# Rove beetles of the genus *Quedius* (Coleoptera, Staphylinidae) of Russia: a key to species and annotated catalogue

**DOI:** 10.3897/zookeys.847.34049

**Published:** 2019-05-17

**Authors:** Maria Salnitska, Alexey Solodovnikov

**Affiliations:** 1 Department of Entomology, St. Petersburg State University, Universitetskaya Embankment 7/9, Saint-Petersburg, Russia St. Petersburg State University Saint-Petersburg Russia; 2 Natural History Museum of Denmark, Zoological Museum, Universitetsparken 15, Copenhagen 2100, Denmark Natural History Museum of Denmark Copenhagen Denmark

**Keywords:** Palearctic, faunistics, systematics, *Microsaurus, Raphirus, Distichalius, Velleius*

## Abstract

This paper is the first inventory of the fauna of the rove beetle genus *Quedius* in the Russian Federation. It provides an annotated catalogue of 88 species of *Quedius* currently recorded from Russia, based on several collections and a critical evaluation of all earlier published records. All species are listed with a summary of their overall distribution and bionomics. Species distributions within Russia are given as lists of regions where they occur with references to the respective source collections or publications which any record is based on. For that, the territory of Russia is divided into 40 regions that mostly follow the administrative division of the country. The annotated catalogue is supplemented by a well-illustrated identification key to all species and a concise checklist in form of an easily visualized table. *Quediusfusus* Cai & Zhou, 2015, *Quediushumosus* Solodovnikov, 2005, and *Quediuslundbergi* Palm, 1973 are recorded from the territory of Russia for the first time. Based on an analysis of literature and available material, records of *Quediuscincticollis* Kraatz, 1857, *Quediushumeralis* Stephens, 1832, *Quediusmaurorufus* (Gravenhorst 1806), *Quediusnemoralis* Baudi de Selve, 1848, *Quediusnigrocaeruleus* Fauvel, 1876, and *Quediuspicipes* (Mannerheim, 1830) from Russia are considered doubtful. The distribution of *Quediusbrachypterus* Coiffait, 1967, described from the ‘Caucasus’, remains ambiguous and its presence in Russia is unlikely. The identity of *Quediusfulvipennis* Hochhuth, 1851 from ‘Dahuria’ remains unknown, pending examination of the type material. For *Quediuscitelli* Kirschenblatt, 1933 a lectotype is designated. For that species and *Q.sofiri* Khachikov, 2015 illustrations of the aedeagi are provided for the first time. The paper stresses the currently poor state of knowledge of the *Quedius* diversity in Russia and provides a platform for its improvement, which should begin with a large-scale sampling program, especially in Siberia and Far East.

## Introduction

With more than 700 species (Herman 2001; Schülke and Smetana 2015) the mainly Holarctic genus *Quedius* Stephens, 1829 is one of the largest among rove beetles (family Staphylinidae) and insects as a whole. *Quedius* are very common inhabitants of the forest leaf and log litter, but they can also be found in other ground-based debris of open landscapes. Some species occur in mammal and bird burrows and nests, in the nests of ants and other wasps, or they are highly adapted to hypogean microhabitats. The species of *Quedius* strongly vary in their landscape and microhabitat preferences, ecological tolerance and, as a result, in the types of their distributions. All these characteristics make *Quedius* commonly encountered beetles and a good model for ecological and biogeographic studies.

Almost the entire diversity of *Quedius* is confined to the Palearctic region (Schülke and Smetana 2015; Smetana 2017) where the largest area is covered by the territory of the Russian Federation. Historically, the main focus of explorations of the Palearctic fauna, including studies of *Quedius*, has been its European part, while the rich and unique faunas of Asia were studied only patchily or remained unexplored. In the last decades we have witnessed a growing interest in the Chinese *Quedius* (Smetana 2017) and, recently, the Middle Asian fauna has been revised (Salnitska and Solodovnikov 2018a, b). With all this progress, the *Quedius* of Russia became a very obvious knowledge gap. As can be seen even from patchy recent publications (Solodovnikov and Hansen 2016; Salnitska and Solodovnikov 2018a; Smetana and Shavrin 2018), the geographically vast and diverse Russia hides numerous *Quedius* species which have not been recorded yet, or are even new to science. To facilitate the badly needed exploration of Russian *Quedius*, we here make a synthesis of the current knowledge of this group within the Russian borders. It aims to structure and summarize all existing literature and the main collections of Russian *Quedius* specimens to assess the fauna, define the largest knowledge gaps and provide an easy platform for further research.

**Figure 1.**
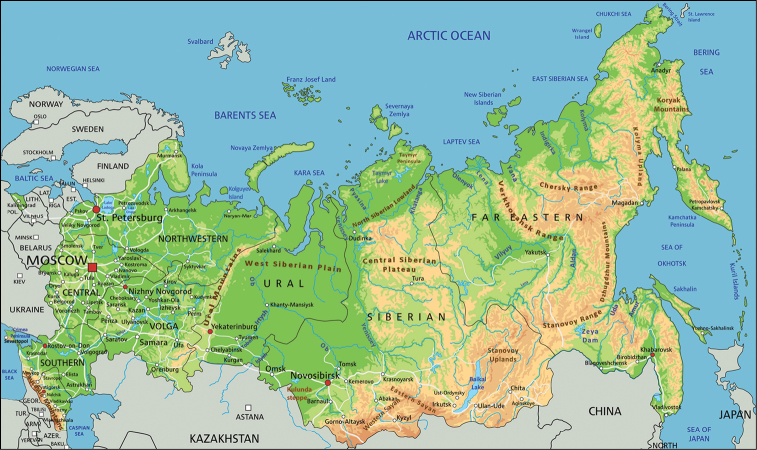
Physical geography map of the Russian Federation.

Russia is a country stretching through a large and extremely diverse geographic area (Fig. 1) that includes diverse biomes from arctic deserts to subtropics. Even though a considerable part of Russia is located within the less biologically diverse polar or boreal regions, its overall species diversity is high because of the multiple terrestrial ecosystems, landscapes and habitats meeting here.

With respect to *Quedius*, very little is known about the Russian fauna. It is very difficult to initiate and advance studies in this direction because even the existing scarce taxonomic and faunistic literature relevant to *Quedius* in Russia is very fragmented, incomplete and, at most, applicable only to smaller regions of the country. So far, reliable work can be done only by somebody with many years of experience. There is not a single publication which could serve as an easy ‘kick start’ for taxonomic or faunistic work on *Quedius* in Russia by the broader community of entomologists. All existing catalogues that cover Russia provide little detail specifically for its territory. For example, the catalogue of rove beetles of the former USSR and adjacent regions in Tikhomirova (1973), a breakthrough for its time and listing 177 species of *Quedius*, is now greatly outdated in terms of taxonomy and coverage. The important Staphylinidae catalogues for the Palearctic region (Schülke and Smetana 2015) or the entire world (Herman 2001) consider Russia only very superficially. For example, in Schülke and Smetana (2015) the territory of Russia is subdivided only into six very large regions and the distribution of each species looks like an enumeration (and thus a very rough outline) of these regions without their underlying literature records. Although Herman (2001) provided an extremely helpful summary of all main references from 1758 to 2001 for each species listed in his catalogue, information relevant for species in Russia is incomplete there. It is even more difficult to identify material collected in Russia. There were only two incomplete and now greatly outdated keys for the *Quedius* fauna of the European part of Russia: one with only eleven species (Jacobson 1905; reproduced and updated in Bogdanov-Katkov, 1930) and the other with 50 species (Kirshenblat 1965). Otherwise, identification of Russian *Quedius* specimens could be attempted with the aid of modern keys for Central European fauna (Solodovnikov 2012b), the outdated monograph of the West Palearctic Staphylinidae (Coiffait 1978), or the recent monograph of *Quedius* of China (Smetana 2017). Needless to say that none of these keys can really work for the Russian fauna as a whole because at most one can key out only widespread species or those that occur in the immediate neighborhood to the geographic coverage of these keys. The absence of good synoptic collections of *Quedius* that would be distributed in Russia, or at least accessible at the main Russian institutions, contributes to the impediment. All this motivated us to compile the present work, which is an identification key and an annotated catalogue of all species of *Quedius* that we have found in the fauna of Russia thus far, based on an exhaustive literature survey and examination of the main collections herein and abroad.

## Materials and methods

Our publication is based on literature data and examination of specimens from several collections abbreviated as follows:

**CNC**Canadian National Collection, Ottawa, Canada (A Brunke)

**ISEA** Institute of Systematics and Ecology of Animals, Siberian Branch of the Russian Academy Sciences, Novosibirsk, Russia (R Dudko)

**LUOMUS**Finnish Museum of Natural History, Helsinki, Finland (J Muona, J Mattila)

**LUOMUS**Muséum national d’Histoire naturelle, Paris, France (A Taghavian-Azari)

**NHMD** Natural History Museum of Denmark at the University of Copenhagen (includes the Zoological Museum formerly known as ZMUC), Copenhagen, Denmark (A Solodovnikov)

**ZIN**Zoological Institute, Russian Academy of Science, Saint-Petersburg, Russia (BA Korotyaev)

**ZMMU** Zoological Museum of Moscow University, Moscow, Russia (AA Gusakov)

**cAle** Private collection of S Alekseev, Kaluga, Russia

**cGon** Private collection of A Gontarenko, Odessa, Ukraine

**cKur** Private collection of S Kurbatov, Moscow, Russia

**cRyv** Private collection of A Ryvkin, Moscow, Russia

**cSha** Private collection of A Shavrin, Daugavpils, Latvia

**cSme** Private collection of A Smetana, Ottawa, Canada

To gather original distributional and reference data for this publication we used a custom made database implemented in Microsoft Access 2010. Our publication consists of three interconnected parts: 1) identification key to all *Quedius* species that occur in Russia; 2) annotated species list arranged by subgenera and alphabetically within each subgenus; and 3) a brief summary of distribution, abundance and source of data for each species in Russia in tabular format, with species arranged alphabetically across the entire genus.

### Russia and its division for the catalogue

The Russian Federation (Fig. 1) extends through ca. 17 million square kilometers from the river Pededze [57.518N, 27.352E] (between Estonia and Pskov Province of Russia) in the west to Cape Dezhnev [66.083N, 169.653E] (Chukotka Autonomous District) in the East, and from Cape Chelyuskin [77.723N, 104.259E] (Krasnoyarsk Territory) in the north to the south of Bazarduzu Mountain [41.185N, 47.782E] (Dagestan Republic) in the south. Kaliningrad Province, including its numerous small islands in the Baltic Sea, is the westernmost enclave separated from the rest of the country by Lithuania, Latvia, and Estonia. While the Crimea Republic is separated from the rest of Russia by the south-western part of Ukraine and the Kerch Strait. From north to south, Russia covers several climate zones from the arctic to subtropics. From west to east it is extended from the Baltic Sea through Siberian plains and Far East mountains to the Pacific Ocean. Russian terrain consists of very diverse forms of relief ranging from high mountains such as Caucasus with Elbrus Mountain as the highest point in Russia at 5642 m, through Ural, Altai, Sayan, Sikhote-Alin, Verkhoyansk, and Chersky ranges, to the plains and lowlands such as European, west Siberian and north Sakhalin plains, or north Russian, Pskov, Cis-Kuban, Cis-Ilmen, Abyisk lowlands, or Kuznetsk Depression, and others.

Finding a system of subdivision for such a large and diverse area as Russia that is suitable for cataloguing purposes is complicated. Normally it is better to visualize species ranges via some biogeographic division reflecting natural geographic units or landscapes (Kryzhanovsky et al. 1995). Such an approach is feasible in the case of well-studied faunas, with clear distributions and bionomics of the species, as well as some widely agreed biogeographic scheme. Unfortunately, rove beetles and *Quedius* in particular are very poorly explored, while a widely agreed upon and detailed biogeographic division of Russia is even more of a problem. In our case, the use of political administrative regions with unambiguous borders, standardized across various maps, is a viable solution. Additionally, records from local faunistic publications are usually restricted to such regions. Therefore, accepting them for our catalogue also simplifies the inventory of these publications. However, political divisions, especially in Russia, comprise units that are not always geographically homogeneous and may consist of very different, sometimes contrasting geographic regions. A large river, a mountain ridge, or another natural barrier may cut a certain large administrative region as the Lena River does in Yakutia (Sakha) Republic, or Kulunda steppe in Altai Territory. On the contrary, some geographically uniform areas may be divided between several administrative regions such as the Ural Mountains, stretching through Yamalo-Nenets and Khanty-Mansi Autonomous districts, Tuymen, Sverdlovsk, and Chelyabinsk provinces. Moreover, the denser populated European part of Russia is fractured into numerous and small administrative regions such as Orel Province or Mordovia Republic, whereas poorly populated Siberia consists of very large regions such as Yakutia (Sakha) Republic or Evenk Autonomous District.

**Figure 2.**
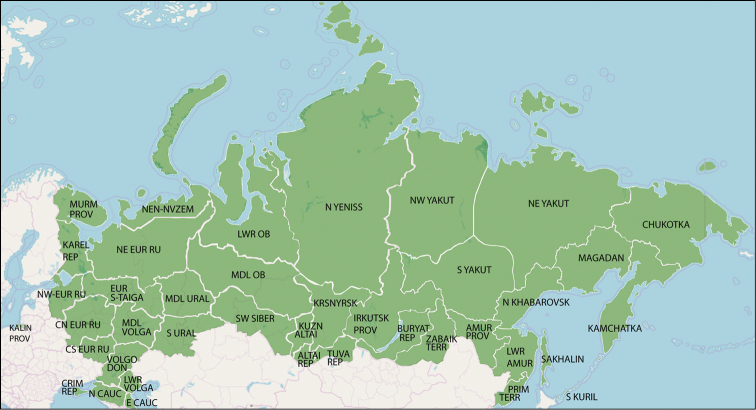
Regions used for the division of the Russian Federation in this publication.

To overcome these problems, we here divide Russia as in the Catalogue of Lepidoptera of Russia (Sinev 2008), which is mainly based on administrative political regions with minor amendments following geographic considerations (Fig. 2). In particular, groups of smaller geographically similar regions of European Russia are merged together, while some Siberian regions are subdivided in accordance with geographic barriers. For the purposes of our catalogue, the composition of some administrative regions was changed according to geography, as follows: Arkhangelsk Province is divided into two regions, one consisting of Nenets Autonomous District with the Novaya Zemlya archipelago and the other covering the rest of its continental area. Tyumen Province is divided in two regions, west and east of Tobol and Irtysh rivers, respectively. Altai Territory is divided into Kulunda steppe and the rest. Krasnoyarsk Territory, apart from Taymyr and Evenk Autonomous Districts, is divided into two regions, one north and one south of Sym River. Similarly, Khabarovsk Territory is divided in two regions, one north and one south of Uda River. Yakutia Republic is divided in three regions, North-Western, North-Eastern and Southern Yakutia, based on the Verkhoyanskiy Range watershed and the Rivers Vilyuy and Aldan, respectively.

As a result, the Russian Federation here is divided into 40 regions abbreviated and listed alphabetically as follows. Numbers correspond to the respective position of the regions in Table 1 where they are arranged according to their location in Russia, from north to south and from west to east:

**ALTAI REP (24)** Altai Republic

**AMUR PROV (36)** Amur Province

**BURYAT REP (27)** Buryatia Republic

**CHUKOTKA (32)** Chukotka: Chukotka Autonomus District, Koryak district, Wrangel Island

**CN RU (8)** Central Northern European Russia: Tver, Smolensk, Yaroslavl, Moscow, Kaluga, Bryansk, Tula, Ryazan, Vladimir, and Ivanovo provinces

**CRIM REP (13)** Crimea Republic

**CS RU (9)** Central Southern European Russia: Kursk, Lipetsk, Tambov, Orel, Belgorod and Voronezh provinces

**E CAUC (15)** Eastern Caucasus: Chechnya and Dagestan republics

**EUR S-TAIGA RU (7)** European Southern taiga Russia: Vologda, Kostroma, and Kirov provinces, Udmurt Republic

**IRKUTSK PROV (26)** Irkutsk Province

**KALIN PROV (1)** Kaliningrad Province

**KAMCHATKA (34)** Kamchatka: the Kamchatka Peninsula (part of Kamchatka Territory), Commander Islands (belong to Kamchatka Territory) and northern Kuril Islands south to Urup strait (belong to Sakhalin Province)

**KAREL REP (3)** Karelia Republic

**KRSNYRSK (22)** Krasnoyarsk: south of Krasnoyarsk Territory, Khakassia Republic

**KUZN ALTAI (23)** Kuznetsk-Altai: Kemerovo Province, Altai Territory (without Kulunda Steppe)

**LWR AMUR (37)** Lower Amur: southern part of Khabarovsk Territory, Jewish Autonomous Province

**LWR OB (18)** Lower Ob: Yamalo-Nenets Autonomous District

**LWR VOLGA (12)** Lower Volga: Astrakhan Province, Kalmykia Republic

**MAGADAN PROV (33)** Magadan Province

**MDL OB (19)** Middle Ob: Khanty-Mansi Autonomous District, Tomsk Province

**MDL URAL (16)** Middle Ural: Perm Territory, Sverdlovsk Province and western part of Tyumen Province

**MDL VOLGA (10)** Middle-Volga Nizhny Novgorod, Penza, Ulyanovsk and Samara provinces, Tatarstan, Mari-El, Chuvashia and Mordovia republics

**MURM PROV (2)** Murmansk Province

**N CAUC (14)** Northern Caucasus: Krasnodar and Stavropol territories, Adygea, Kabardino-Balkaria, Karachay-Cherkessia, North Ossetia–Alania and Ingushetia republics

**N KHABAROVSK (35)** Northern Khabarovsk (northern part of Khabarovsk Territory to the Uda River in the south)

**N YENISS (21)** Northern Yenisei: Taymyr and Evenk Autonomous Districts, northern part of Krasnoyarsk Territory

**NE RU (6)** North-Eastern European Russia: Arkhangelsk Province (without Nenets Autonomous District and Novaya Zemlya archipelago), Komi Republic

**NE YAKUT (30)** North-Eastern Yakutia (Sakha) Republic

**NEN–NVZEM (5)** Nenets–Novaya Zemlya: Nenets Autonomous District, Novaya Zemlya archipelago

**NW RU (4)** North-Western European Russia: Leningrad, Novgorod and Pskov provinces

**NW YAKUT (29)** North-Western Yakutia (Sakha) (in the east up to Verkhoyanskiy range watershed)

**PRIM TERR (40)** Primorsky Territory

**S KURIL (39)** Southern Kuril: southern Kuril islands (Kunashir, Iturup, Urup, Shikotan, and other islands of Lesser Kuril Chain, all belong to Sakhalin Province)

**S URAL (17)** Southern Ural: Bashkortostan Republic, Orenburg, Chelyabinsk, and Kurgan provinces

**S YAKUT (31)** Southern Yakutia: Yakutia (Sakha Republic) south of Vilyuy and Aldan rivers

**SAKHALIN (38)** Sakhalin Island (belongs to Sakhalin Province)

**SW SIBER (20)** South-Western Siberian: Tyumen Province (eastern part), Omsk and Novosibirsk provinces, Altai Territory (eastern part: Kulunda Steppe)

**TUVA REP (25)** Tuva Republic

**VOLGO-DON (11)** Volgo-Don: Saratov, Volgograd, and Rostov provinces

**ZABAIK TERR (28)** Zabaikalsky Territory

### History of the study of *Quedius* of Russia

The first mentions of species of the genus *Quedius* from an area that included the territory of modern Russia belong to Hochhuth (1849–1862) who published several works on the fauna of the Caucasus (1849) and “Russlands” (1851, 1862). The first descriptions of new species from the territory of Russia were confined to the unique and rich fauna of the north-western Caucasus (Eppelsheim 1878a, b, 1889; Roubal 1911). Among other pioneering studies, Fauvel (1875), Eppelsheim (1886, 1887), Bernhauer (1902), and Roubal (1914, 1929) described new species from Siberia and the Russian Far East.

Throughout the rest of the 20^th^ and the beginning of the 21^st^ centuries, the amount of taxonomic publications that touched upon *Quedius* of Russia significantly grew and included many species described from the Russian parts of the Caucasus (Coiffait 1967; Solodovnikov 2002, 2004), Altai Mountains (Coiffait 1969; Salnitska and Solodovnikov 2018a, b), Siberia (Kirschenblatt 1933; Coiffait 1975; Smetana 1978b, 1995), or Far East (Solodovnikov and Hansen 2016; Smetana 2003; Smetana and Shavrin 2018). In addition to these taxonomic publications, there are faunistic publications accumulated over decades. Usually these cover local faunas within political borders of various larger or smaller regions of Russia (Shilov 1975; Shavrin 2000; Goreslavets et al. 2002; Nikitsky and Schigel 2004; Dedyukhin 2005; Pavlov 2005; Shulaev and Bogdanov 2008; Ryabukhin 2008, 2010; Kolesnikova and Molkov 2009; Semenov 2009; Kovalev et al. 2011; Dorofeev 2013; Goreslavets 2014, 2016; Kolesnikova 2015; Pushkin and Minav 2015; Babenko 2016; Ruchin 2016; Voitenkova 2016 etc.), other larger or smaller geographical territories of any kind (Koval 1961; Boháč 1986; Babenko 1991; Solodovnikov 1998; Grebennikov 2001; Kolesnikova 2008, 2012; Kolesnikova and Konakova 2010; Alekseev and Shapoval 2012; Semenov et al. 2013; Alekseev 2014; Troshkova and Troshkov 2014; Chernov et al. 2014; Lobkova and Semenov 2017 etc.), as well as nature reserves and protected areas (Veselova and Ryvkin 1991; Uhova 2001; Ermakov 2003; Kolesnikova and Taskaeva 2003; Koryakin 2004; Goreslavets 2010; Pirugin 2010; Semenov 2010; Babenko and Nuzhnykh 2014; Dorzhieva and Khobrakova 2014; Aiydov 2015; Psarev 2015; Semenov 2016, 2017; Semenov et al. 2014, 2015 etc.). Often these papers were published in various local, hard-to-access outlets, and the quality of their underlying species identifications is variable.

**Figure 3.**
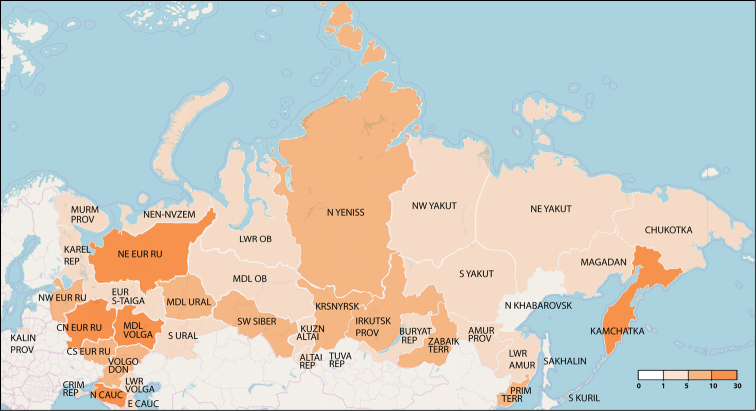
Summary statistics of the published records of *Quedius* in Russia. Numbers at the color bar indicate number of literature records, respectively.

**Figure 4.**
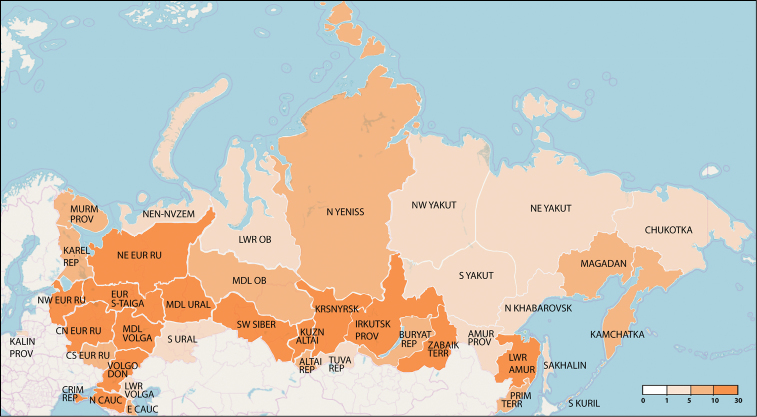
Summary statistics of the diversity of *Quedius* species in various regions of Russia. Numbers at the color bar indicate number of species, respectively.

Overall, the current knowledge about *Quedius* of Russia is very fragmented, both taxonomically and geographically and often it is hidden in the publications of a more inclusive scope, covering all Staphylinidae or even Coleoptera. Finally, for some regions of Russia, publications, or even collected material are limited to non-existent (Figs 3, 4).

## Taxonomy

The subdivision of the genus *Quedius* into subgenera is used here according to Schülke and Smetana (2015). It is noteworthy to mention that the genus *Quedionuchus* recently reinstated to this level (Brunke and Solodovnikov 2013) is not included in this catalogue. Within subgenera, we list species alphabetically. Since the territory of Russia is very extensive, it is impossible to use any species groups developed only for local faunas from adjacent countries such as China (Smetana 2017).

At the species level, there are a number of taxonomic problems pending more detailed studies as well. For example, *Quediusumbrinus* displays very strong morphological variation suggesting a complex of more than one species. On the other hand, species limits are not clear among some described species, to mention *Q.sublimbatus* and *Q.arcticus* pair, or the *Q.boops*-group as examples. In case of *Q.sublimbatus* and *Q.arcticus*, we follow their conventional synonymy. Our accepted concept of *Quediusboops*, *Q.boopoides*, and *Q.paraboops* also needs further study. These three species are indistinguishable from each other by characters of external morphology. Genitalic differences are subtle, subject to variation and, together with geographic distribution, are interpreted here as follows. Two species, *Q.boops* and *Q.boopoides*, occur sympatrically from Europe to Siberia, but *Q.boopoides* gradually becomes rare from the west to the east of its range, which does not reach the Far East. Meanwhile, *Q.boops* is present in the Far East, with its easternmost record known from the Lower Amur region. At the same time, *Q.paraboops* is known only from Siberia and Far East, but its western form that occurs in Krasnoyarsk and Tuva regions appears as a gradual transition between this species and *Q.boops*. Future examination of a larger amount of material using rigorous methods of molecular and morphometric species delimitation should bring more clarity about species limits in the *Q.boops*-group. Other species of the Russian fauna also pose various taxonomic problems, perhaps of a lower severity. In those cases some assumptions or preliminary conclusions are discussed in the respective ‘Notes’ section. In general, we deliberately avoided any taxonomic changes and nomenclatural acts here, pending their proper justification and implementation in the separate publications.

### Identification keys

These are traditional dichotomous keys that also include a succinct summary of the most important diagnostic and biological features for each species. Often distributions or bionomics may be as helpful as morphology, especially for identification of closely related species. The overall structure of the key and some aedeagus illustrations are adopted from Solodovnikov (2012). Species whose presence in the Russian fauna is strongly ambiguous are placed in square brackets.

### Key to subgenera of *Quedius* of Russia

**Table:** 

1	Elytra densely covered by setiferous punctures, interspaces between punctures smaller or slightly larger than diameter of punctures	**2**
–	Elytra with sparse setiferous punctures, interspaces distinctly larger than diameter of punctures. (Fig. 5A)	**Subgenus Distichalius Casey, 1915**
2	Anterior margin of labrum with deep emargination or distinct notch in the middle so that labrum appears bilobed. Body size variable	**4**
–	Anterior margin of labrum entire so that labrum never bilobed or emarginated in the middle. Habitus as in Fig. 5B–E	**3**
3	Large species with body length not smaller than 9 mm	**Subgenus Quedius Stephens, 1829**
–	Smaller species, body not longer than 7 mm	**Quedius (Rahirus) jenisseensis** 1
4	Eyes in most cases small or moderate in size, slightly longer to distinctly shorter than temples (Figs 5F, 6A–D). Vertex (one side) with two basal punctures postero-medially from posterior frontal puncture. Postero-lateral areas of pronotum somewhat explanate in most cases	**5**
–	Eyes large and convex, always longer than temples. Vertex (one side) with one basal puncture postero-medially from posterior frontal puncture. Postero-lateral areas of pronotum not explanate. Habitus as in Figs 6E, F; 7; 8A, B	**Subgenus Raphirus Stephens, 1829**
5	Smaller species 4.5–14.0 mm. Antennae not serrate. Pronotum mostly not transverse and laterally only slightly explanate, with marginal setae situated at or very close to pronotal margins	**Subgenus Microsaurus Dejean, 1833**
–	Large and robust species 15.0–24.0 mm. Antennae strongly serrate. Pronotum distinctly transverse, laterally strongly explanate, with marginal setae situated at notable distance from pronotal margins	**Subgenus Velleius Leach, 1819**

### Key to Russian species of the subgenus Distichalius Casey, 1915

**Table:** 

1	Elytra entirely reddish, sometimes darkened at suture. Aedeagus (Fig. 9A–D): (in dorsal or ventral view) median lobe distinctly bilobed at apex (Fig. 9D). Body length 4.9–6.0 mm. Known from Russian Far East (Schülke and Smetana 2015)	***Q.japonicus* Sharp, 1874**
–	Elytra black or brownish-black, sometimes lighter at suture (exceptionally, elytra can be pale in some specimens of *Q.cinctus*). Aedeagus: median lobe never bilobed at apex	**2**
2	Larger species: body length 7.5–8.5 mm. Lateral outline of head gradually converging towards neck behind eyes	**3**
–	Smaller species: body length 5.5–7.5 mm. Lateral outline of head parallel-sided immediately behind eyes and then broadly rounded and converging towards neck	**4**
3	Aedeagus (Fig. 9E–G): paramere (in dorsal or ventral view) wide and fusiform, strongly narrowed at middle, (from underside) with one longitudinal band of peg setae along midline (Fig. 9E, G), (in lateral view) apically distinctly protruding over apex of median lobe (Fig. 9F). Hitherto known from the original description (China, Beijing, Cai and Zhou 2015) and one record from Amur Province in the Russian Far East	***Q.fusus* Cai & Zhou, 2015**
–	Aedeagus (Fig. 9H–J): paramere (in dorsal or ventral view) lanceolate, slightly narrowed in the middle, (from underside) with two rows of sensory peg setae arranged close to apical margins (Fig. 9H, J); (in lateral view) paramere vaguely protruding over level of apex of median lobe (Fig. 9I). Distributed in the West Palearctic; common and polytopic species. In Russia, known only from its European part	***Q.cinctus* (Paykull, 1790)**
4	Larger species 6.0–7.5 mm (Fig. 5A). Aedeagus (Fig. 9K–M): (in lateral view) paramere slightly or significantly protruding over level of apex of median lobe. Montane species distributed in Western and Central Caucasus and northern Turkey. In Russia, known only from the Northern Caucasus region	***Q.minor* Hochhuth, 1849**
–	Smaller species 5.5–6.0 mm. Aedeagus (Fig. 9N–P): (in lateral view) paramere not quite reaching to apex of median lobe. Known only from Kamchatka peninsula	***D.kamchaticus* Smetana, 1976**

### Key to Russian species of the subgenus Quedius Stephens, 1829

**Table:** 

1	Scutellum impunctate, glabrous	**2**
–	Scutellum punctate, setose	**3**
2	Aedeagus (Fig. 9Q–S): (in parameral view) apical part of paramere acuminate, with lateral margins sinuate, rows of sensory peg setae, in their basal half, extended medially from lateral margins (Fig. 9Q, S); (in ventral view) lateral margins of median lobe apically not visible from under paramere (Fig. 9R). Elytra usually black, but occasionally partly or entirely reddish. At least first antennal segments slightly darkened (except if teneral). Body length 10.0–15.0 mm. Common West Palearctic species reaching Northern Yenissey and Krasnoyarsk regions, as well as Irkutsk Province and Buryatia Republic	***Q.fuliginosus* (Gravenhorst, 1802)**
–	Aedeagus (Fig. 9T–V): (in parameral view) apical part of paramere gradually narrowing apicad; rows of sensory peg setae, in their basal half, extended more laterally, closer to parameral lateral margins (Fig. 9T, V); (ventral view) lateral margins of median lobe apically visible from under paramere (Fig. 9U). Elytra usually black. First antennal segments not even slightly darkened. Body length 10.0–15.0 mm. Widespread in Europe, can be found together with *Q.fuliginosus*; recorded from Middle Asia. In Russia known only from the European part	***Q.curtipennis* Bernhauer, 1908**
3	Frons with additional setiferous punctures between anterior frontal punctures. Aedeagus (Fig. 10A–D): median lobe (in dorsal view) with pair of weak lateral teeth, without a pair of medial teeth, and short apical medial carina (Fig. 10D). Body length 10.0–16.00 mm. Habitus as in Fig. 5B. Widespread in West Palearctic, rarer in the north. In Russia, only known only from the European part	***Q.levicollis* Brullé, 1832**
–	Frons without additional setiferous punctures between anterior frontal punctures. Aedeagus: median lobe (in dorsal view) apically without two well developed lateral teeth, with a pair of medial teeth, without apical medial carina (e.g., Fig. 10L, I)	**4**
4	Elytra shortened, distinctly shorter than pronotum, obviously brachypterous species without whitish apical seam on abdominal tergite VII. Habitus as in Fig. 5C. Smaller: body length 7.5–9.0 mm. Aedeagus as in Fig. 10E–H. Wide spread Russian species, known from south-eastern Siberia to Far East	***Q.sundukovi* Smetana, 2003**
–	Elytra normal, not shortened, about as long as pronotum. Species with whitish apical seam on abdominal tergite VII. Larger: body length 8.6–12.5 mm	**5**
5	Body brown, with reddish elytra. Aedeagus (Fig. 10I–L): (in lateral view) apex of paramere pointing ventral, away from median lobe in the form of a small hook (Fig. 10J). Body length 12–14 mm. Common in south-eastern part of West Palearctic. In Russia known from the Eastern and Northern Caucasus	***Q.vicinus* Ménétriés, 1832**
–	Body black, or at most brownish (Fig. 5D, E). Elytra black or brown, rarely reddish. Aedeagus (in lateral view): apex of paramere straight, not pointing ventrad, away from median lobe (Fig. 10N)	**6**
6	Antennae light or at least first two to three antennomeres distinctly paler than remaining antennomeres	**7**
–	Antennae dark including first two to three antennomeres	**8**
7	Aedeagus (Fig. 10M–P): (in lateral view) C-shaped sclerite of internal sac with spine-like basal extension (Fig. 10N); (underside) with rows of sensory peg setae located in the middle of paramere and closer to each other (Fig. 10O). Body length 9.5–13.0 mm. Common in West Palearctic with the eastern limit stretching through Northern Yenissey and Krasnoyarsk regions in Russia. In Russia, more common in the northern and central regions and becoming rare towards the south	***Q.molochinus* (Gravenhorst, 1806)**
–	Aedeagus (Fig. 10Q–T): (in lateral view) C-shaped sclerite of internal sac without spine-like basal extension; paramere (underside) with rows of peg setae located closer to margins of paramere and further from each other (Fig. 10S). Body length 10.0–13.0 mm. Widespread in the south of the West Palearctic. In Russia, known from southern regions of the European part	***Q.meridiocarpathicus* Smetana, 1958**
8	Elytra most often brownish. Aedeagus (Fig. 10U–X): (in ventral or dorsal view) median lobe with attenuate part of its apex shorter and with less pronounced teeth near apex (Fig. 10X); (in lateral view) internal sac with main sclerite thicker, less obviously C-shaped (Fig. 10V); (parameral view) paramere at middle relatively broader, on underside with apical rows of sensory peg setae situated in the middle of paramere and largely confluent from apex to about half of their extension basad (Fig. 10U, W). Habitus as in Fig. 5D. Body length 9.0–12.0 mm. Widespread in northern and central parts of the West Palearctic. In Russia, recorded only from Crimea Republic and Volgo-Don regions	***Q.balticus* Korge, 1960**
–	Elytra most often black or dark brown. Aedeagus: (in ventral or dorsal view) median lobe with attenuate part of its apex (Fig. 11D, H) elongate and with more pronounced teeth near apex; (in lateral view) internal sac with thin, obviously C-shaped main sclerite; (parameral view) paramere narrower at middle, on underside with apical rows of sensory peg setae well separated, situated close to margins of paramere	**9**
9	Aedeagus (Fig. 11A–D): (in lateral view) C-shaped sclerite of internal sac distinctly arcuate, median lobe with larger, distinctly visible subapical teeth (Fig. 11B). Northern European species, with unclear distribution limits in Russia	***Q.subunicolor* Korge, 1961**
–	Aedeagus (Fig. 11E–H): (in lateral view) C-shaped sclerite of internal sac only slightly curved, median lobe with smaller, hardly visible subapical teeth (Fig. 11F). Known from few localities in the Altai Mountains	***Q.altaicus* Korge, 1962**

### Key to Russian species of the subgenus Microsaurus Dejean, 1833

**Table:** 

1	Scutellum punctate, setose, even if sometimes with very few punctures	**2**
–	Scutellum completely impunctate, glabrous (sometimes with irregular surface sculpture, but never with setiferous punctures)	**7**
2	Distinctly larger species, body length 11.0–14.0 mm. Head, pronotum and elytra dark brown to blackish, abdomen more or less reddish-brown. Antennomeres not serrate, of moniliform shape. Aedeagus as in Fig. 11I–K. Widespread in Europe, but not common. In Russia, known only from Middle Volga region	***Q.truncicola* Fairmaire & Laboulbène, 1856**
–	Small species, body length 4.5–6.8 mm. Aedeagus and habitus different	**3**
3	Body variously yellowish-brown to brown. Elytra (measured at sides, from shoulder) distinctly longer than wide, usually with distinctly or vaguely paler margins	**4**
–	Body piceous black to brown. Elytra (measured at sides, from shoulder) as long as wide or slightly shorter than wide, usually uniformly colored	**6**
4	Eyes small and flat, distinctly shorter than temples. Aedeagus (Fig. 11L–N): (in lateral view) apex of median lobe broad and distinctly protruding over apex of paramere (Fig. 11M); paramere on underside with about six or less sensory peg setae in each of two longitudinal rows (Fig. 11N). Body length 4.5–5.5 mm. Widely distributed in Europe except Iberian Peninsula. In Russia, known from the European part and from a few regions in south-western Siberia	***Q.microps* Gravenhorst, 1847**
–	Eyes rather large and slightly protruding beyond head contour, as long as, or slightly longer than temples. Aedeagus different	**5**
5	Aedeagus (Fig. 11O–Q): (in lateral view) apex of paramere distinctly protruding over apex of median lobe (Fig. 11P), underside of paramere with ca. 10 sensory peg setae in each of two regular rows situated closer to parameral margins (Fig. 11Q). Body length 5.0–6.5 mm. Widely distributed in Europe. In Russia, known from its southern European part	***Q.infuscatus* Erichson, 1840** 2
–	Aedeagus (Fig. 11R–T) : (in lateral view) apex of paramere not or slightly protruding over apex of median lobe (Fig. 11S), underside of paramere with ca. 30 sensory peg setae in each of two wide rows of irregularly scattered sensory peg setae situated further from parameral margins (Fig. 11S). Body length 7.5–8 mm. Currently known from Sweden; here newly recorded from Tuva Republic of Russia	***Q.lundbergi* Palm, 1972**
6	Larger species, body length 6.8 mm. Aedeagus as in Fig. 11U, V. Known only from the type locality in Amur Province of Russia	***Q.amurensis* Smetana, 2018**
–	Smaller species, body length 5.9 mm. Aedeagus as in Fig. 11W–Y. Known from Turkey and the type locality in Rostov Province of Russia	***Q.sofiri* Khachikov, 2005**
7	Microsculpture of head and pronotum consisting of isodiametrical meshea, never of waves and micropunctation. Head and pronotum dull, not glossy. Head dark brown, pronotum reddish-brown to brown with paler sides; elytra, appendages and apical parts of abdominal tergites reddish to pale brown. Aedeagus as in Fig. 12A–C. Body length 9.0–10.0 mm. Widely distributed in the Europe and European part of Russia, reaching south-western Siberia. Usually associated with mole nests (Potockaja 1967; Osella and Zanetti 1975; Nowosad 1990)	***Q.longicornis* Kraatz, 1857 8**
–	Microsculpture of head and pronotum consisting of transverse waves and often with micropunctation, never isodiametric. Head and pronotum more or less glossy. Habitus and aedeagus different	**8**
8	Elytra brownish, of the same or very similar coloration as rest of the body. Eyes very small, temples 1.9–2.5 times as long as eyes. Elytra slightly or distinctly shorter than pronotum. Distinctly brachypterous species without whitish apical seam on abdominal tergite VII	**9**
–	Elytra dark brown or even blackish as rest of body, or contrasting with darker body. (If elytra black as rest of body, see *Q.nigrocaeruleus*). Eyes larger, temples ca. 0.5–1 times as long as eyes. Elytra longer than, or as long as pronotum. Apical seam on abdominal tergite VII always present	**10**
9	Head narrow, pronotum with distinctly pronounced posterior angles and lateral contours strongly narrowing anteriad. Aedeagus (Fig. 12D–F): median lobe (in lateral view) with wide and broad apex (Fig. 12E); paramere with slight apical emargination and sensory peg setae (ca. 8–10) forming compact groups at apex only (Fig. 12F). Known only from the type locality in Sikhote-Alin Mountains in Khabarovsk Territory of Russia	***Q.roma* Solodovnikov & Hansen, 2016**
–	Head wider, pronotum with less pronounced posterior angles and lateral contours less narrowed anteriad. Aedeagus (Fig. 12G–I): median lobe (in lateral view) with narrower apex (Fig. 12H); paramere with deeper apical emargination and sensory peg setae (ca. 10–12) extending to parameral lateral margins (Fig. 12I). Known only from the type locality in Altai Mountains in Altai Republic of Russia	***Q.repentinus* Salnitska & Solodovnikov, 2018**
10	Pronotum with four setiferous punctures in dorsal row (check both dorsal rows because occasionally the basalmost puncture may be reduced or lost in one row). Body testaceous brown to blackish, pronotum and especially elytra and apical margins of abdominal tergites often paler than rest of the body (Fig. 5F). Aedeagus as in Fig. 12J–L. Body length 7.0–8.0 mm. Transpalearctic, in Russia from Central Northern European region to Kamchatka	***Q.tenellus* (Gravenhorst, 1806)**
–	Pronotum with two or three setiferous punctures in dorsal row (check both dorsal rows because occasionally the basalmost puncture may be reduced or lost in one row; also count the foremost puncture which can be very close to anterior margin of pronotum and slightly laterad from other punctures of dorsal row). Aedeagus different	**11**
11	Pronotum with two setiferous punctures in dorsal row (Fig. 6A, B)	**12**
–	Pronotum with three setiferous punctures in dorsal row (Fig. 6C, D)	**17**
12	Elytra and rest of body black, apical margins of abdominal tergites and appendages vaguely paler. Sublateral rows of punctures on pronotum absent. Aedeagus as in Fig. 12M, N. Body length 8.5–9.0 mm. Known only from the type locality in Irkutsk Province of Russia, where it was collected in the burrow of *Urocitellusundulatus* (Pallas, 1778)	***Q.conviva* Smetana, 2018**
–	Elytra reddish, yellowish, or brown, body black or brown, sometimes pale. Sublateral rows of punctures on pronotum consisting of 1–3 punctures. Aedeagus different	**13**
13	Smaller species, body length 6.0–8.0 mm. Eyes small and flat, distinctly shorter than temples	**14**
–	Larger species, body length 7.0–12.5 mm. Eyes rather long and convex, as long as or slightly longer than temples	**15**
14	Body brown, often with paler elytra and apical margins of abdominal tergites (Fig. 6A). Aedeagus (Fig. 12O–Q): (in parameral view) paramere apically lanceolate, sharpened (Fig. 12O, Q); (in lateral view) median lobe moderately wide, with broad, but distinctly pointed apex (Fig. 12P). Body length 6.0–7.0 mm. Transpalearctic myrmecophilous species. In Russia it can be found from Central Northern European to Lower Amur regions. Widespread, but not common	***Q.brevis* Erichson, 1840**
–	Body black to dark brown, with reddish elytra. Aedeagus (Fig. 12R–T): (in parameral view) paramere apically of rhomboid shape, with rounded apex (Fig. 12R, T); median lobe (in lateral view) wider, with broad apex (Fig. 12S). Hitherto known only from the type locality in Adun-Tshelon range, Chita region of Russia, where it was collected in the burrow of *Spermophilusdauricus* Brandt, 1843	***Q.citelli* Kirschenblat, 1933**
15	Aedeagus: (in parameral view) paramere apically of rhomboid shape, strongly narrowed towards pointed apex, underside sensory peg setae arranged in separate groups situated only along parameral margins (Figs 12X, Z; 13A, C); median lobe (in lateral view) broad with strongly narrowed short apical portion, only slightly protruding over apex of paramere (Figs 12X, 13C). Smaller species 7.0–9.0 mm	**16**
–	Aedeagus (Fig. 12U–W): (in parameral view) paramere apically of rectangle shape, slightly narrowed towards truncate apex, underside with sensory peg setae arranged in one irregular group located more medially and posteriad (Fig. 12U, W); median lobe (in lateral view) narrower with gradually narrowed and elongate apical portion, distinctly protruding over apex of paramere (Fig. 12V). Larger species 10.0–12.5 mm. Distributed in west and south of Central Caucasus, apparently confined to the burrows of *Prometheomysschaposchnikovi* Satunin, 1901	***Q.abdominalis* Eppelsheim, 1878**
16	Aedeagus (Fig. 12X–Z): (in parameral view) apical part of paramere strongly rhomboid with distinct lateral angles, with vaguely bilobed apex, underneath with one longitudinal row of ca. 11–28 peg setae along each of its lateral margins (Fig. 12X, Z); (in lateral view) apex of median lobe strongly narrowed and very short (Fig. 12Y). Habitus as in Fig. 6B. Known only from a wide area in south-eastern Russia, from Irkutsk Province to Southern Kurils	***Q.fasciculatus* Eppelsheim, 1886**
–	Aedeagus (Fig. 13A–C): (in parameral view) apical part of paramere of rhomboid shape but with rounded lateral angles, apex not bilobed, underneath with two groups or rows of ca. 6–8 peg setae along parameral lateral margins (Fig. 13A, C); (in lateral view) median lobe with gradually narrowed and moderately short apex (Fig. B). Known from Russia (from the type locality in Khabarovsk Territory), Middle Asia, and China, but very rare	***Q.koltzei* Eppelsheim, 1887**
17	Infraorbital ridges (head in latero-ventral view) well developed at base only, not reaching base of mandibles. Aedeagus distinctly asymmetrical (Fig. 13D–F). Body pale brown; elytra mostly yellowish with darkened lateral hind angles and, sometimes, also darkened along suture. Body length 6.5–8.0 mm. Widespread in Central Europe, but rather rare. In Russia, from the European part to Northern Caucasus, also recorded from Irkutsk Province. Often associated with ants	***Q.scitus* (Gravenhorst, 1806)**
–	Infraorbital ridges (head in latero-ventral view) well developed throughout their entire length, from neck to base of mandibles. Aedeagus rather symmetrical	**18**
18	Smaller species, body length 6.5–8 mm. Head and pronotum darker, elytra reddish to brownish. Aedeagus as in Fig. 13G–I. Known only from the Caucasus Mountains, in Russia (Northern Caucasus region)	***Q.edmundi* Coiffait, 1969**
–	Larger species, body length 7.5–12 mm. Aedeagus and habitus different	**19**
19	Pronotum, at both sides, with all setiferous punctures of sublateral group situated before (anterior to), or at most at the same level as, large lateral puncture. Aedeagus: (in parameral view) paramere usually of rhomboid shape with moderately sharp apex (e.g., Fig. 13R, U; except for *Q.brevicornis* with strongly bilobed apex, Fig. 13L); (in lateral view) median lobe either wide with broad apex strongly narrowing near it apex (e.g., Fig. 13K), narrower with very gradually narrowing and sharp apex (e.g., Fig. 13T) or elongate with truncate apex (Fig. 13N, Z)	**20**
–	On at least one side of pronotum, basalmost setiferous puncture of sublateral group situated distinctly behind (posterior to) the level of large lateral puncture. Aedeagus: (in parameral view) paramere usually of trapezoidal shape with broad apex (e.g., Fig. 14I, L); (in lateral view) median lobe moderately wide with broad and rounded apex gradually narrowing from the middle of its apical part (e.g., Fig. 14B, K)	**25**
20	Legs entirely or at least partly dark brown to black. Aedeagus distinctly symmetrical	**21**
–	Legs uniformly pale, yellowish to brown, without darkened, dark brown to black, parts. Either aedeagus asymmetrical or, if aedeagus symmetrical, elytra brownish to red, contrasting in coloration with dark brown head and pronotum	**24**
21	Aedeagus: (in parameral view) paramere with broad and strongly bilobed apex (Fig. 13L). Posterior frontal puncture situated in the middle of distance between posterior margin of eye and nuchal ridge. Head and pronotum dark brown to black, elytra much paler, yellowish to reddish. Aedeagus as in (Fig. 13J–L). Body length 9.0–14.0 mm. Distributed throughout Europe except Iberian Peninsula and especially abundant in northern Europe. In Russia, rather rare and known only from Central Northern European and Northern Caucasus regions	***Q.brevicornis* (Thomson, 1860)**
–	Aedeagus: (in parameral view) paramere with broad, but disrtinctly pointed and entire apex. Posterior frontal puncture usually situated closer to posterior margin of eye than to neck constriction. Elytra of the same or very similar color as head and pronotum, brown to dark brown, only exceptionally paler or reddish	**22**
22	Aedeagus (Fig. 13M–O): (in lateral view) apex of median lobe rather broad, with abrupt notch at base of apical portion (Fig. 13N); underside of paramere with sensory peg setae arranged in two shorter rows widely separated, located near parameral lateral margins (Fig. 13O). Body length 8.0–11.0 mm. Habitus as in Fig. 6C. Transpalearctic, including Iceland and apparently introduced to Greenland, North and South America, and to the Australian region. Widespread in Russia, more common along its middle latitudes, from North-Western and Central Northern European regions in the west to Kamchatka and South Kuril in the east	***Q.mesomelinus* (Marsham, 1802)**
–	Aedeagus: (in lateral view) apex of median lobe narrow and moderately sharp, without abrupt notch at base of apical portion (Fig. 13Q, T); underside of paramere with sensory peg setae arranged in one or two indistinct irregular rows in the middle (Fig. 13R, U)	**23**
23	Aedeagus (Fig. 13P–R): (in lateral view) median lobe with sharply narrowing apex (Fig. 13Q); (in parameral view) paramere of rhomboid shape with distinct lateral angles (Fig. 13P); underside of paramere with sensory peg setae arranged in two long rows situated close to each other (Fig. 13R). Body length 7.5–9.0 mm. Widespread in Europe, especially in the north. In Southern Europe in the mountains and absent in most of the Mediterranean, but recorded from Asia Minor. In Russia, recorded only from the European part to Northern Caucasus	***Q.maurus* (C. Sahlberg, 1830)**
–	Aedeagus (Fig. 13S–U): (in lateral view) median lobe with distinctly sharp and gradually narrowing, elongate apex (Fig. 13T); (in parameral view) paramere of rhomboid shape, but with rounded angles (Fig. 13S); underside of paramere with sensory peg setae arranged in one wide median irregular row (Fig. 13U). Body length 9.0 mm. Known from the type locality in Armenia and from a single, questionable literature record in Volgo-Don region of Russia	***Q.tetrapunctatus* Coiffait, 1977**
24	Temples distinctly longer than length of eye, more or less parallel-sided immediately behind eyes, then forming broadly rounded posterior angles of head. Elytra pale brown to red, distinctly different in coloration from dark brown head and pronotum. Aedeagus symmetrical as in Fig. 13V–X. Body length 8.0–11.0 mm. Widespread in Europe, except its westernmost, northernmost and southernmost parts. In Russia, only in the European part as the record from Krasnoyarsk region needs confirmation	***Q.vexans* Eppelsheim, 1881**
–	Temples not longer than length of eye, gradually converging to neck, posterior angles of head indistinct. Elytra of about same coloration as head and pronotum, entire body except appendages brownish. Aedeagus asymmetrical, with elongate and strongly asymmetrical apical portion of median lobe (Fig. 13Y–AA). Body length 7.0–10.0 mm. Transpalearctic, apparently with disjunct boreo-montane distribution. In Russia, widespread from the European part to Primorsky Territory	***Q.xanthopus* Erichson, 1839**
25	Aedeagus apically on parameral side with two more or less dentate longitudinal carinae (best seen when paramere removed from median lobe)	**26**
–	Aedeagus apically on parameral side with only one median longitudinal carina, forming a small tooth at its base (best seen when paramere removed from median lobe)	**29**
26	Aedeagus: (in parameral view) apical portion of paramere narrow, underneath with sensory peg setae arranged in irregular, variable, but always distinctly longitudinal groups (Fig. 14C, F)	**27**
–	Aedeagus: (in parameral view) apical portion of paramere truncate and broad, underneath with sensory peg setae arranged in irregular, variable, but always distinctly transverse groups (Fig. 14I, L)	**28**
27	Aedeagus (Fig. 14A–C): (in parameral view) median lobe with pointed apex (Fig. 14A); paramere with distinctly rhomboid apical portion (Fig. 14C). Pronotum with only one (basalmost) seta of each sublateral group situated behind (posterior to) level of large lateral seta. Body length 8.0–10.0 mm. West Palearctic, introduced to North America and, apparently, to the Oriental Region. In Russia, known from North Western European to Crimea Republic and Northern Caucasus regions	***Q.cruentus* (Olivier, 1795)**
–	Aedeagus (Fig. 14D–F): (in parameral view) median lobe with very broad and weakly emarginate to truncate apex (Fig. 14D); paramere with relatively narrower apical portion, not rhomboid (Fig. 14F). Pronotum with two setae (two basalmost) of each sublateral group situated behind (posterior to) level of large lateral seta. Body length 8.0–11.0 mm. Widespread in the Palearctic and west Oriental regions. In Russia, recorded only from the European part, east to South West Siberia region	***Q.ochripennis* (Ménétriés, 1832)**
28	Elytra yellowish red to red, without metallic luster, contrasting with dark brown to black coloration of rest of body. Elytra black in very rare cases. Aedeagus (Fig. 14G–I): (in lateral view) apex of paramere not protruding over apex of median lobe (Fig. 14H); underside of paramere with sensory peg setae in more or less irregular, non-linear arrangement (Fig. 14I); (in ventral or dorsal view) median lobe with less truncate apex (Fig. 14G). Body length 7.5–11.0 mm. Widely distributed in the Palearctic and introduced everywhere around the world, cosmopolitan. In Russia, not common and known mainly from the European part, but also recorded from south-western Siberia	***Q.fulgidus* (Fabricius, 1793)**
–	Elytra black as in rest of body, often with bluish metallic lustre. Aedeagus (Fig. 14J–L): (in lateral view) apex of paramere slightly protruding over apex of median lobe (Fig. 14K); underside of paramere with sensory peg setae in more or less linear arrangement (Fig. 14L); (in ventral or dorsal view) median lobe with more truncate, relatively broader apex (Fig. 14J). Body length 9.0–12.0 mm. Widely distributed in Europe, except its northern part. Nidicolous, mostly in mole nests. In Russia, known from a single questionable record	***Q.nigrocaeruleus* Fauvel, 1876**
29	Antennal segments less elongate, fourth segment transverse. Male abdominal sternite VIII entirely or at least in anterior three-fourths black, its posterior margin broadly concave, with extremely long black setae (the longest of them longer than antennomere I). Aedeagus as in Fig. 14M–O: paramere (in dorsal view) broader and usually weakly concave apically, more rarely truncate or weakly convex. Apical margin of female sternite VIII with black setae. Habitus as in Fig. 6D. Body length 8.0–11.0 mm. Presumably widely distributed West Palaearctic species; apparently not nidicolous (Assing, 2019). In Russia, known from southern regions of its European part	***Q.invreae* Gridelli, 1924**
–	Antennal segments more elongate, fourth segment not transverse. Male abdominal sternite VIII with at least the anterior and posterior portions pale (brownish) and only median portion usually blackish-brown, its posterior margin shallowly concave only in the middle, with shorter brown setae (barely half as long as antennomere I). Apical margin of female sternite VIII with brown setae. Aedeagus (Fig. 14P): paramere (in dorsal view) more slender and apically distinctly convex. Body length 8.0–11.0 mm. Presumably north and central European species, not common; nidicolous, especially in mole nests. In Russia more reliably known from the westernmost part of Central North European region, but also reported from easternmost part of Kuznetsk-Altai regions	***Q.puncticollis* (Thomson, 1867)**

### Key to species of the subgenus Raphirus Stephens, 1829

**Table:** 

1	Scutellum punctate, setose, even if sometimes with few setae only	**2**
–	Scutellum impunctate, glabrous	**15**
2	Abdomen: first three visible tergites near base at sides with shallow depressions and patches of denser, variegated setation, where setae are variously directed but not uniformly posteriad. Aedeagus (Fig. 14Q–S): underside of paramere with rows of sensory peg setae not reaching very apex of paramere, i.e. apicalmost pegs located below basalmost pair of apical setae (Fig. 14S). Body dark brown to black, head usually darker, elytra sometimes with thin yellowish apical margin; appendages yellowish. Body length 7.0–7.5 mm. West Palearctic species. In Russia, known only from the northern regions of its European part	***Q.semiaeneus* (Stephens 1832)**
–	Abdomen: first three visible tergites smooth near base, without shallow depressions and with regular, even setation, all setae directed posteriad. Aedeagus different	**3**
3	At least metafemora on inner face more or less darkened. Smaller species with body length 4.3–6.5 mm. Third segment of antennae shorter or as long as second segment. Species of this complex can be reliably identified only by the study of male genitalia	**4**
–	All legs entirely pale, metafemora not darkened on inner face. Larger species with body length 7.0–10.5 mm. Third segment of antennae distinctly longer than second segment	**12**
4	Aedeagus (Fig. 14T–V): (in parameral view) paramere wider than median lobe for most of its length, lateral outline of median lobe hidden under paramere (Fig. 14T). Body piceous to piceous black, with variably brown pronotum and usually dark brown elytra, with yellowish appendages. Body length 6.0–6.5 mm. Holarctic species with circumpolar distribution. In the Palearctic, from northern Europe, including Iceland throughout entire northern Russia, from its European part to Kamchatka peninsula	***Q.fulvicollis* (Stephens, 1832)**
–	Aedeagus: (in parameral view) paramere at least along most of its length narrower than median lobe; lateral outline of median lobe well visible for most of its length (e.g., Fig. 15D, G, P)	**5**
5	Aedeagus: (in lateral view) subapical tooth distinct as such, median lobe apicad of this tooth not resembling an axe blade (e.g., Fig. 15E, H, N)	**6**
–	Aedeagus: (in lateral view) subapical tooth not distinct as such because it forms carina extended to the apex of median lobe which, therefore, resembles an axe blade (Fig. 15Q, T, W)	**10**
6	Aedeagus (Fig. 15A–C): (in parameral view) lateral sides of paramere sharply converging apicad after expansion, distinctly narrower in middle portion (Fig. 15A); (in lateral view) median lobe with subapical tooth close to apex (Fig. 15B). Piceous black, with dark brown pronotum and elytra, appendages pale brown to brown. Body length 5.0–6.0 mm. Widespread in Europe, except the southern part. In Russia only in its European part to Northern Caucasus in the south	***Q.persimilis* Mulsant & Rey, 1876**
–	Aedeagus: (in parameral view) sides of paramere gradually converging apicad, almost not narrowing in the middle portion (e.g., Fig. 15D, G, P); (in lateral view) median lobe with subapical tooth situated far from apex and paramere far from reaching apex of median lobe	**7**
7	Body smaller and more gracile, length 4.0–5.5 mm; elytra shorter, some of the species wingless. Aedeagus: (in lateral view) paramere far from reaching apex of median lobe; median lobe wider, subapical tooth situated far from its apex (Fig. 15E, H). Montane species, known from elevations 1000 m and higher	**8**
–	Body larger and more robust, length 5.0–6.2 mm; elytra longer, usually winged species. Aedeagus: (in lateral view) paramere almost reaching apex of median lobe; median lobe narrower, subapical tooth situated closer to its apex (Fig. 15K, N). Species with diverse bionomics	**9**
8	Aedeagus (Fig. 15D–F): (in lateral view) median lobe wide with strongly curved apical portion (Fig. 15E); (in parameral view) paramere moderately wide, underneath with rows of sensory peg setae converging apicad (Fig. 15D, F). Known only from Russia: from the type locality at Teletskoe Lake in Altai Mountains (Altai Republic) and from Nizhneudinsky District (Irkutsk Province, here recorded for the first time)	***Q.centrasiaticus* Coiffait, 1969**
–	Aedeagus (Fig. 15G–I): (in lateral view) median lobe narrower with less curved apical portion (Fig. 15H); (in parameral view) paramere narrower with rows of sensory peg setae not converging apicad (Fig. 15G, I). Known from Northern Caucasus and Turkey	***Q.omissus* Coiffait, 1977**
9	Aedeagus (Fig. 15J–L): (in parameral view) median lobe moderately wide, with apex pointed; paramere gradually narrowing apicad, slightly narrower in middle part, underneath with rows of peg setae extending parallel or slightly converging apicad (Fig. 15J, L). Piceous black, with dark brown pronotum and elytra, and pale brown to brown appendages. Body length 5.0–6.0 mm. West Palearctic species. In Russia, widespread in the European part; also ambiguously recorded from Irkutsk region	***Q.nitipennis* (Stephens, 1833)**
–	Aedeagus (Fig. 15M–O): (in parameral view) median lobe narrower, with narrow but rounded apex; paramere gradually narrowing anteriad, not narrower in middle part, underneath with rows of peg setae converging apicad (Fig. 15M, O). Piceous to piceous black, pronotum and elytra sometimes more or less brownish. Appendages yellowish-brown (Fig. 6E). Body length 5.0–6.2 mm. Circumpolar species, common in the northern parts of Eurasia and North America. In Russia, widespread in the north, but also can be found in the mountains of the southern regions	***Q.fellmani* (Zetterstedt, 1838)**
10	Aedeagus (Fig. 15P–R): (in lateral view) median lobe gradually narrowing apicad at half of its length, with ventral tooth situated far from its apex (Fig. 15Q); (in parameral view) paramere moderately narrow, not parallel sided, usually narrowing at middle (Fig. 15P). Head distinctly transverse; emargination of sixth male sternite shallow. Body length 4.0–4.9 mm. Russian species distributed throughout Siberia, from Krasnoyarsk in the west to Magadan region in the east	***Q.paraboops* Coiffait, 1975**
–	Aedeagus (Fig. 15S–X): (in lateral view) median lobe gradually narrowing apicad in apical third of its length, subapical tooth situated close to its apex (Fig. 15T, W); (in parameral view) paramere very narrow, almost parallel sided or only slightly narrowing at middle (Fig. 15U, X). Head slightly transverse; emargination of sixth male sternite rather deep	**11**
11	Aedeagus (Fig. 15S–U): (in lateral view) subapical tooth of median lobe situated closer to apex, so that ventro-apical axe-like carina shorter (Fig. 15T). Body length 4.5–5.5 mm. Transpalearctic species. In Russia, known from the European part to Lower Amur region, but becoming rare toward the east	***Q.boops* (Gravenhorst, 1802)**
–	Aedeagus (Fig. 15V–X): (in lateral view) subapical tooth of median lobe situated further from apex, so that ventro-apical axe-like carina longer (Fig. 15W). Body length 5.5–6.5 mm. Widely distributed in Europe but distribution is unclear due to frequent confusion with *Q.boops*. In Russia, known from the European part to Zabaikalsky Territory region in the east, more rare in eastern regions	***Q.boopoides* Munster, 1923**
12	Aedeagus (Fig. 16A–C): (in lateral view) median lobe with subapical tooth, (in dorsal view) with broad apical portion. On average larger, length of body 8.0–10.5 mm. Piceous black, often with brown elytra and apical margins of abdominal tergites; pronotum sometimes brown or reddish-brown; appendages yellowish-brown. West Palearctic species. In Russia, known only from Eastern and Northern Caucasus	***Q.semiobscurus* (Marsham, 1802)**
–	Aedeagus (in lateral view): median lobe without subapical tooth, (in dorsal view) with narrow apical portion (Fig. 16D). On average smaller, length of body 7.0–8.5 mm	**13**
13	Aedeagus (Fig. 16D–F): (parameral view) paramere relatively shorter, its apex very far from reaching apex of median lobe (Fig. 16D); narrow apical portion of median lobe elongate and (in lateral view) slightly curved dorsad (Fig. 16E). Piceous black, sometimes with dark-brown pronotum, elytra and apical margins of abdominal tergites; appendages brown (Fig. 6F). Body length 7.0–8.5 mm. In Russia, widely distributed in Northern Caucasus, also known in the mountains of Turkey	***Q.korgeanus* Fagel, 1968**
–	Aedeagus: (in parameral view) paramere relatively longer, its apex reaching closer to apex of median lobe; narrow apical portion of median lobe short and (in lateral view) slightly acute	**14**
14	Aedeagus (Fig. 16G, H): (in parameral view) median lobe and paramere broader, underside of paramere with numerous sensory peg setae, covering entire third of its length; (in lateral view) apex of paramere almost reaching apex of median lobe. Head black, pronotum piceous with rufotestaceous lateral margins, elytra rufobrunneous to rufotestaceous, abdomen predominantly piceous-black to black. Body length 7.5–8.0 mm. Known only from the Russian Far East	***Q.ryvkini* Smetana, 2018**
–	Aedeagus (Fig. 16I, J): (in parameral view) median lobe and paramere narrower, underside of paramere with sensory peg setae less numerous, forming narrow median field in its apical portion; (in lateral view) apex of paramere not reaching apex of median lobe. Head black, pronotum uniformly piceous, elytra piceous, abdomen piceous-black to black with apical margins of tergites more or less narrowly paler. Body length 7.5 mm. Known only from the Russian Far East	***Q.aedilis* Smetana, 2018**
15	Frons with pair of setiferous punctures between anterior frontal punctures	**16**
–	Frons without setiferous punctures between anterior frontal punctures	**17**
16	Aedeagus (Fig. 16K–M): (in lateral view) median lobe with apex distinctly curved ventrad (Fig. 16L); (in parameral view) paramere with rows of sensory peg setae converging basad (Fig. 16K, M). Abdominal tergites brown, usually with darker longitudinal median and lateral spots. Habitus as in Fig. 7A. Body length 5.0–6.0 mm. West Palaearctic species. In Russia, known only from two regions (North-western and Central North) of its European part	***Q.lucidulus* Erichson, 1839**
–	Aedeagus (Fig. 16N–P): (in lateral view) median lobe with straight apex (Fig. 16O); (in parameral view) paramere with rows of sensory peg setae extended along parameral margins, slightly diverging from sides basad (Fig. 16N, P). Abdominal tergites brown to dark brown, but never with distinct color pattern of longitudinal spots. Body length 5.0–6.0 mm. Distributed in the West Palearctic. In Russia, recorded from the European part southwards to Northern Caucasus	***Q.scintillans* (Gravenhorst, 1806)**
17	Head (in dorsal or lateral view): eyes about 3–4 times as long as temples, so large that they occupy almost entire lateral side of head before neck constriction, leaving only very short temples. Body and appendages pale: head and elytra testaceous brown; pronotum testaceous brown with paler, yellowish margins. Aedeagus as in Fig. 16Q–S. Body length 5.5–6.5 mm. Montane species. In Europe, recorded from eastern Alps, Carpathians and mountains of north-western Balkans. In Russia, known from a few questionable literature records from Kuznetsky Altai and North Eastern European regions	[***Q.cincticollis* Kraatz, 1857**]
–	Head (in dorsal or lateral view): eyes about 1.5–2.5 times as long as temples, never as large as to occupy almost entire lateral side of head before neck constriction. Habitus and aedeagus not as in *Q.cincticollis* below	**18**
18	Head with two basal punctures on each side forming oblique row with posterior frontal puncture. Neck relatively narrow; pronotum widest shortly before its middle; elytra relatively long; abdominal tergites at sides with flecks of denser and longer variegated golden setae. Coloration of the whole body piceous black (Fig. 7B). Aedeagus as in Fig. 16T–V. Body length 6.0–7.0 mm. Widely distributed in Central Europe except northern part. In Russia, recorded only from the Northern Caucasus region. Inhabits wet debris near stream edges (ripicolous species)	***Q.riparius* F. Kellner, 1843**
–	Head with one basal puncture on each side; (if temples densely punctuate, basal punctures are recognized as significantly larger and located medialmost), with two basal punctures only exceptionally (possibly on one side only), but never forming oblique row with posterior frontal puncture. Habitus and aedeagus not as in *Q.riparius*	**19**
19	Surface of elytra between setiferous punctures (interspaces) very glossy, without distinct, more or less reticulate microsulpture, at most with some very faint irregularities (viewed at high magnification)	**20**
–	Surface of elytra between setiferous punctures (interspaces) rather dull, with distinct, more or less reticulate microsculpture (viewed at high magnification)	**35**
20	Posterior frontal punctures, each, with one to three or even four smaller additional punctures nearby. Relatively large, dark brown to black species with reddish elytra and pale, yellowish-brown legs. Aedeagus as in Fig. 16W–Y. Body length 8.0–11.0 mm. Widespread in Europe and extending to Asia Minor. In Russia known only from South-Western Siberian region based on a single ambiguous record	[***Q.picipes* (Mannerheim, 1830)**]
–	Posterior frontal punctures, each, without one or more smaller additional punctures nearby. Habitus and structure of aedeagus different	**21**
21	Labrum entire (at most with slight apical notch medially); abdomen parallel-sided along most of its length, not distinctly tapering apicad. Aedeagus (Fig. 17A–C): internal sac with pair of large sclerites (Fig. 8C), median lobe (in lateral view) with sharp and curved hook-like apex (Fig. 17B); (in parameral view) paramere broad and plate-like (Fig. 17A, C). Dark brown to brownish, with pronotum and appendages usually paler (Fig. 7C). Body length 5.8–7 mm. Known only from Russia: from the northern regions of its European part and throughout entire Siberia, except Far East	***Q.jenisseensis* Sahlberg, 1880**
–	Labrum bilobed; abdomen distinctly tapering apicad. Aedeagus: internal sac without large, conspicuous sclerites, median lobe (in lateral view) without curved, hook-like apex (e.g., Fig. 17K, N)	**22**
22	Elytra longer than, or as long as pronotum, longer than wide (Fig. 7D), sometimes with paler sides. Smaller, body length 5.0–8.5 mm. Mostly widespread species (only *Q.gemellus* and *Q.vulneratus* confined to Caucasus and Asia Minor)	**23**
–	Elytra shorter than pronotum; wider than long, never bicolored (Figs 7F, 8A, B). Larger, body length 8–12 mm. All species confined to the Caucasus and Asia Minor	**32**
23	Pronotum pale brown or reddish, contrasting with dark brown to black head. Aedeagus (Fig. 17D–F): (in parameral view) paramere in apical two thirds of its length narrower than corresponding part of median lobe, so that lateral margins of median lobe visible from under paramere; apical portion of median lobe first expanding and then narrowing towards apex, lanceolate (Fig. 17D); paramere underneath with sensory peg setae forming loose and relatively long rows, with distances between pegs mostly much wider than peg diameter (Fig. 17F). Body length 7.5–8.5 mm. Widespread in Europe, but more abundant in its western part, becoming rare towards the east. In Russia, known only from a few literature records from the European part and Irkutsk Province	***Q.nigriceps* Kraatz, 1857**
–	Coloration of body and/or structure of aedeagus different	**24**
24	Aedeagus: (in lateral view) median lobe straight, never curved (for example as in Fig. 17Q, T); (in parameral view) paramere thin, parallel sided or only slightly narrowing at middle, underneath with two distinct regular rows of setae along each parameral lateral margin (e.g., Fig. 17 L, O, R)	**25**
–	Aedeagus: (in lateral view) median lobe slightly or distinctly curved (e.g., Fig. 17W, Z); (in parameral view) paramere wide and not parallel sided, usually narrowing at middle, underneath with two irregular rows of peg setae along each parameral lateral margins (Fig. 17X, AA)	**29**
25	Aedeagus: (in lateral view) median lobe with sharp apex (e.g., Fig. 17K); (in parameral view) paramere underneath with two short rows of peg setae only slightly extending basad of lateral setae (for example as in Fig. 17I)	**26**
–	Aedeagus: (in lateral view) median lobe with broad apex (for example as in Fig. 17Z); (in parameral view) paramere underneath with two longer rows of peg setae extending far basad of lateral setae (for example as in Fig. 17R, U)	**27**
26	Aedeagus (Fig. 17G–I): (in parameral view) paramere with narrower apex, underneath with 3–5 peg setae in each of two rows (Fig. 17G); (in lateral view) apical portion of median lobe moderately narrow (Fig. 17H). Body length 5.0–6.0 mm. Widespread in the West and Central Palearctic, with the eastern extent of distribution in Russia, extending through Krasnoyarsk, Irkutsk, Buryatia, and Zabaikalsky regions	***Q.limbatus* (Heer, 1839)**
–	Aedeagus (Fig. 17J–L): (in parameral view) paramere with broadly rounded apex, underneath with more than 10 peg setae in each of two rows (Fig. 17J); (in lateral view) apical portion of median lobe very narrow (Fig. 17K). Body length 6.5–7.5 mm. Relatively common in Europe except northern part, reaching Asia Minor. In Russia, known only from the Caucasus	***Q.suturalis* Kiesenwetter, 1845**
27	Elytra distinctly longer than wide and distinctly longer than pronotum. Aedeagus (Fig. 17M–O): (in parameral view) apex of median lobe pointed; underside of paramere with sensory peg setae forming dense and short row (Fig. 17O); (in lateral view) median lobe with subapical tooth situated far from its apex (Fig. 17N). Body length 6.0–7.5 mm. West Palearctic species. In Russia, known only from scattered and dubious literature records from Middle Volga, Northern Caucasus, Krasnoyarsk, Kuznetsky Altai, Buryatia, and Irkutsk regions	[***Q.humeralis* Stephens, 1832**]
–	Elytra as long as and slightly longer than pronotum (Fig. 7D). Aedeagus: (in parameral view) apex of median lobe not pointed, underside of paramere with sensory peg setae forming thin and long rows (e.g., Fig. 17R); (in lateral view) median lobe with ventral tooth situated close to its apex (e.g., Fig. 17Q)	**28**
28	Elytral width greater or subequal to length; posterior margin of tergite VII sometimes with palisade fringe (Fig. 7D). Aedeagus (Fig. 17P–R): (in lateral view) median lobe with subapical tooth situated further from its apex, apex of paramere reaching or almost reaching apex of median lobe (Fig. 17Q). Body length 4.5–6.5 mm. Circumpolar species, common in northern territories of North America, rarer in Asia. In Russia, known mainly from the northern regions but can also be found in the mountains of the southern regions	***Q.sublimbatus* Mäklin, 1853**
–	Elytral width distinctly greater than length, posterior margin of tergite VII without palisade fringe. Aedeagus (Fig. 17S–U): (in lateral view) median lobe with subapical tooth situated close to its apex, apex of paramere extending distinctly beyond apex of median lobe (Fig. 17T). Body length 5.0–6.5 mm. Common, endemic to the north-western Caucasus	***Q.gemellus* Eppelsheim, 1889**
29	Elytra unicolored, never with paler apical margins. Aedeagus (Fig. 17V–X): (in lateral view) median lobe moderately sharp with apical portion distinctly curved dorsad, subapical tooth situated close to its apex (Fig. 17W). Body length 6.0–7.5 mm. Common in West Palearctic and reaching Middle Asia. Rather common in Russia, with eastern border of distribution extending through North Yenissei, Krasnoyarsk and Irkutsk regions	***Q.umbrinus* Erichson, 1839** [poorly known *Q.angaricus* may fit here too, see the Annotated catalogue section for details]
–	Elytra not unicolored, with slightly or distinctly paler apical margins (Fig. 7E). Aedeagus different	**30**
30	Aedeagus (Fig. 17Y–AA): (in parameral view) apex of paramere acuminate, with ca. 7 peg setae in each of two rows, only slightly extending basad of lateral setae; (in lateral view) ventral tooth situated nearly at apex of median lobe. Body length 6.0–7.0 mm. Widely distributed in Europe. In Russia, known only from a few ambiguous records from Irkutsk province and Kuznetsk Altai	[***Q.maurorufus* (Gravenhorst, 1806)**]
–	Aedeagus: (in parameral view) apex of paramere evenly converging anteriad, with ca. 15 peg setae in each of two rows, extending far basad of lateral setae; (in lateral view) ventral tooth situated nearly at the apex of median lobe	**31**
31	Smaller species, body length 6.0–7.5 mm. Aedeagus (Fig. 17BB–DD): (in lateral view) median lobe with more rounded apex (Fig. 17CC); (in parameral view) paramere underneath with two rows of peg setae very close to parameral lateral margins, divergent (Fig. 17DD). Widespread in Europe and known from Asia Minor. In Russia known from several literature records in its European part	[***Q.nemoralis* Baudi de Selve, 1848**]
–	Larger species (Fig. 7E), body length 8.0–8.5 mm. Aedeagus (Fig. 18A–C): (in lateral view) median lobe with sharper apex (Fig. 18B); (in parameral view) paramere underneath with two rows of peg setae closer to parameral midline than to lateral margins, convergent (Fig. 18C). Known only from the Caucasus and Turkey. In Russia in the Northern and Eastern Caucasus regions	***Q.vulneratus* Gemminger and Harold, 1868**
32	Body blackish, appendages and posterior margins of abdominal tergites brownish. Aedeagus: (in lateral view) median lobe with thin and curved apical portion (Fig. 18E, H); (in parameral view) apex of paramere rounded, underneath with regular rows of peg setae (Fig. 18F, I)	**33**
–	Body from dark reddish brown to yellowish brown, appendages and posterior margins of abdominal tergites usually lighter (Figs 7F, 8A, B). Aedeagus: (in lateral view) median lobe with wide and only slightly curved apical portion (Fig. 18K, N); (in parameral view) apex of paramere more elongate, underneath with irregular groups of peg setae (Fig. 18L, O)	**34**
33	Pronotum gradually narrowing anteriad; elytra moderately short (Fig. 7F). Aedeagus (Fig. 18 D–F): (in lateral view) median lobe with thin and strongly curved apical portion (Fig. 18E); (in parameral view) paramere with narrow and acuminate apex (Fig. 18D). Body length 9.5–12 mm. Endemic to the north-western Caucasus and found at high altitudes in subalpine and alpine zones (2000–2700 m)	***Q.lgockii* Roubal, 1911**
–	Pronotum more strongly narrowing anteriad; elytra very short. Aedeagus (Fig. 18G–I): (in lateral view) median lobe with broader and less curved apical portion (Fig. 18H); (in parameral view) paramere with relatively broader apex, not lanceolate (Fig. 18G). Body length 9.5 mm. The species is known only from the holotype from the unspecified locality “Caucasus”, so its presence in Russia is uncertain	[***Q.brachypterus* Coiffait, 1967**]
34	Aedeagus (Fig. 18J–L): (in lateral view) median lobe with moderately sharp apex and subapical tooth situated closer to its apex; apex of paramere almost reaching apex of median lobe (Fig. 18K); (in parameral view) paramere underneath with ca. 40–50 peg setae arranged in two longitudinal groups (Fig. 18L). Body length 8.0–10.5 mm. Habitus as in Fig. 8A. Endemic to the north-western Caucasus where it can be found from lower altitudes to the timber line	***Q.obliqueseriatus* Eppelsheim, 1889**
–	Aedeagus (Fig. 18M–O): (in lateral view) median lobe with subapical tooth situated further from its broader apex (Fig. 18N); (in parameral view) paramere relatively short, far from reaching apex of median lobe, underneath with ca. 30 sensory peg setae arranged in one irregular group (Fig. 18O). Body length 8.6–9.7 mm. Described from Georgia, here recorded from Russia (Krasnodar Territory) for the first time	***Q.humosus* Solodovnikov, 2005**
35	Smaller species with body length 7.0–9.0 mm. Body brown, elytra with only slightly lighter margins. Aedeagus (Fig. 18P–R): (in parameral view) apex of paramere nearly reaching apex of median lobe, paramere underneath with long and thin scattered groups of peg setae (Fig. 18P, R). Distributed in Europe and North Africa. In Russia recorded only from the European part south to North Caucasus	***Q.fumatus* (Stephens, 1833)**
–	Larger species with body length 8.0–11.0 mm. Body brown to blackish; elytra from completely dark to completely pale but most commonly pale with more or less darkened margins (Fig. 8B). Aedeagus (Fig. 18S–U): (in parameral view) apex of paramere reaching or nearly reaching apex of median lobe, paramere underneath with short and broad groups of densely spaced peg setae (Fig. 18S, U). Widespread in the Caucasus and Asia Minor. In Russia common in the Northern Caucasus region	***Q.suramensis* Eppelsheim, 1880** 3

### Key to species of the subgenus Velleius Leach, 1819

**Table:** 

1	Large and robust beetles, 15.0–24.0 mm. Entire body black, sometimes elytra dark brown. Pronotum distinctly transverse, laterally explanate, distinctly wider than head. Aedeagus as in Fig. 18Y–AA. Transpalearctic, distributed from Europe to Japan. In Russia widely distributed, found from the European to Far East regions, but not common. Associated with nests of *Vespacrabro*	***Q.dilatatus* Fabricius, 1787**

**Figure 5.**
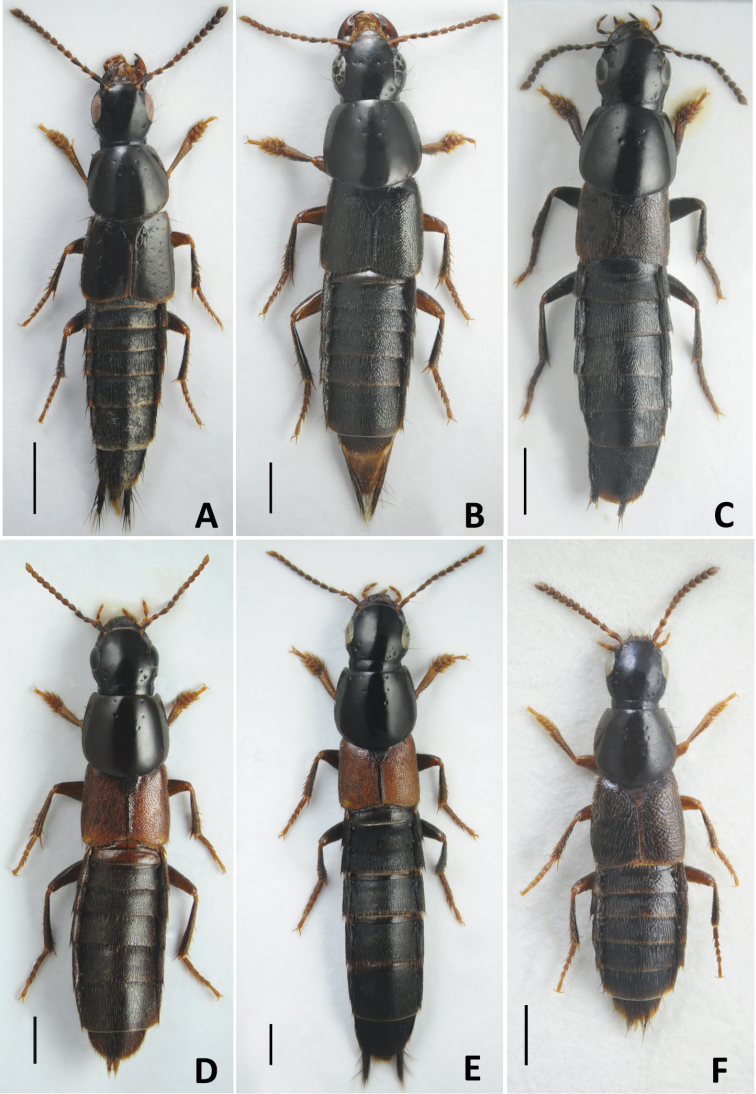
Habitus of *Quedius* recorded from Russia. **A***Q.minor***B***Q.levicollis***C***Q.sundukovi***D***Q.balticus***E***Q.molochinus***F***Q.tenellus*. All scale bars 1 mm.

**Figure 6.**
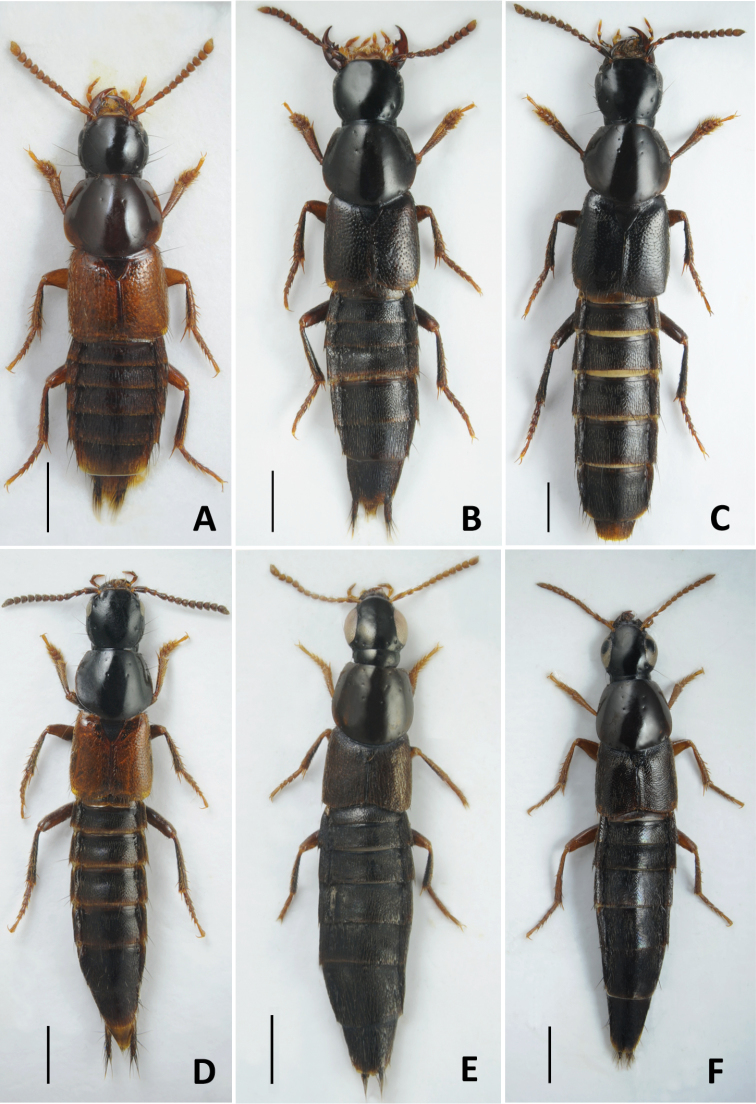
Habitus of *Quedius* recorded from Russia. **A***Q.brevis***B***Q.fasciculatus***C***Q.mesomelinus***D***Q.invreae***E***Q.fellmani***F***Q.korgeanus*. All scale bars 1 mm.

**Figure 7.**
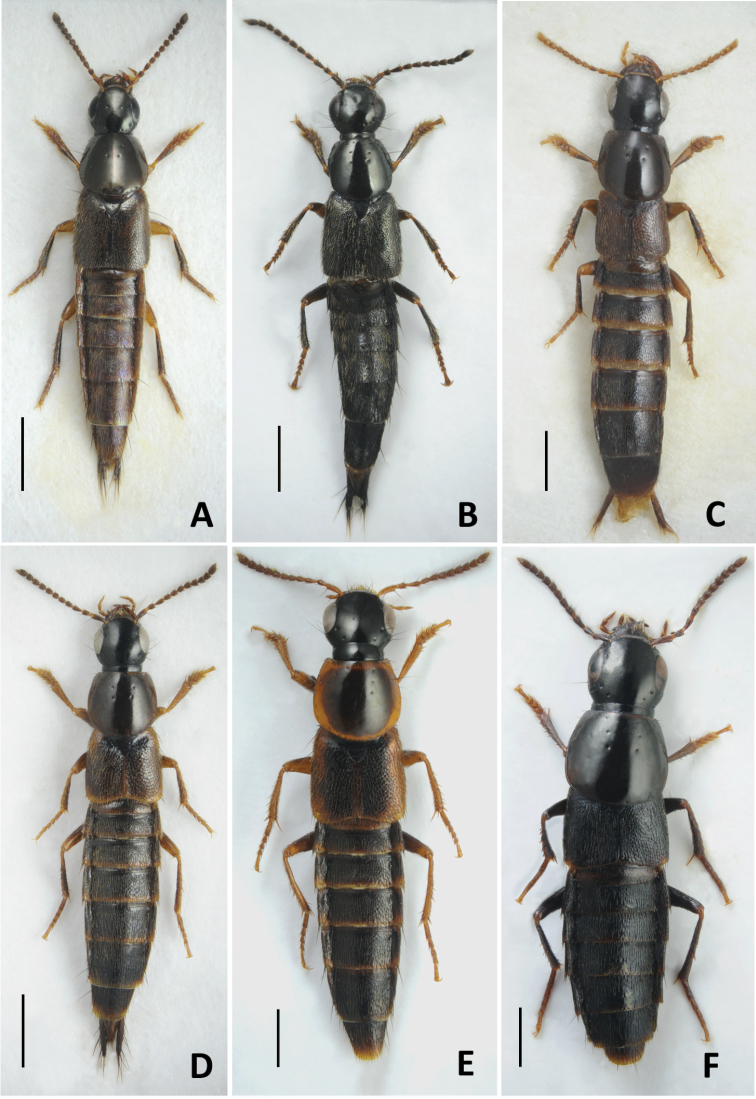
Habitus of *Quedius* recorded from Russia. **A***Q.lucidulus***B***Q.riparius***C***Q.jenisseensis***D***Q.sublimbatus***E***Q.vulneratus***F***Q.lgockii*. All scale bars 1 mm.

**Figure 8.**
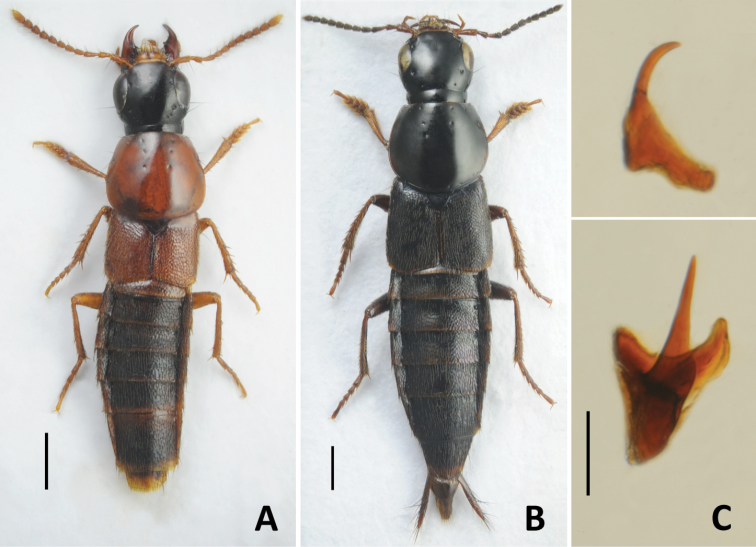
Habitus of *Quedius* recorded from Russia. **A***Q.obliqueseriatus***B***Q.suramensis*. Sclerites in the internal sac of *Q.jenisseensis* (**C**). Scale bars: 1 mm (**A, B**); 0.5 mm (**C**).

## Annotated catalogue of species of *Quedius* of Russia

This annotated catalogue provides details about identity, general distribution, and bionomics of every species. Complete synonymies for each species can be found in the catalogue of Herman (2001). Here we list only synonyms proposed later and not accounted in that world catalogue. In brief format all synonyms published before 2015 can be also found in the Palaearctic Catalogue (Schülke and Smetana 2015).

Species distributions within Russia are given in the form of abbreviated regions from which a given species was recorded with reference to the respective literature or collection source. For easier navigation, abbreviations of the regions are listed alphabetically for each species. In cases where it was necessary but impossible to establish exact localities for species records based on old references, we simply cited these papers, with the original data given verbatim, where available. One catalogue to which we also refer here (Silfverberg 1992) provided species distributions as a summary list of larger territories, which do not coinside with the regions we use here. Regions in Silfverberg (1992), namely Karelia Republic, Murmansk province, left banks of Onega and Kena rivers in Arkhangelsk province, northern part of Andomian upland, and right banks of Svir and Neva rivers in Leningrad province, are here referred altogether as ‘northern part of European Russia’.

Species whose presence in the Russian fauna is strongly ambiguous are given in square brackets, i.e., in the same way as in the keys above. Species whose taxonomic identity is ambiguous and need a revision are marked with an asterisk *.

**Figure 9.**
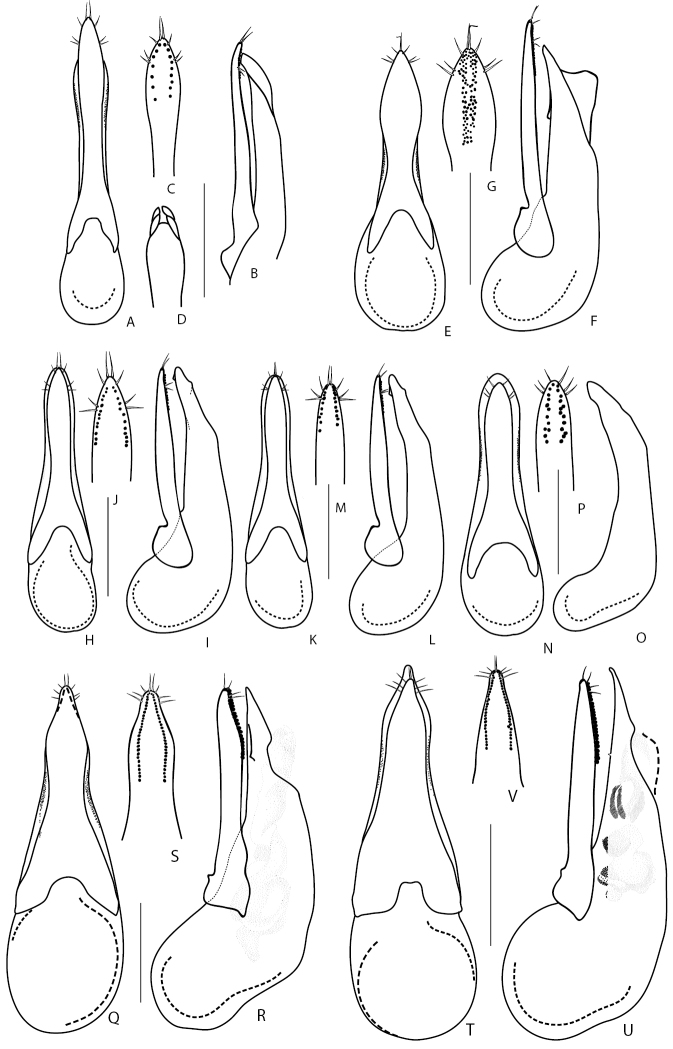
Aedeagi of *Quedius* recorded from Russia: parameral view (**A, E, H, K, N, Q, T**), lateral view (**B, F, I, L, O, R**), underside of paramere (**C, G, J, M, P, S, V**), median lobe in ventral view (**D**). *Q.japonicus* (modified from Smetana 1998) (**A–D**); *Q.fusus* (**E–G**); *Q.cinctus* (**H–J**); *Q.minor* (**K–M**); *Q.kamchaticus* (modified from Smetana 1976) (**N–P**); *Q.fuliginosus* (**Q–S**); *Q.curtipennis* (**T–V**). Scale bars: 1 mm (**Q, R, T, U**), 0.8 mm (**S, V**), 0.5 mm (**A, B, E, F, H, I, K, L, N, O**), 0.25 mm (**C, D, G, J, M, P**).

**Figure 10.**
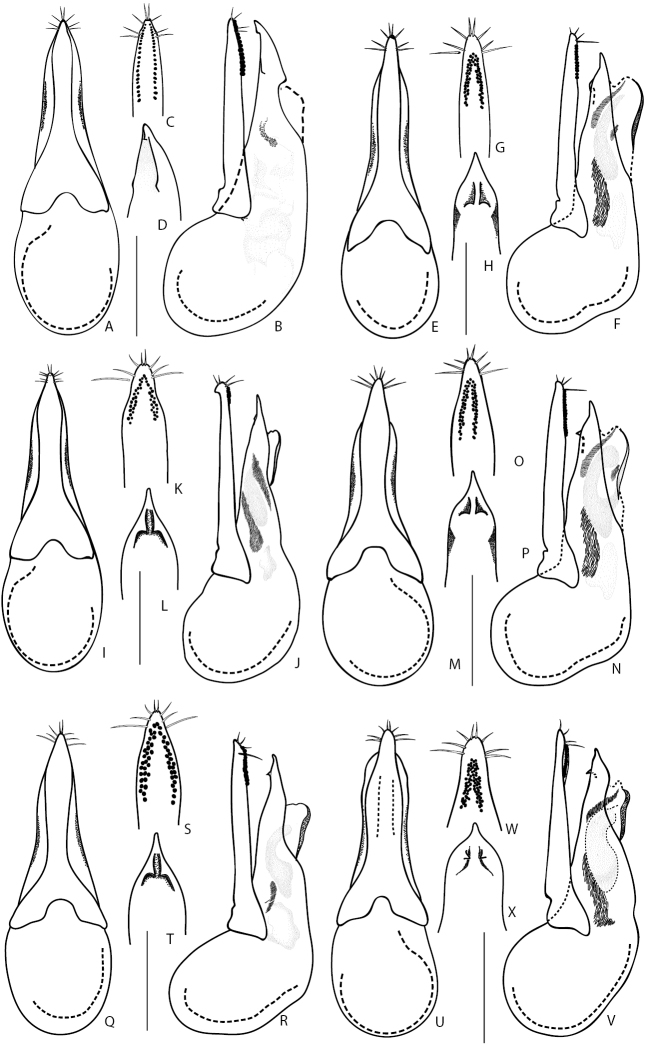
Aedeagi of *Quedius* recorded from Russia: parameral view (**A, E, I, M, Q, U**), lateral view (**B, F, J, N, R, V**), underside of paramere (**C, G, K, O, S, W**), median lobe in ventral view (**D, H, L, P, T, X**). *Q.levicollis* (**A–D**); *Q.sundukovi* (**E–H**); *Q.vicinus* (**I–L**); *Q.molochinus* (**M–P**); *Q.meridiocarpathicus* (**Q–T**); *Q.balticus* (**U–X**). Scale bars: 1 mm (**A, B, E, F, I, J, M, N, Q, R, U, V**), 0.8 mm (**C, D, G, H, K, L, O, P, S, T, W, X**).

**Figure 11.**
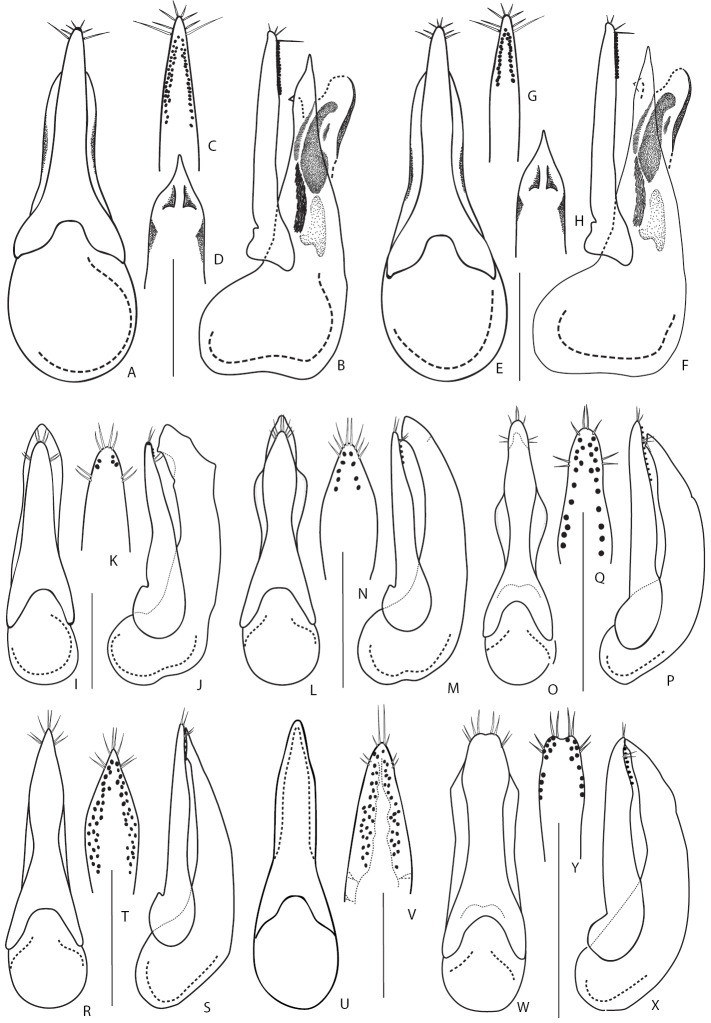
Aedeagi of *Quedius* recorded from Russia: parameral view (**A, E, I, L, O, R, U, W**), lateral view (**B, F, J, M, P, S, X**), underside of paramere (**C, G, K, N, Q, T, V, Y**), median lobe in ventral view (**D, H**). *Q.subunicolor* (**A–D**); *Q.altaicus* (**E–H**); *Q.truncicola* (**I–K**); *Q.microps* (**L–N**); *Q.infuscatus* (**O–Q**); *Q.lundbergi* (**R–T**); *Q.amurensis* (modified from Smetana and Shavrin 2018) (**U, V**); *Q.sofiri* (**W–Y**). Scale bars: 1 mm (**A, B, E, F**), 0.8 mm (**C, D, G, H**), 0.5 mm (**I, J, L, M, O, P, R, S, U, W, X**), 0.25 mm (**K, N, Q, T, V, Y**).

**Figure 12.**
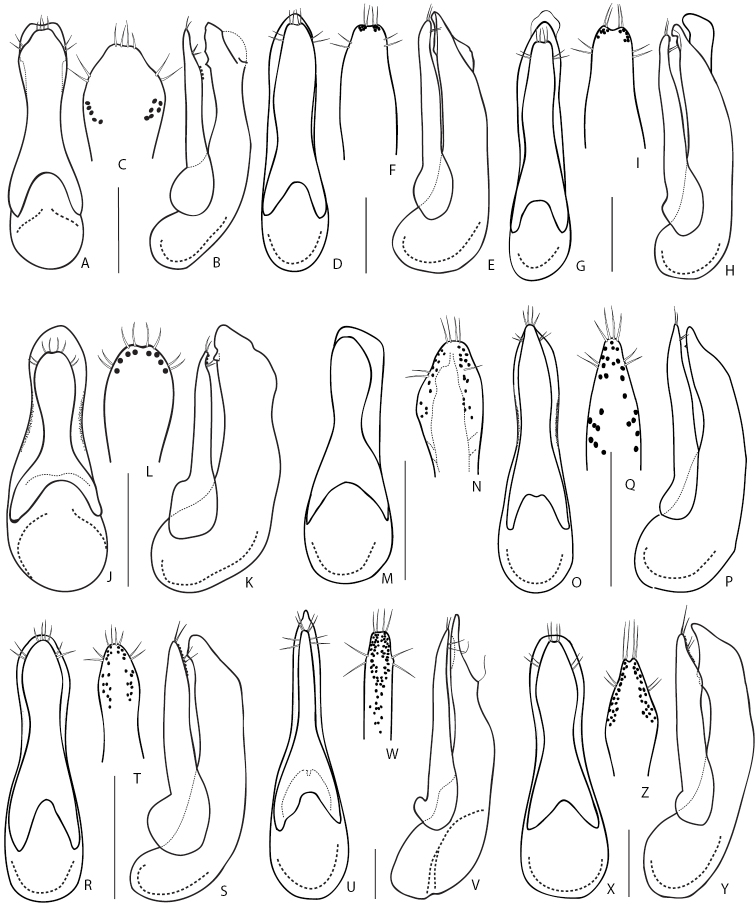
Aedeagi of *Quedius* recorded from Russia: parameral view (**A, D, G, J, M, O, R, U, X**), lateral view (**B, E, H, K, P, S, V, Y**), underside of paramere (**C, F, I, L, N, Q, T, W, Z**). *Q.longicornis* (**A–C**); *Q.roma* (**D–F**); *Q.repentinus* (**G–I**); *Q.tenellus* (**J–L**); *Q.conviva* (modified from Smetana and Shavrin 2018) (**M, N**); *Q.brevis* (**O–Q**); *Q.citelli* (**R–T**); *Q.abdominalis* (**U–W**); *Q.fasciculatus* (**X–Z**). Scale bars: 0.5 mm (**A, B, D, E, G, H, J, K, M, O, P, R, S, U, V, X, Y**), 0.25 mm (**C, F, I, L, N, Q, T, W, Z**).

**Figure 13.**
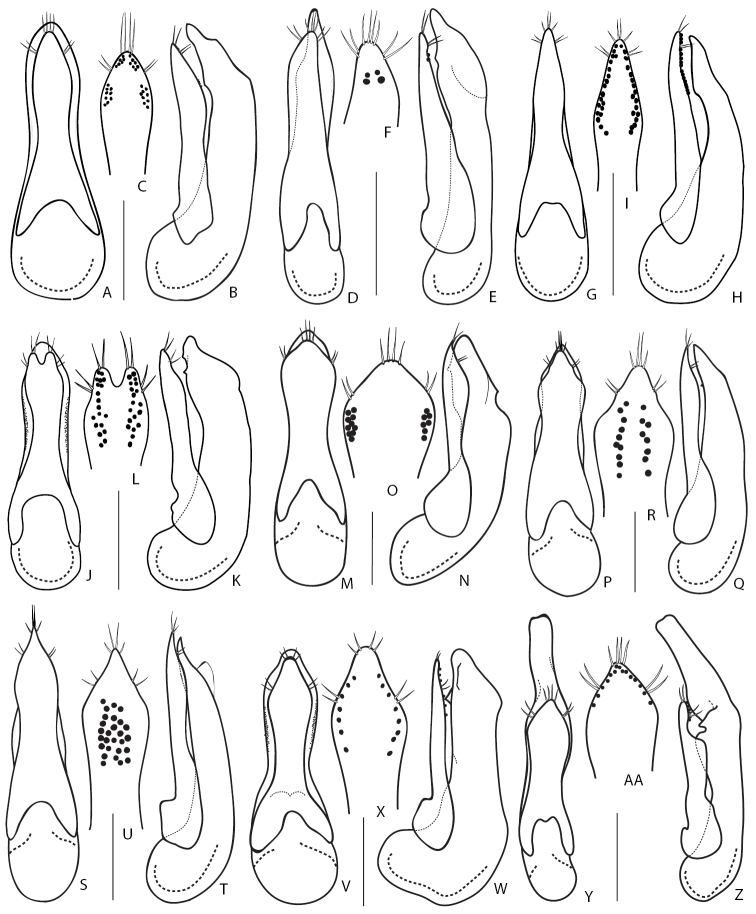
Aedeagi of *Quedius* recorded from Russia: parameral view (**A, D, G, J, M, P, S, V, Y**), lateral view (**B, E, H, K, N, Q, T, W, Z**), underside of paramere (**C, F, I, L, O, R, U, X, AA**). *Q.koltzei* (modified from Coiffait 1978) (**A–C**); *Q.scitus* (**D–F**); *Q.edmundi* (**G–I**); *Q.brevicornis* (**J–L**); *Q.mesomelinus* (**M–O**); *Q.maurus* (**P–R**); *Q.tetrapunctatus* (modified from Coiffait 1969) (**S–U**); *Q.vexans* (**V–X**); *Q.xanthopus* (**Y–AA**). Scale bars: 0.5 mm (**A, B, D, E, G, H, J, K, M, N, P, Q, S, T, V, W, V, Y, Z**), 0.25 mm (**C, F, I, L, O, R, U, X, AA**).

**Figure 14.**
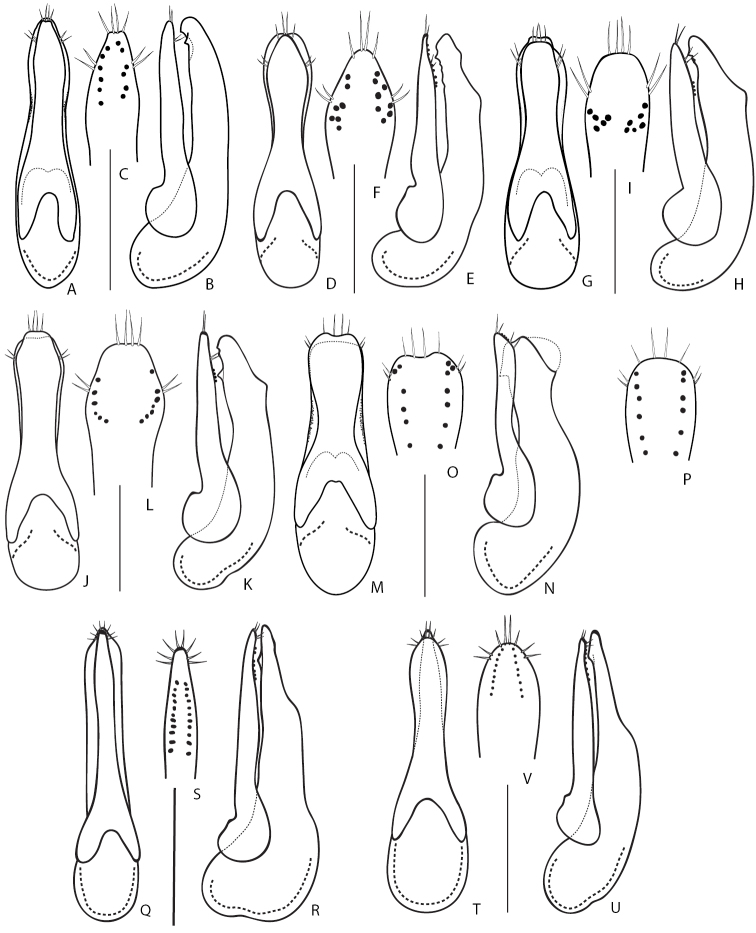
Aedeagi of *Quedius* recorded from Russia: parameral view (**A, D, G, J, M, Q, T**), lateral view (**B, E, H, K, N, R, U**), underside of paramere (**C, F, I, L, O, P, S, V**). *Q.cruentus* (**A–C**); *Q.ochripennis* (**D–F**); *Q.fulgidus* (**G–I**); *Q.nigrocaeruleus* (**J–L**); *Q.invreae* (**M–O**); *Q.puncticollis* (**P**); *Q.semiaeneus* (**Q–S**); *Q.fulvicollis* (**T–V**). Scale bars 0.5 mm (**A, B, D, E, G, H, J, K, M, N, P, Q, R, T, U**), 0.25 mm (**C, F, I, L, O, S, V**).

**Figure 15.**
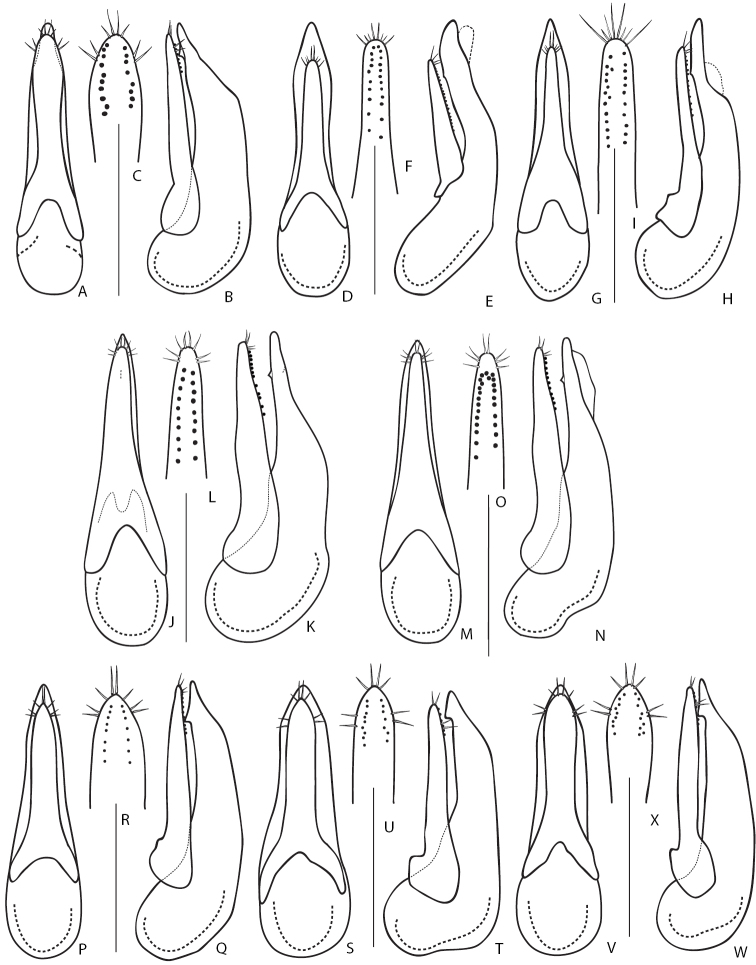
Aedeagi of *Quedius* recorded from Russia: parameral (**A, D, G, J, M, P, S, V, Y**), lateral view (**B, E, H, K, N, Q, T, W, Z**), underside of paramere (**C, F, I, L, O, R, U, X, AA**). *Q.persimilis* (**A–C**); *Q.centrasiaticus* (**D–F**); *Q.omissus* (modified from Coiffait 1977) (**G–I**); *Q.nitipennis* (**J–L**); *Q.fellmani* (**M–O**); *Q.paraboops* (**P–R**); *Q.boops* (**S–U**); *Q.boopoides* (**V–X**). Scale bars: 0.5 mm (**A, B, D, E, G, H, J, K, M, N, P, Q, S, T, V, W, V**), 0.25 mm (**C, F, I, L, O, R, U, X**).

**Figure 16.**
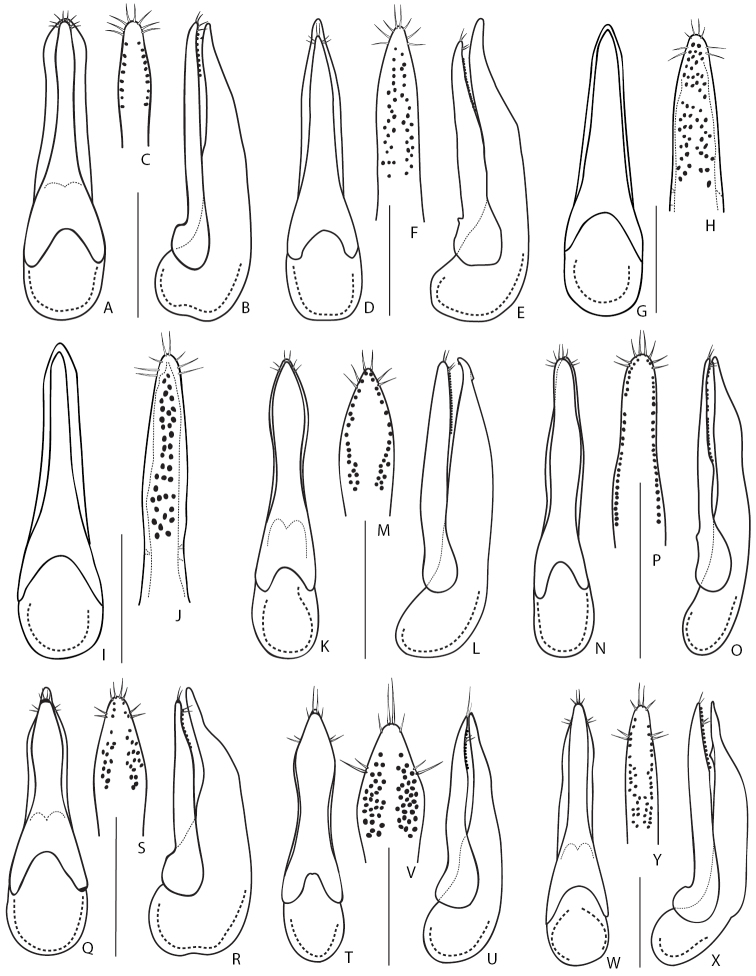
Aedeagi of *Quedius* recorded from Russia: parameral view (**A, D, G, I, K, N. Q, T, W**), lateral view (**B, E, L, O, R, U, X**), underside of paramere (**C, F, H, J, M, P, S, V, Y**). *Q.semiobscurus* (**A–C**); *Q.korgeanus* (**D–F**); *Q.ryvkini* (modified from Smetana and Shavrin 2018) (**G, H**); *Q.aedilis* (modified from Smetana and Shavrin 2018) (**I, J**); *Q.scintillans* (**K–M**); *Q.lucidulus* (**N–P**); *Q.cincticollis* (**Q–S**); *Q.riparius* (**T–V**); *Q.picipes* (**W–Y**). Scale bars 0.5 mm (**A, B, D, E, G, I, K, L, N, O, Q, R, T, U, W, X**), 0.25 mm (**C, F, H, J, M, P, S, V, Y**).

**Figure 17.**
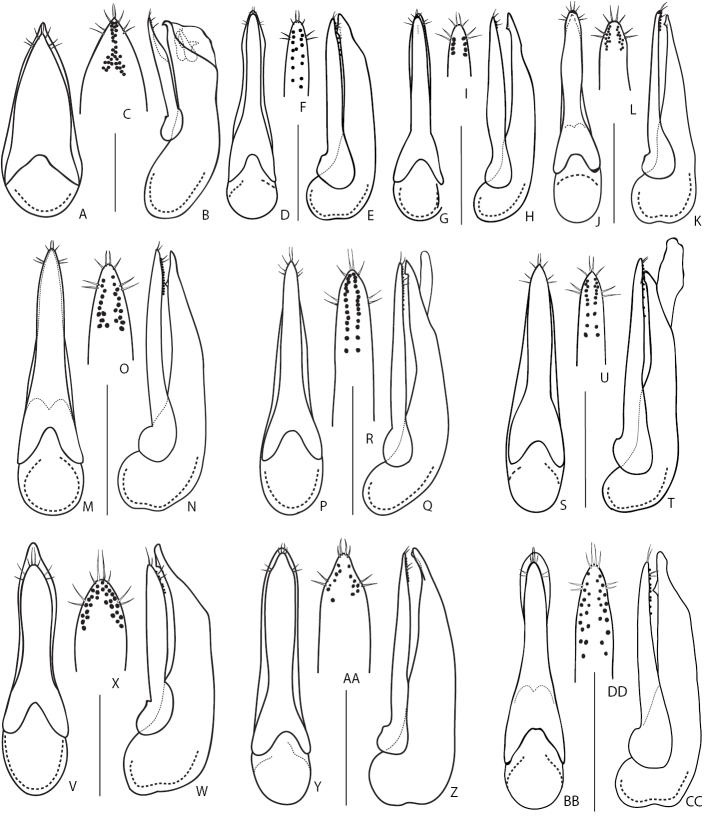
Aedeagi of *Quedius* recorded from Russia: parameral (**A, D, G, J, M, P, S, V, Y**), lateral view (**B, E, H, K, N, Q, T, W, Z**), underside of paramere (**C, F, I, L, O, R, U, X, AA**). *Q.jenisseensis* (**A–C**); *Q.nigriceps* (**D–F**); *Q.limbatus* (**G–I**); *Q.suturalis* (**J–L**); *Q.humeralis* (**M–O**); *Q.sublimbatus* (**P–R**); *Q.gemellus* (**S–U**); *Q.umbrinus* (**V–X**); *Q.maurorufus* (**Y–AA**); *Q.nemoralis* (**BB–DD**). Scale bars: 0.5 mm (**A, B, D, E, G, H, J, K, M, N, P, Q, S, T, V, W, V, Y, Z, BB, DD**), 0.25 mm (**C, F, I, L, O, R, U, X, AA, CC**).

**Figure 18.**
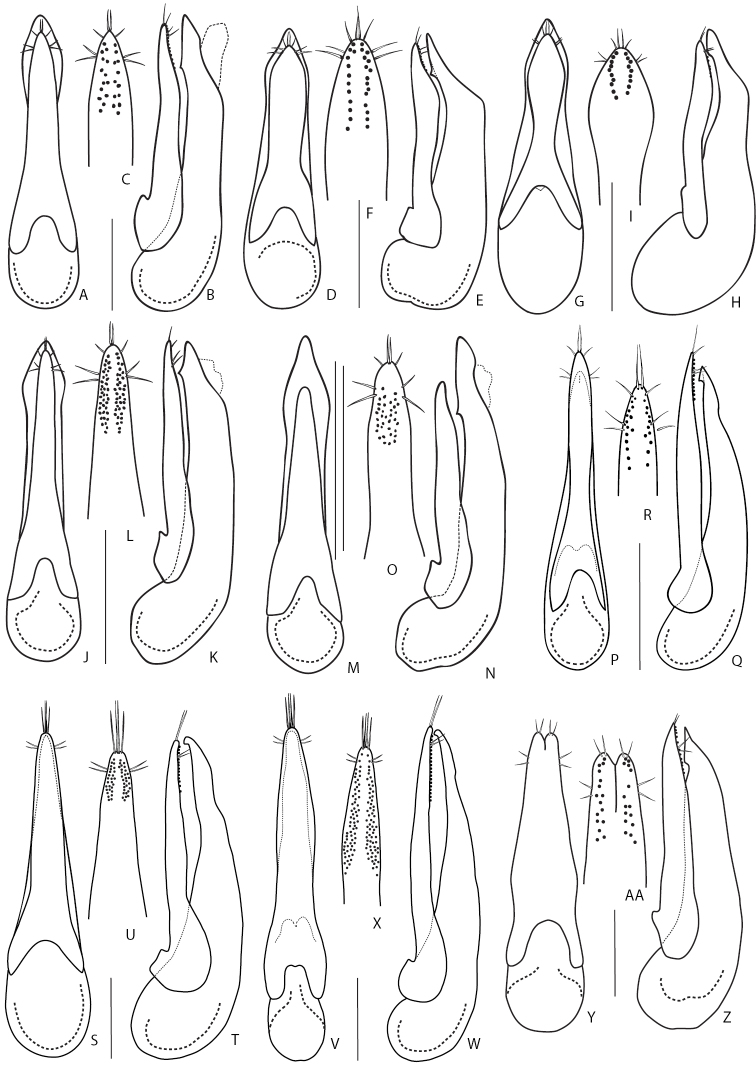
Aedeagi of *Quedius* recorded from Russia: parameral (**A, D, G, J, M, P, S, V**), lateral view (**B, E, H, K, N, Q, T, W**), underside of paramere (**C, F, I, L, O, R, U, X**). *Q.vulneratus* (**A–C**); *Q.lgockii* (**D–F**); *Q.brachypterus* (**G–I**); *Q.obliqueseriatus* (**J–L**); *Q.humosus* (**M–O**); *Q.fumatus* (**P–R**); *Q.suramensis* (**S–U**); *Q.lateralis* (**V–X**). Scale bars 0.5 mm (**A, B, D, E, G, H, J, K, M, N, P, Q, S, T, V, W, V**), 0.25 mm (**C, F, I, L, O, R, U, X**).

### Subgenus Distichalius Casey, 1915

#### Quedius (Distichalius) cinctus (Paykull, 1790)

Fig. 9H–J

*Philonthuslittorinus* Gistel, 1857: 75 Schillhammer 2009: 115 (synonymy)

Very common polytopic species widespread in the West Palearctic (Solodovnikov 2012b). In Russia, widely distributed in the European part from Karelia in the north to Northern Caucasus in the south, reaching the Volga basin in the east. Usually it occurs in various ground-based debris in natural and anthropogenic landscapes (Owen 1999, 2000; Pirugin 2010).

Russia: EUR S–TAIGA (Dedykhin et al. 2005); CN EUR RU (Semionenkov et al. 2015; Semenov 2009; Pirugin 2010); KAREL REP (Horion 1965); MDL VOLGA (Semionenkov et. Al 2015; Semenov 2016; ZIN); N CAUC (Horion 1965; Khachikov 1998; Solodovnikov 1998; ZIN); NW EUR RU (Seidlitz 1875); VOLGO–DON (Khachikov 1998, 2012; Arzanov et al. 2016); unspecified locality: Silfverberg (1992) (northern part of European Russia).

#### Quedius (Distichalius) fusus Cai & Zhou, 2015

Fig. 9E–G

This species was recently described from Dongling Mt. in Mentougou district, Beijing City, in north-eastern China, where it was collected at elevations between 1200 and 1800 m (Cai and Zhou 2015). We here record it from Russia for the first time based on one examined male specimen from Selemdzhinsky District of Amur Province.

Russia: AMUR PROV (cRyv).

#### Quedius (Distichalius) japonicus Sharp, 1874

Fig. 9A–D

This species is widely distributed in Japan (Shibata 1984) and known from the Russian Far East from an unspecified locality. There are no published data on its bionomics.

Russia: unspecified locality in the Far East (Schülke and Smetana 2015).

#### Quedius (Distichalius) kamchaticus Smetana, 1976

Fig. 9N–P

According to published records, this species is restricted to the Kamchatka peninsula. Its bionomics is currently unknown.

Russia: KAMCHATKA (Smetana 1976, 1978b).

#### *Quedius* (*Distichalius) minor* Hochhuth, 1849

Figs 5A, 9K–M

This is a montane species widespread in the Caucasus (Hochhuth 1849; Roubal 1914; Solodovnikov 1998, 2002a) and northern Turkey (Korge 1964, 1971; Coiffait 1978; Solodovnikov 2002a). It is usually found at elevations of 1200–3000 m, mostly in leaf litter in the upper forest zone or in wet ground-based debris in the subalpine and alpine zones, often at the edges of snowfields. In the western Caucasus it is also recorded from lower elevations 300–400 m (Solodovnikov 2002a).

Russia: N CAUC (Khachikov 1998; Solodovnikov 1998, 2002a; ZIN).

### Subgenus Quedius Stephens, 1829

#### Quedius(s. str.)altaicus Korge, 1962

Fig. 11E–H

The distribution of this species stretches across the central and south-western Altai through the border between Russia and Kazakhstan (Korge 1962; Salnitska and Solodovnikov 2018b). All hitherto known specimens were collected at elevations of 1200–2000 m. It was also recorded from Saur Mountains in Kazakhstan (Toleutaev 2014) but such a remote record needs confirmation.

Russia: KUZN ALTAI (Korge 1962; Salnitska and Solodovnikov 2018b; NHMD; ZIN).

#### Quedius(s. str.)balticus Korge, 1960

Figs 5D, 10U–X

This species is distributed in the northern and central regions of Europe where it occurs in various wet ground-based debris, along sea and lake shores. It is considered halophilous or at least tolerant to habitats with high salinity (Coiffait 1978; Solodovnikov 2012b). In Russia it is known only from a few literature records for the southern regions of its European part.

Russia: CRIM REP (Gusarov 1989); VOLGO–DON (Khachikov 1998, 2012; Grebennikov 2001; Arzanov et al. 2004).

#### Quedius(s. str.)curtipennis Bernhauer, 1908

Fig. 9T–V

*Quediuscurtipennis* is considered to be a rather common, polytopic and widely distributed species that is collected from various ground based microhabitats across the entire West Palearctic (Herman 2001). It was also introduced to North America (Smetana 1971, 1978a, 1990). In Spain it was recorded from a cave (Outerelo et al. 1998). However, as stated in Salnitska and Solodovnikov (2018b), its actual distribution, especially outside Europe, requires revision because this species can be easily confused with *Q.fuliginosus* (see below). The data summarized here suggest that *Q.curtipennis* is widespread in the European part of Russia.

Russia: CN EUR RU (Semionenkov et al. 2015); CRIM REP (Koval 1961; Gusarov 1989; Turbanov et al. 2016; ZIN); E CAUC (Khachikov 1998); KAREL REP (Horion 1965); MDL VOLGA (Semenov et al. 2015; ZIN); N CAUC (Khachikov 1998); NW EUR RU (ZIN); VOLGO–DON (Khachikov 1998); unspecified locality in the northern part of European Russia: Silfverberg (1992).

#### Quedius(s. str.)fuliginosus (Gravenhorst, 1802)

Fig. 9Q–S

This is one of the most common species of *Quedius* distributed throughout the entire West Palearctic east to Middle Asia (Salnitska and Solodovnikov 2018b). Its record from Northern China (Horion 1965) is obviously erroneous, because in the latest revision of the Chinese Quediina (Smetana 2017) the subgenus Quedius s. str. was not found in that country at all. In Russia, *Q.fuliginosus* appears to be widely distributed in its European part and extends further east reaching Northern Yenissey and Krasnoyarsk regions. The species is polytopic and can be found in forests and open landscapes, usually in leaf and log litter and different kinds of ground debris (Solodovnikov 2012b); it has been recorded from mole nests (Nowosad 1990), from *Polyporussquamosus* fungi (Semenov et al. 2015a) and from ant nests (Goreslavets 2010, 2016). Because of the strong similarity with *Q.curtipennis* (see above) its literature records from Russia need careful checking.

Russia: ALTAI REP (Psarev 2015); BURYAT REP (Dorzhieva 2015; cSha); CN EUR RU (Kozodoi 1982; Gruntal 2009; Semenov 2009; Pirugin 2010; Troshkova and Troshkov 2014; Semionenkov et al. 2015; cKur; FMNH; NHMD; ZIN; NHMD; ZMMU); CRIM REP. (Nordmann 1837; Gusarov 1989; ZIN); CS EUR RU (Boháč 1988; Semenov 2015; Ruchin 2015; cRyv); E CAUC (Khachikov 1998; cRyv); EUR S–TAIGA (Dedyukhin et al. 2005); IRKUTSK PROV (Shavrin et al. 1999; 2001; Shavrin 2000, 2001; cSha; cSme); KALIN PROV (Alekseev 2014); KAREL REP (cRyv); KRSNYRSK (Sahlberg 1880; Rybalov et al. 2000; cRyv; ZIN); KUZN ALTAI (Babenko 1991; Sushchev et al. 2015; cRyv); MDL OB (Babenko 2016; Babenko and Nuzhnykh 2014); MDL URAL (Sahlberg 1880; Heyden 1880; Smetana 1978b; Uhova 2001; Ermakov 2003; Belskaya and Kolesnikova 2011; cRyv); MDL VOLGA (Krasnobaev 1992; Matveev 2011; Goreslavets et al. 2002; Shulaev and Bogdanov 2008; Semenov and Egorov 2009a; Goreslavets 2010, 2014, 2016a, b; Semenov et al. 2013, 2015a; cRyv; ZIN); N CAUC (Bolov 1969a, b; Khachikov 1998; Solodovnikov 1998; Iljina and Khachikov 2000; Pushkin and Maksimova 2014; Aiydov 2015; Pushkin and Minav 2015; Pushkin 2015, 2016; cRyv; CSha; ZIN); NE EUR RU (Konakova and Kolesnikova 2011a; 2014; 2017); N YENISS (Smetana 1978b; Rybalov et al. 2000; cRyv); NW EUR RU (Poppius 1908; Kolesnikova and Taskaeva 2003; Kolesnikova 2008; Kolesnikova et al. 2010b; Goncharov and Tiunov 2014; ZIN); S URAL (ZIN); SW SIBER (Sahlberg 1880; Pavlov 2002, 2005; Striganova and Porjadina 2005; Buhkalo et al. 2012); VOLGO–DON (Khachikov 2003; Arzanov et al. 2004); unspecified localities: ‘Caucaso’ (Hochhuth 1849); ‘Kaukasus’ (Horion 1965); ‘Sib. med.’ (Smetana 1976); northern part of European Russia (Silfverberg 1992).

#### Quedius(s. str.)levicollis Brullé, 1832

Figs 5B, 10A–D

*Quediuslevicollis* is widespread in the West Palearctic, from Europe to the Middle East (Faldermann 1835; Horion 1965; Smetana 1967, 1978a) and North Africa (Fauvel 1902; Gridelli 1924). The species becomes more rare towards the north. In Russia, it is not common and known only from a few regions of its European part. The species is distinctly thermophilous, avoiding montane areas and preferring open landscapes, especially sandy soils. It can be found in various ground debris on shores or even under seaweeds on beaches (Solodovnikov 2012b); it was also recorded from caves in Sardinia (Bordoni 1982).

Russia: MDL–VOLGA (Shulaev and Bogdanov 2008; ZIN); unspecified localities: ‘Russlands’ (Hochhuth 1862); ‘Caucase et Transcaucase’ (Fauvel 1874).

#### Quedius(s. str.)meridiocarpathicus Smetana, 1958

Fig. 10Q–T

The species is mostly known from south-eastern Europe (Smetana 1958, 1962, 1967, 1993; Horion 1965) and Asia Minor (Coiffait 1961, 1978), but its entire distribution is unclear due to confusion with *Quediusmolochinus* (see below). *Quediusmeridiocarpathicus* prefers various wet ground based debris, mainly in open landscapes.

Russia: CRIM REP (Horion 1965; Gusarov 1989; ZIN); N CAUC (Solodovnikov 1998; Knysh and Solodovnikov 2004; ZIN); VOLGO–DON (Khachikov 1998).

#### Quedius(s. str.)molochinus (Gravenhorst, 1806)

Figs 5E, 10M–P

This is one of the most common *Quedius* s. str. species broadly distributed in the West Palearctic and introduced to North America (Herman 2001). Southern Palearctic records need revision because of the confusion with *Q.meridiocarpathicus* (see above). In Russia, it is also widely distributed, reaching Krasnoyarsk and North Yenissei regions in the east. The record from Zabaikalsky Territory (Shavrin 2000) needs confirmation due to its isolation from the reliably known distribution area. It can be found in leaf litter and various wet ground debris; it was also recorded from a *Talpaeuropaea* nest (Osella and Zanetti 1975), and in association with ants (Goreslavets 2016). Based on the material examined here from the Ural Mountains, the species can be found at rather high elevations, up to 1548 m.

Russia: BURYAT (Shavrin 1998); CN EUR RU (Semenov 2009; Pirugin 2009; Troshkova and Troshkov 2014; Semionenkov et al. 2015; Ruchin 2017; ZIN); EUR S–TAIGA RU (Dedyukhin 2005); IRKUTSK PROV (Shavrin et al. 2001); KAREL REP (cRyv); KRSNYRSK (Veselova and Ryvkin 1991; cRyv); KUZN ALTAI (Babenko 1991); MDL OB (Smetana 1978b; ZIN); MDL URAL (Uhova 2001; Belskaya and Kolesnikova 2011); MDL VOLGA (Goreslavets et al. 2002; Shulaev and Bogdanov 2008; Semenov et al. 2013; Goreslavets 2016; cRyv; ZIN); MURM PROV (cRyv; ZIN); NE EUR RU (Shilov 1975; Kolesnikova and Taskaeva 2003; Kolesnikova and Konakova 2010; Konakova and Kolesnikova 2011a, 2014, 2017; ZIN); NEN–NVZEM (Konakova and Kolesnikova 2014); N YENISS (cRyv; ZIN); NW EUR RU (Seidlitz 1875; Poppius 1908; Kolesnikova 2008; ZIN); S URAL (Voitenkova 2016; LUOMUS); SW SIBER (Buhkalo et al. 2012); ZABAIK TERR (Shavrin 2000); unspecified localities: northern part of European Russia (Silfverberg 1992).

#### Quedius(s. str.)subunicolor Korge, 1961

Fig. 11A–D

The species was hitherto known from Northern Europe (Korge 1961; Palm 1962; Coiffait 1978). Records from Czech Republic, Slovakia, southern Poland and Germany (Smetana 1993; Majzlan et al. 1997; Boháč et al. 2006; Wojas 2006) are obviously misidentifications of the very similar *Q.unicolor*. In Russia, it is known only from the Northern European region. *Quediussubunicolor* can be found in moss in wet habitats (Palm, 1963).

Russia: NE EUR RU (Shilov 1975; ZIN); unspecified locality in the northern part of European Russia: Silfverberg (1992).

#### Quedius(s. str.)sundukovi Smetana, 2003

Figs 5C, 10E–H

*Quediussundukovi* is a clearly brachypterous species with a surprisingly wide distribution. In Russia, it is known from an extensive area from the Far East to south-eastern Siberia. A single record exists from the Altai Mountains in Kazakhstan (Salnitska and Solodovnikov 2018b). The species inhabits regular leaf litter of broad leaved, coniferous, or mixed forests, where usually it can be found in talus-associated debris or moss on the ground. Based on the material examined here, it is also recorded from rather high elevations, up to 2000 m.

Russia: AMUR PROV (cRyv; ZIN); BURYAT REP (NHMD; NHMD; ZIN); IRKUTSK PROV (Smetana and Shavrin 2018); LWR AMUR (Smetana 2003: cRyv; ZIN); SAKHALIN (CNC); ZABAIK TERR (Smetana and Shavrin 2018).

#### Quedius(s. str.)vicinus Ménétriés, 1832

Fig. 10I–L

This species is confined to the southern regions of the West Palearctic from the Middle East (Ghahari et al. 2009; Assing and Feldmann 2012) through Transcaucasia (Ménétriés 1832; Faldermann 1835) to Middle Asia (Coiffait 1978; Boháč 1988; Salnitska and Solodovnikov 2018b). In Russia, it is known only from a literature record from Dagestan Republic. Nothing is known about its bionomics except that it can be found at rather high elevations, up 1500 m (Korge 1971), and in caves (Coiffait 1954, 1955).

Russia: E CAUC (Khachikov 2002).

### Subgenus Microsaurus Dejean, 1833

#### Quedius (Microsaurus) abdominalis Eppelsheim, 1878

Fig. 12U–W

This species is known only from the burrows of *Prometheomysschaposchnikovi* Satunin, 1901, an endemic rodent of the Caucasus (Solodovnikov 2002b), from Western and the south of Central Caucasus regions. It was found in all compartments of the burrow and in the soil around the burrows, at high elevations up to 2400 m (Coiffait 1978; Solodovnikov 1998; 2002b). In Russia, it is known only from Bambak Mountain and Aibga mountain range in the Northern Caucasus region. Its sibling species, *Q.mirus*, is currently known from the burrows of *P.schaposchnikovi* in Georgia and can be distinguished by the structure of the aedeagus (Solodovnikov 2002b).

Russia: N CAUC (Solodovnikov 1998, 2002b); unspecified localities: ‘Caucasus’ (Eppelsheim 1878b).

#### *Quedius (Microsaurus) amplissimus Bernhauer, 1912

This species has not been studied since its original description, which was based on the single female from Crimea “der Umgebung von Sebastopol” (Bernhauer 1912). The author indicated that the species was similar to *Q.brevicornis*. Gridelli (1924) placed this species near *Q.fulgidus* and basically repeated the notes from original description. Jarrige (1971) recorded *Q.amplissimus* from Iran based on the single female without any comments in support of his identification. From the high quality photos of the female holotype available from the Field Museum online beetle type database (FMNH, 2018), one can see that *Q.amplissimus* may be conspecific with *Q.brevicornis*.

#### Quedius (Microsaurus) amurensis Smetana, 2018

Fig. 11U, V

*Quediusamurensis* was recently described from Amur Province (Smetana and Shavrin 2018). The type material was found in leaf litter and mosses at a swampy site and in mixed forest (Smetana and Shavrin 2018).

Russia: AMUR PROV (Smetana and Shavrin 2018).

#### Quedius (Microsaurus) brevicornis (Thomson, 1860)

Fig. 13J–L

*Quediusbrevicornis* is distributed through the whole territory of Central and Northern Europe where it is especially abundant in the north (Solodovnikov 2012b). In Russia, it is also known only from the European part. It usually inhabits debris of decaying wood and old hollow trees (Legner and Moore 1977; Smetana 1958). It was also recorded from mole nests (Nowosad 1990).

Russia: CN EUR RU (Nikitsky and Schigel 2004; Semionenkov et al. 2015; cRyv); CS EUR RU (Horion 1965); N CAUC (Jablokov–Khnzorian 1975); unspecified localities: ‘Russie’ (Fauvel 1874); northern part of European Russia (Silfverberg 1992).

#### Quedius (Microsaurus) brevis Erichson, 1840

Figs 6A, 12O–Q

The species is widely distributed in the Palearctic from Europe (Solodovnikov 2012b) to the Russian Far East. In Russia, it is known from sparse records through its whole territory. *Quediusbrevis* is a myrmecophilous species confined to the nests of ants mostly of the genus *Formica* (Grimm 1845; Janák & Vysoky´ 1992) or sometimes *Lasius* (Smetana 1958).

Russia: CN EUR RU (Semenov 2009; Semionenkov et al. 2015; NHMD; ZIN); EUR S–TAIGA RU (Dedykhin et al. 2005; ZIN); IRKUTSK PROV (cSha, ZIN); LWR AMUR (cRyv); MDL VOLGA (Goreslavets et al. 2002; Shulaev and Bogdanov 2008; Goreslavets 2010, 2016, 2016b; Semenov et al. 2015); NW EUR RU (Seidlitz 1875; Poppius 1908; Savelyeva and Dolgin 2009; ZIN); S YAKUT (cRyv); VOLGO–DON (ZIN); unspecified localities: ‘Russlands’ in Hochhuth (1862); northern part of European Russia (Silfverberg 1992).

#### Quedius (Microsaurus) citelli Kirschenblat, 1933

Fig. 12R–T

The species was hitherto known only from the type locality Adun–Tshelon Mountain Ridge in Zabaikalsky Territory (Kirshenblat 1933). It was collected in the burrow of the ground squirrel *Spermophilusdauricus* Brandt, 1843. Boháč (1988) illustrated an aedeagus of the specimen that he claimed to be the type of *Q.citelli*. Kirshenblat (1933) did not specify the number of the type specimens and from his description it is only clear that he had more than one specimen. We were able to find one male and two female specimens with the labels “Adun–Tshelon plemchoz. Zabaik. Bytshkov VIII.929/ burrow of ground squirrel/ *Quediuscitelli* sp. nov. Kirshenblat det” which are undoubtedly syntypes of *Q.citelli*. However, the male syntype was intact (not dissected by Boháč) and our examination of its aedeagus showed that its structure (Fig. 12R–T) is completely different from the description and illustration provided by Boháč (1988). Potentially, Boháč (1988) had dissected another syntype specimen which we did not find and in this case, two species would be hiding under *Q.citelli*. Alternatively, he has illustrated the aedeagus of another species based on some other material.

*Lectotypedesignation*: to avoid confusion and fix the identity of *Q.citelli*, here we designate one male syntype as a lectotype rendering two mentioned female paralectotypes.

Russia: ZABAIK TERR (Kirschenblat 1933).

#### Quedius (Microsaurus) conviva Smetana, 2018 in Smetana and Shavrin (2018)

Fig. 12M, N

The species is currently known from the type locality in East Siberia: Irkutsk Area, Angarsk. The type specimens were collected from the burrows of *Urocitellusundulatus* (Pallas 1778) at the edge of a *Pinussylvestris* forest, with grasses (*Calamagrostis* spp.) on sandy soil with alluvium (Smetana and Shavrin 2018).

Russia: IRKUTSK PROV (Smetana and Shavrin 2018).

#### Quedius (Microsaurus) cruentus (Olivier, 1795)

Fig. 14A–C

*Philonthusputridarius* Gistel, 1857: 19; Schillhammer 2009: 115 (synonymy).

*Quediuscruentus* is a common and widely distributed West Palearctic species (Solodovnikov 2012b) that was introduced to the Oriental Region (Kraatz 1859; Fauvel 1874; Cameron 1932; Coiffait 1978) and North America (Gusarov 2001). In Russia, it is widely distributed in its European part. It is a rather polytopic and widespread species, which can be found in various ground based debris, usually associated with decaying wood (Solodovnikov 2012b). It was also recorded from a cave (Jeannel and Jarrige 1949) and on fungi (Voitenkova 2016).

Russia: CN EUR RU (Nikitsky and Schigel 2004; Semionenkov, et al. 2015; Voitenkova 2016); CRIM REP (Gusarov 1989; ZIN); CS EUR RU (Semenov 2015; Ruchin 2017; ZMMU; ZIN); EUR S-TAIGA RU (Dedykhin et al. 2005); KAREL REP (Horion 1965); MDL VOLGA (Semenov et al. 2015); N CAUC (Khachikov 1998; ZMMU; ZIN); NW EUR RU (Seidlitz 1875; ZIN); VOLGO–DON (Khachikov 1998; Arzanov et al. 2004); unspecified localities: ‘Russie; le Caucase’ (Fauvel 1874); ‘Caucasus’ (Ganglbauer 1895); northern part of European Russia (Silfverberg 1992).

#### Quedius (Microsaurus) edmundi Coiffait, 1969

Fig. 13G–I

*Quediusedmundi* is endemic to the North-Western Caucasus and was described (Reitter 1909) and further recorded (Coiffait 1967, 1978) from Georgia. In Russia, this species is known from the Western and Northern Caucasus only. Its bionomics are barely known, but based on a few records (Solodovnikov 1998; NHMD) it can be found in leaf litter.

Russia: N CAUC (Solodovnikov 1998; cSme; NHMD).

#### Quedius (Microsaurus) fasciculatus Eppelsheim, 1886

Figs 6B, 12X–Z

The species is currently known from Russia only, from the Far East and East Siberia. Based on the material examined here, it can be found in various decaying wood. Also it was recorded from a nest of the Siberian chipmunk *Eutamiassibiricusasiaticus* (Gmelin 1788) (ZIN).

Russia: AMUR PROV (cKur; ZIN); BURYAT REP (Smetana 1978b; Shavrin 2000); IRKUTSK PROV (Roubal 1914; Shavrin 2001); LWR AMUR (Eppelsheim 1886; ZMMU); PRIM TERR (CNC; ZMMU); S KURIL (NHMD); S YAKUT (Smetana 1978b); ZABAIK TERR (Shavrin 2000; cSha).

#### Quedius (Microsaurus) fulgidus (Fabricius, 1793)

Fig. 14G–I

*Quediusfulgidus* is widely distributed in the West Palearctic and it is one of several cosmopolitan *Quedius* introduced to North and South America, Oriental region, Australia and New Zealand (Herman 2001). In Russia, however, its wide range stretches only through its European part to East Siberia. *Quediusfulgidus* is not recorded from the Russian Far East. Its microhabitats vary from leaf litter and similar ground based debris to decaying wood. It can also be found in caves (Jeannel and Jarrige 1949; Bordoni 1982), ant nests (Shulaev and Bogdanov 2008), and is overall common in synanthropic habitats (Solodovnikov 2012b).

Russia: CN EUR RU (Semionenkov et al. 2005; ZIN); CRIM REP (Gusarov 1989; Turbanov et al. 2016; ZIN); CS EUR RU (ZIN); EUR S–TAIGA RU (Dedykhin et al. 2005; ZIN); IRKUTSK PROV (ZIN); KAREL REP (Horion 1965); KUZN ALTAI (Heyden 1880; Babenko 1991); LWR VOLGA (Khachikov 1998; Grebennikov 2001); MDL OB (Heyden 1880); MDL VOLGA (Gridelli 1929; Shulaev and Bogdanov 2008; Semenov et al. 2015; Goreslavets 2016b); NW EUR RU (Seidlitz 1875); S URAL (Gridelli 1924); VOLGO–DON (Khachikov 1998); ZABAIK TERR (Horion 1965); unspecified localities: ’Rosia merid. et orientalis’ (Hochhuth 1862); northern part of European Russia (Silfverberg 1992).

#### Quedius (Microsaurus) infuscatus Erichson, 1840

Fig. 11O–Q

*Quediusinfuscatus* is widely distributed throughout Europe (Herman 2001; Gamarra et al. 2011; Assing 2016), where it is more common in its central part (Solodovnikov 2012b). Records from Middle Asia (Kascheev 1984, 1985) were considered ambiguous in the revision by Salnitska and Solodovnikov (2018b). In Russia, it is known from the southern regions of its European part. The species inhabits old trees and decaying wood debris (Roubal 1941; Smetana 1993).

Russia: CRIM REP (Gusarov 1989); MDL VOLGA (Shulaev and Bogdanov 2008; cRyv); N CAUC (Roubal 1911); unspecified locality: ‘Kaukasus’ (Horion 1965).

Notes: *Quediuskvashei* described by Khachikov (2005) is identical with *Q.infuscatus*. It will be synonymized with the latter in our separate paper on the entire species group, currently in preparation.

#### Quedius (Microsaurus) invreae Gridelli, 1924

Figs 6D, 14M–O

Based on Assing (2019), distribution of this species needs clarification because of the earlier confusion with *Q.puncticollis*. It is reliably known from southern Europe and Transcaucasia and presumably it is a widespread species in the south-westrn Palaearctic where it was erroneously recorded as *Q.puncticollis*. In Russia, it is also known from the southern regions of its European part. Its bionomics is poorly known, but apparently (Assing 2019) it is not a nidicolous species, unlike *Q.puncticollis*. Based on a few records from Russia provided here, it can be found in leaf litter.

Russia: MDL VOLGA (Goreslavets et al. 2002; Goreslavets 2010; ZIN); VOLGO–DON (Khachikov 1998); N CAUC (cRyv; ZIN).

#### *Quedius (Microsaurus) koltzei Eppelsheim, 1887

Fig. 13A–C

This species was described from Khabarovsk in the Russian Far East (Eppelsheim 1887). Recently, it was also recorded from Kazakhstan (Coiffait 1978) and China (Smetana 2015). Currently, the distribution and identity of this species remain ambiguous pending more material for study (Salnitska and Solodovnikov 2018b).

Russia: LWR AMUR (Eppelsheim 1887).

#### *Quedius (Microsaurus) kvashei Khachikov, 2005

*Quediuskvashei* was described based on a single male specimen from Rostov Province (Khachikov 2005). The author indicated that the species is very similar to *Q.infuscatus*, from which it can be distinguished by unicolorous coloration of elytra and the structure of aedeagus. We have examined the holotype of *Q.kvashei* and it is clear that the species is identical with *Q.infuscatus*. We will formally introduce this synonymy in a separate paper treating the entire *Q.infuscatus* group of species, which is currently in preparation.

Russia: VOLGO–DON (Khachikov 2005).

#### Quedius (Microsaurus) longicornis Kraatz, 1857

Fig. 12A–C

The species is widely distributed in Europe, but not common (Solodovnikov 2012b). In Russia, it is known mainly from its European part with the easternmost record from the South-West Siberian region. Records from the Caucasus are ambiguous and need confirmation. Usually it can be found in forests: in leaf litter, decaying wood. Also, it was found in caves (Jeannel and Jarrige 1949) and in mole (Potockaja 1967; Osella and Zanetti 1975; Nowosad 1990) and other small mammals (Solodovnikov 2012b) nests.

Russia: CN EUR RU (Semionenkov et al. 2015; ZMMU); EUR S–TAIGA RU (Dedykhin et al. 2005); MDL URAL (Belskaya and Kolesnikova 2011); MDL VOLGA (Shulaev and Bogdanov 2008; Matveev 2011; Semenov et al. 2013); N CAUC (Khachikov 1998; Solodovnikov 1998; ZIN); NW EUR RU (cRyv); SW SIBER (Buhkalo et al. 2012; ZMMU); unspecified localities: ‘Kaukasus’ (Horion 1965); northern part of European Russia (Silfverberg 1992).

#### Quedius (Microsaurus) lundbergi Palm, 1973

Fig. 11R–T

The species was hitherto known from the original description based on material from Sweden (Palm 1973). Here we report the first record of this species from Russia, where it was collected in the village Cherbi (Tuva Republic) at an elevation of ~800 m. Presumably it is a widespread boreal species.

Russia: TUVA REP (ZIN).

#### Quedius (Microsaurus) maurus (Sahlberg, 1830)

Fig. 13P–R

The species is known mostly from central and northern Europe, and from the mountain areas of southern Europe; it is absent in the Mediterranean region, but recorded from Turkey (Korge 1964) and the Caucasus (Coiffait 1978; Solodovnikov 2012b). In Russia, it is distributed in its European part, east to Middle Volga region. *Quediusmaurus* can be found from the lowlands to the subalpine zone of mountains, mainly in forested landscapes. It inhabits various ground based debris, but is also recorded from decaying wood (Semenov 2010; Semenov et al. 2015) and mole burrows (Osella and Zanetti 1975; Nowosad 1990).

Russia: CN EUR RU (Semenov 2010; Semionenkov et al. 2015; ZIN, ZMMU); CS EUR RU (Semenov 2015; Ruchin 2017); EUR S–TAIGA RU (Dedykhin et al. 2005); KAREL REP (Horion 1965); MDL VOLGA (Semenov et al. 2015); N CAUC (Roubal 1911; Horion 1965; ZIN); NW EUR RU (Seidlitz 1875; Horion 1965; Zagidullina et al. 2010; ZIN); unspecified localities: ‘Russie septentrionale’ (Fauvel 1874); ‘Caucasus’ (Ganglbauer 1895); northern part of European Russia (Silfverberg 1992).

#### Quedius (Microsaurus) mesomelinus (Marsham, 1802)

Figs 6C, 13M–O

*Quediusmesomelinus* is a widely distributed transpalearctic species, which has been introduced to Greenland, North and South America and to the Australian region. It is considered boreo-montane and is confined to the northern part of the Palearctic and to the mountains in the south (Herman 2001; Solodovnikov 2012b). In Russia, it is distributed from the European part to the Far East but not recorded from the southern regions. *Quediusmesomelinus* can be found in forested and open landscapes, in various ground debris, sometimes in caves (Bordoni and Oromi 1998; Outerelo et al. 1998), in mammal nests or burrows (Nowosad 1990), in ant nests (Goreslavets 2016), on fungi (Voitenkova 2016; cRyv) and in basements or other shady human constructions (Ryabukhin 1999; Solodovnikov 2012b).

Russia: CN EUR RU (Kochetova et al. 2011; Troshkov and Nikitsky 2015; Semenov 2015; Semionenkov et al. 2015; Voitenkova 2016; Ruchin 2017; cKur; ZIN; ZMMU); EUR S–TAIGA RU (Dedykhin et al. 2015); IRKUTSK PROV (Shavrin 2001); KALIN PROV (Alekseev 2014; ZIN); KAMCHATKA (Ryabukhin 1999; 2008, 2010; Lobkova and Semenov 2014, 2017); KAREL REP (Sahlberg 1876); MAGADAN PROV (Ryabukhin 1999); MDL URAL (ZIN); MDL VOLGA (Goreslavets et al. 2002; Goreslavets 2010, 2014, 2016; Shulaev and Bogdanov 2008; cRyv); NE EUR RUS (Shilov 1975; ZIN); N CAUC (Roubal 1911); NW EUR RU (cRyv; ZIN); NW YAKUT (ZIN); PRIM TERR (Horion 1965); S KURIL (Shibata et al. 2006); VOLGO–DON (Khachikov 1998); ZABAIK TERR (Horion 1965); unspecified localities: ‘northern, north-western and central regions of the European part of USSR’ (Potockaja 1967); northern part of European Russia (Silfverberg 1992).

#### Quedius (Microsaurus) microps Gravenhorst, 1847

Fig. 11L–N

The species is widely distributed in West Palaearctic. In Europe, it occurs everywhere except the Iberian Peninsula; it is absent in North Africa. In Russia, *Q.microps* is known from a few regions in the European part and from South-West Siberia region. It is usually found in mammal nests (Nowosad 1990; Solodovnikov 2012b), but also recorded from [probably old] dung (Voitenkova 2016).

Russia: CN EUR RU (Dorofeev 2013; Semionenkov et al. 2015); EUR S–TAIGA RU (Dedykhin et al. 2005); KUZN ALTAI (Zinchenko 2003); N CAUC (Khachikov 1998); SW SIBER (Voitenkova 2003); unspecified locality: ‘northern part of European Russia’ (Silfverberg 1992).

Notes: Veselova and Ryvkin (1991) recorded *Q.* sp. nov. pr. *microps* from Krasnoyarsk region of Russia, but examination of that material is needed to clarify the identity of that species.

#### [Quedius (Microsaurus) nigrocaeruleus Fauvel, 1876]

Fig. 14J–L

This nidicolous species, confined to mole nests, is distributed in Europe, except its northern part, and in North Africa. It is more common in the western part of its range (Solodovnikov 2012b). There is only one dubious record from European Russia for *Q.nigrocaeruleus* (Potockaja 1976), but unfortunately without any locality data.

Russia: ‘European Russia’ (Potockaja 1976).

#### Quedius (Microsaurus) ochripennis (Ménétriés, 1832)

Fig. 14D–F

The species is widely distributed in the West Palearctic, including the Mediterranean and North Africa (Solodovnikov 2012b), and is also recorded from the Oriental region (Cameron 1932). In Russia, it is known only from its European part, and from South-West Siberia based on the easternmost record in Pavlov (2005). *Quediusochripennis* is a polytopic species occurring in various ground-based debris and is often associated with decaying wood and the nests of mammals, wasps and ants (Potockaja 1967; Solodovnikov 2012b).

Russia: CRIM REP (Gusarov 1989; ZIN); CS EUR RU (ZIN); MDL URAL (ZIN); N CAUC (Bolov 1969a, b; Khachikov 1998; Solodovnikov 1998; Knysh and Solodovnikov 2004); NE EUR RU (Shilov 1975); SW SIBER (Pavlov 2005); unspecified localities: ‘Russie, Caucase’ (Fauvel 1874); ‘Kaukasus’ (Horion 1965); ‘central, southwest and southern regions’ (Potockaja 1967).

#### Quedius (Microsaurus) puncticollis (Thomson, 1867)

Fig. 14P

*Quediusrubripennis* Bernhauer, 1901: 652; Solodovnikov 2002a: 141 (synonymy).

Based on Assing (2019), distribution of this species needs clarification because of the earlier confusion with *Q.invreae*. It is reliably known from the northern part of Central Europe and presumably it is less widespread species than *Q.puncticollis*. It is a nidicolous species that prefers mammal nests (Osella and Zanetti 1975; Nowosad 1990; Semenov et al. 2015; Assing 2019). The Russian records of this species where it was reported throughout its European part, West Siberia and from Kuznetsky Altai, need revision.

Russia: CN EUR RU (cRyv); EUR S–TAIGA RU (Dedykhin et al. 2005); KUZN ALTAI (Zinchenko 2003); MDL VOLGA (Semenov et al. 2015); N CAUC (Bolov 1969 a, b; Solodovnikov 2002a); NE EUR RU (Mannerheim 1830, 1831); SW SIBER (Buhkalo et al. 2012); VOLGO–DON (Grebennikov 2001; Khachikov 2012; Sazhnev and Halilov 2015; Arzanov 2016); unspecified locality in northern part of European Russia (Silfverberg 1992).

#### Quedius (Microsaurus) repentinus Salnitska & Solodovnikov, 2018

Fig. 12G–I

This hypogean species is known only from the type locality in Altai Republic: Turochansky Distr., Mountain Evrechala (south-eastern Altai). The type specimens were collected at elevations of 1850–2050 m in an old talus formation covered by fine detrital rock with lichens (Salnitska and Solodovnikov 2018a).

Russia: ALTAI REP (Salnitska and Solodovnikov 2018a).

#### Quedius (Microsaurus) roma Solodovnikov & Hansen, 2016

Fig. 12D–F

*Quediusroma* is a recently described hypogean species from Mt. Ko in Central Sikhote-Alin and hitherto known only from the original description. The type material was collected from humus between small rocks of the upper levels of the talus at lower elevations ca. 750 m (Solodovnikov and Hansen 2016).

Russia: LWR AMUR (Solodovnikov and Hansen 2016).

#### Quedius (Microsaurus) scitus (Gravenhorst, 1806)

Fig. 13D–F

*Bolitobiuspunctulatus* Heer, 1839: 298; Schülke 2004: 933 (synonymy).

The species is distributed throughout Europe but is quite rare; it is not recorded from North Africa (Solodovnikov 2012b). In Russia, it is known from its European part, but also recorded from Irkutsk Province. Usually, it can be found in decaying wood debris from holes of old trees (Legner and Moore 1977; Owen 1999, 2000; Semenov 2009), often in association with ants.

Russia: CN EUR RU (Semenov 2009; Semionenkov et al. 2015; ZIN); CS EUR RU (Semenov 2015; Ruchin 2017 given by Semenov 2015); EUR S–TAIGA RU (Dedykhin et al. 2005); IRKUTSK PROV. (Shavrin et al. 1999); MDL VOLGA (Semenov et al. 2015a; Semenov 2016); N CAUC (ZIN); NW EUR RU (ZIN); unspecified localities and dubious records: ‘Russia’ (Nordmann 1837); ‘Kaukasus’ (Horion 1965); ‘north west and south west [Russia]’ (Potockaja 1967); northern part of European Russia (Silfverberg 1992).

#### Quedius (Microsaurus) sofiri Khachikov, 2005

Fig. 11W–Y

Khachikov (2005) described *Q.sofiri* based on a single female specimen from Rostov Province. He compared *Q.sofiri* with *Q.infuscatus*, from which it can be distinguished by unicolorous coloration and punctation of elytra. Also he mentioned that *Q.sofiri* differs from *Q.kvashei* (described in the same paper, here placed in synonymy with *Q.infuscatus*) by the wider (1.5–2 times as wide as long) penultimate antennal segments and sparser punctation of scutellum (only 2–3 punctures) and elytra. We examined the holotype of *Q.sofiri* and verified that the diagnostic characters indicated by Khachikov (2005) for *Q.sofiri* are accurate. Also we were able to examine one male specimen from Northern Turkey which is identical in external morphology to the holotype of *Q.sofiri*. A full redescription of *Q.sofiri* will be provided in our separate paper, which is in preparation. Here we provide the first illustrations of the aedeagus for this species based on the specimen from Turkey (Fig. 11W–Y).

Russia: VOLGO–DON (Khachikov 2005).

#### Quedius (Microsaurus) tenellus (Gravenhorst, 1806)

Figs 5F, 12J–L

This is a widespread and rather common transpalearctic species (Solodovnikov 2012b; Lobkova and Semenov 2014). It is recorded throughout Russia, from its European part to Magadan region. Usually it is confined to forests, especially coniferous, where it can be found in leaf litter, moss or in old mouse nests (Solodovnikov 2012b).

Russia: ALTAI REP (ZIN); AMUR PROV (cKur); BURYAT REP (Smetana 1995; cSch); CN EUR RU (ZIN); IRKUTSK PROV (Heyden 1896; Gridelli 1924; Shavrin and Anischenko 1998; Shavrin et al. 1999; cSha); KAMCHATKA (Smetana 1978b; Ryabukhin 1999, 2008; Lobkova and Semenov 2014; cRyv); KAREL REP (Horion 1965); KUZN ALTAI (Babenko 1991); LWR AMUR (cRyv); MAGADAN PROV (LUOMUS); MDL URAL (Horion 1965; Ermakov 2003); N CAUC (ZIN); NW EUR RU (Horion 1965); PRIM TERR (Coiffait 1974,); TUVA REP (cRyv); unspecified localities: ‘et bords du lac Baical’ (Fauvel 1874); ‘Sibirien’ (Ganglbauer 1895); ‘Kaukasus’ (Horion 1965); ‘de la Russie et la Siberie’ (Coiffait 1978); northern part of European Russia (Silfverberg 1992).

#### *Quedius (Microsaurus) tetrapunctatus Coiffait, 1977

Fig. 13S–U

This species was described and hitherto known from Armenia (Jablokov-Khnzoria 1961; Coiffait 1969, 1977). There is only one dubious record from Russia. This species needs a revision.

Russia: VOLGO–DON (Khachikov 1998).

#### Quedius (Microsaurus) truncicola Fairmaire & Laboulbène, 1856

Fig. 11I–K

This species is widely distributed in Europe, especially Central Europe, but not common and the records are very scattered (Solodovnikov 2012b). In Russia, it is known only based on the literature record from the lowlands of the Middle Volga region (Goreslavets et al. 2002). The species usually can be found in debris and holes of old trees; its detailed biology is described in Sörensson (1996).

Russia: MDL VOLGA (Goreslavets et al. 2002); unspecified locality: ‘Nordrussl., Südrussland’ (Horion 1965).

#### Quedius (Microsaurus) vexans Eppelsheim, 1881

Fig. 13V–X

The species is quite rare and occurs mainly in Central Europe. In Russia, it is also known mainly from its European part, but also recorded from Krasnoyarsk region in Khakassia Republic (Janovsky et al. 1998). *Quediusvexans* prefers the nests of small mammals (Smetana 1957; Potockaja 1967; Nowosad 1990).

Russia: CN EUR RU (Semionenkov et al. 2015); CRIM REP (Gusarov 1989; ZIN); EUR S–TAIGA RU (Dedykhin et al. 2005); KRSNYRSK (Janovsky et al. 1998); unspecified locality: ‘central regions [of European Russia]’ (Potockaja 1967).

#### Quedius (Microsaurus) xanthopus Erichson, 1839

Fig. 13Y–AA

The species is widespread in the Palearctic, but in East Siberia and Russian Far East it is known only from old literature records, which need verification. *Quediusxanthopus* usually can be found in decaying wood or under bark (Legner and Moore 1977; Semenov 2009; Semenov et al. 2015), often on fungi (Hågvar 1999; Vinogradova et al. 2010).

Russia: CN EUR RU (Semenov 2009; Kochetova et al. 2011; ZMMU; ZIN); CS EUR RU (Horion 1965; Semenov 2014; Ruchin 2015); EUR S–TAIGA RU (Dedykhin et al. 2015); KAREL REP (cRyv); MDL URAL (Horion 1965; ZIN); MDL VOLGA (Shulaev 2008; Vinogradova 2010; Goreslavets 2010; Semenov et al. 2009a, 2015; Semenov 2016, 2017; ZIN); NW EUR RU (Seidlitz 1875; Zagidullina 2010; ZIN); PRIM TERR (Horion 1965); ZABAIK TERR (Horion 1965); unspecified locality: ‘Russie et sur les bords du Baikal’ (Fauvel 1874); ‘widespread’ (Potockaja 1976); northern part of European Russia (Silfverberg 1992).

### Subgenus Raphirus Stephens, 1829

#### Quedius (Raphirus) aedilis Smetana, 2018 in Smetana and Shavrin (2018)

Fig. 16I–J

This species was recently described from Sikhote–Alin Nature Reserve (Smetana and Shavrin 2018) and here we have seen additional specimens from Primorsky Territory. Bionomics is unclear, because all material was collected using pan or pitfall traps. The specimens we were able to study were collected at a rather high elevation of 1300–1500 m in pine leaf litter.

Russia: PRIM TERR: (Smetana and Shavrin 2018; CNC).

#### *Quedius (Raphirus) angaricus Coiffait, 1975

Coiffait (1975) described *Quediusangaricus* from ‘Listvianka, région sud-ouest du Lac Baïkal’ in Irkutsk province based on female specimens. He mentioned that the species is close to *Q.umbrinus*, but can be distinguished from the latter by the very short (as wide as long) and densely punctate elytra. Since we examined neither the type, nor we found any additional material of this species, its identity remains unclear.

Russia: IRKUTSK PROV (Coiffait 1975).

#### Quedius (Raphirus) boopoides Munster, 1923

Fig. 15V–X

This species is considered as wide-spread in Europe, but its real distribution is unclear due to confusion with *Q.boops* (Solodovnikov 2012b). In Russia, it is more common in its northern and central European parts, absent in the south, and becomes more rare eastwards with the easternmost records from Irkutsk and Zabaikalsky regions. *Quediusboopoides* can be found in wet ground-based debris and especially in moss in the forests (Solodovnikov 2012b). Further comments on the identity and composition of the *Q.boops*-group of species are provided in the introductory Taxonomy section.

Russia: CN EUR RU (Semenov 2009; Semionenkov et al. 2015); IRKUTSK PROV (cSha); KRSNYRSK (cRyv); MDL OB (Babenko 2016; cRyv); MDL URAL (Uhova 2001); MDL VOLGA (Shulaev and Bogdanov 2008); MURM PROV (cRyv; ZIN); N CAUC (ZIN); NE EUR RU (Shilov 1975; Konakova and Kolesnikova 2011); NW EUR RU (ZIN); SW SIBER (Buhkalo et al. 2012); ZABAIK TERR (cSha); unspecified localities: ‘NordRußland’ (Horion 1965); northern part of European Russia (Silfverberg 1992).

#### Quedius (Raphirus) boops (Gravenhorst, 1802)

Fig. 15S–U

*Philonthusboopstauricus* Nordmann, 1837: 78;

*Quediuscrius* Tottenham, 1948: 258;

*Quediusboopsislandicus* Fagel, 1960: 113; Assing 2017: 1036 (synonymy).

*Quediusboops* is a transpalearctic species distributed from Europe to the Russian Far East (Herman 2001; Solodovnikov 2012b). In Russia, it occurs everywhere, but is more common in its European part and becomes more rare towards the east, where its easternmost record is known from Lower Amur region. The species inhabits various wet ground based debris such as leaf litter, moss, hay, plant residues in forested and open landscapes (Solodovnikov 2012b; material examined here). Further comments on the identity and composition of the *Q.boops*-group of species are provided in the introductory Taxonomy section.

Russia: BURYAT REP (cRyv); CN EUR RU (Pirugin 2010; Semionenkov et al. 2015; ZIN); CRIM REP (Nordmann 1837; Gusarov 1989; ZIN); EUR S–TAIGA RUS (Dedykhin et al. 2005); IRKUTSK PROV (Poppius 1909; Shavrin 2001; cSha); KRSNYRSK (Veselova and Ryvkin 1991; ZIN); KUZN ALTAI (Babenko 1991; Sushchev et al. 2015); LWR AMUR (cRyv); MDL OB (Babenko and Nuzhnykh 2014; cRyv); MURM PROV (cRyv; ZIN); N CAUC (Khachikov 1998; Solodovnikov 1998; Knysh and Solodovnikov 2004; ZIN); N YENISS (Sahlberg 1880; Poppius 1909; cRyv); NE EUR RU (Shilov 1975); NW EUR RU (cRyv; ZIN); NW YAKUT (Poppius 1909); SW SIBER (Striganova and Porjadina 2005); ZABAIK TERR (cRyv); unspecified localities: ‘Sibirien’ (Horion 1965); northern part of European Russia (Silfverberg 1992).

Notes: *Quediusacuminatus* was described from the unspecified locality ‘Kaukasus’ (Hochhuth 1849). Later the species was recorded from the Central and South Europe, Turkey, Armenia and Lebanon (Fauvel 1874; Horion 1965; Coiffait 1967, 1978 etc.), but never from Russia. In our revision of the Middle Asian *Quedius* (Salnitska and Solodovnikov 2018b) records of *Q.acuminatus* from that region were recognized as doubtful. *Quediusacuminatus* undoubtedly belongs to the *Q.boops* group, but as indicated in the discussion about that group in the introductory Taxonomy section here, the borders between species there need clarification. Presumably, *Q.acuminatus* is a synonym of one of the currently recognized species in that group. Its type material, therefore, must be considered in a comprehensive revision of *Q.boops* and alike.

#### [Quedius (Raphirus) brachypterus Coiffait, 1967]

Fig. 18G–I

This brachypterous species is currently known only from the holotype from the Caucasus (Coiffait 1967), for which there is no clear locality or bionomic data. It may well be that it does not occur in Russia. Details about the type specimen, redescription, and comparison of the species can be found in Solodovnikov (2004).

Unspecified locality: ‘Kaukas’ (Coiffait 1967).

#### Quedius (Raphirus) centrasiaticus Coiffait, 1969

Fig. 15D–F

This species is known only from the type locality in Altai at Teletskoe Lake (Coiffait 1969, 1978) and our first new provincial record from the Nizhneudinsky District of Irkutsk Province. Bionomics unknown.

Russia: ALTAI REP (Coiffait 1969); IRKUTSK PROV (cSha; cRyv).

#### [Quedius (Raphirus) cincticollis Kraatz, 1857]

Fig. 16Q–S

This montane species is known from the European mountains such as eastern Alps, Carpathians, and north-western Balkans (Solodovnikov 2012b). Russian records from Kuznetksy Altai and North Eastern European regions are questionable. The species can be found in leaf litter and other kinds of ground debris of montane forests, usually around the timber line (Solodovnikov 2012b).

Russia: KUZN ALTAI (Babenko 1991); NE EUR RU (Shilov 1975).

#### Quedius (Raphirus) fellmani (Zetterstedt, 1838)

Figs 6E, 15M–O

*Quediusfellmani* is a widely distributed species confined to the arctoboreal circle of the Holarctic region: Noth America, Europe, and Asia (Herman 2001; Ryabukhin 2008, 2010). In Russia, the species is rather widespread and also more common in the northern regions (Ryabukhin 1999, 2008, 2010; material examined here). It inhabits forest and scrubs leaf litter, and occurs in moss and lichen cover of lowland tundra; also it can be found under stones, in rotten plants and other ground based wet debris in meadows (Ryabukhin 1999).

Russia: ALTAI REP (cRyv); CHUKOTKA (Ryabukhin 1999); CN EUR RU (ZMMU); IRKUTSK PROV (Shavrin et al. 1999; cSme; cSha); KAMCHATKA (Smetana 1995, 1978b; Ryabukhin 1999, 2008, 2010; cRyv; ZIN); KRSNYRSK (Veselova and Ryvkin 1991; cRyv); LWR OB (Olshvang 1992; Chernov et al. 2014 (given by Olshvang 1992); Striganova and Porjadina 2005; cRyv); N YENISS (cRyv); NW YAKUT (Smetana 1978b); MAGADAN PROV (Ryabukhin 1999); S YAKUT (Smetana 1978b; ZIN); ZABAIK TERR (cSha); unspecified locality: northern part of European Russia (Silfverberg 1992).

#### Quedius (Raphirus) fulvicollis (Stephens, 1832)

Fig. 14T–V

This is a widely distributed arctoboreal Holarctic species that occurs in many countries of Europe, in Russia, Canada, and USA (Herman 2001; Ryabukhin 2008, 2010). In Russia, it is a common northern species (Ryabukhin 1999, 2008, 2010) with a biology similar to that of *Q.fellmani*. However, *Q.fulvicollis* usually prefers wetter habitats around bogs and rivers (Ryabukhin 1999; material examined here).

Russia: BURYAT REP (cSha); CHUKOTKA (Ryabukhin 1999); CN EUR RU (Semionenkov et al. 2015); IRKUTSK PROV (Shavrin et al. 1999; cSha; ISEA); KAMCHATKA (Bernhauer 1926; Smetana 1976; Ryabukhin 1999, 2008, 2010; Lobkova and Semenov 2017 (given by Ryabukhin 1999); cRyv; ZMMU); KRSNYRSK (cRyv); MAGADAN PROV (Ryabukhin 1999); MDL URAL (Uhova 2001); MDL OB (Smetana 1967); MURM PROV (cRyv); NE EUR RU (Shilov 1975; Smetana 1976; Konakova and Kolesnikova 2017; cSme); N YENISS (Heyden 1880; Poppius 1909); S KURIL (Shibata et al. 2006); SW SIBER (Buhkalo et al. 2012); unspecified localities: ‘Ecosse et bords du lac Baikal’ (Fauvel 1874); ‘Baikal’ (Ganglbauer 1895); ‘Sibirien’ (Horion 1965); ‘Ural bor.; Fl. [maybe Finland] Pjosa’ (Smetana 1967); northern part of European Russia (Silfverberg 1992).

#### Quedius (Raphirus) fumatus (Stephens, 1833)

Fig. 14W–Y

The species is distributed in Europe and North Africa, and is most common in the western part of its distribution (Solodovnikov 2012b). In Russia, it is known only from its European part. *Quediusfumatus* can be found in leaf litter or other kinds of ground-based debris in deciduous forests, often in rotten logs or under bark (Legner and Moore 1977; Owen 2000); it has been also recorded from a cave (Outerelo et al. 1998).

Russia: KALIN PROV (Alekseev and Shapoval 2012); N CAUC (cRyv); NE EUR RU (Shilov 1975).

#### Quedius (Raphirus) gemellus Eppelsheim, 1889

Fig. 17S–U

*Quediusghilarovi* Coiffait, 1967: 405;

*Quediusparamerus* Coiffait, 1967: 411; Solodovnikov 2004: 225 (synonymy).

The species is endemic to the north-western Caucasus (south-western Russia and western Georgia) (Eppelsheim 1889; Solodovnikov 2004) where it is very common throughout its narrow distribution range. Usually it is found in leaf litter of forests from the foothills up to 1200–1500 m (Solodovnikov 2004; material examined here). Details about the taxonomy of this species can be found in Solodovnikov (2004).

Russia: N CAUC (Eppelsheim 1889; Roubal 1911; Gridelli 1924; Coiffait 1967; Boháč 1986; Solodovnikov 1998, 2004; cKur; cRyv; cSme; LUOMUS; LUOMUS; ZIN).

#### [Quedius (Raphirus) humeralis Stephens, 1832]

Fig. 17M–O

*Quediushumeralis* is a widespread West Palearctic species known from Europe, North Africa, and the Middle East (Herman 2001). The literature-based record from Middle Asia (Eppelsheim 1892) was not confirmed in our recent revision (Salnitska and Solodovnikov 2018b). We have not seen any specimens from Russia, which suggests that all literature records below are based on misidentifications. The species is not common and can be found in leaf litter and different types of ground based debris (Solodovnikov 2012b).

Russia (doubtful records): BURYAT REP (Dorzhieva and Khobrakova 2014; Dorzhieva 2015); IRKUTSK PROV (Shavrin et al. 1999); KRSNYRSK (Lopatina 2014); KUZN ALTAI (Babenko 1991); MDL VOLGA (Matveev 2011); N CAUC (Roubal 1911); unspecified locality: ‘Central and south-western regions’ (Potockaja 1967).

#### Quedius (Raphirus) humosus Solodovnikov, 2005

Fig. 18M–O

The species was described from Abkhazia (Solodovnikov 2005). Here we record it for the first time from adjacent Krasnodar Territory in Russia. Specimens from the original description were collected by pitfall traps at low elevations in the mountains (Solodovnikov 2005).

Russia: N CAUC (Solodovnikov 2005; ZIN).

#### Quedius (Raphirus) jenisseensis Sahlberg, 1880

Figs 7C, 8C, 17A–C

*Quediusjenisseensis* is an arctoboreal Eurasian species that is widely distributed in several northern-European regions of Russia through Sakha Republic and Zabaikalsky territory, to Primorsky Territory in the Far East. The species can be found in forest leaf litter, moss, and different types of ground debris, but usually it prefers moist habitats around rivers and streams (Smetana 1976, 1995; Smetana and Shavrin 2018). In the southern areas of its range it can be found at rather high elevations, up to 2450 m, around alpine meadows (material examined here).

Russia: ALTAI REP (NHMD; cRyv); BURYAT REP (Smetana 1995; cSha); IRKUTSK PROV (Shavrin et al. 1999; Shavrin et al. 2001; Smetana and Shavrin 2018; ISEA; cRyv); KRSNYRSK (Sahlberg 1880; Veselova and Ryvkin 1991; Rybalov et al. 2000; cRyv); KUZN ALTAI (ZMMU); LWR OB (Striganova and Porjadina 2005); MDL OB (Smetana 1976); N YENISS (Sahlberg 1880; Smetana and Shavrin 2018); NE EUR RU (Kolesnikova 2012; Kolesnikova and Konakova 2010, 2017; NHMD); NE YAKUT (Poppius 1909); NEN–NVZEM (Smetana 1976; Kolesnikova 2015; Smetana and Shavrin 2018); NW YAKUT (Smetana 1976); PRIM TERR (Smetana 1976); S YAKUT (CNC; ISEA); SW SIBER (Buhkalo et al. 2012); TUVA REP (cRyv); ZABAIK TERR (Shavrin 2000; cSha).

#### Quedius (Raphirus) korgeanus Fagel, 1968

Figs 6F, 16D–F

*Quediussvanetianus* Coiffait, 1969: 53;

*Quediusorophilus* Drugmand, 1988: 202; Solodovnikov 2004: 234 (synonymy).

*Quediuskorgeanus* is a widely distributed species in the mountains of northern Turkey and Transcaucasia (Solodovnikov 1998, 2004). In Russia, it is known from the north-western Caucasus with the north-easternmost records reaching Karachaevo-Cherkessia. This polytopic montane species can be found at 1400–2500 m, from forests up to alpine meadows. *Quediuskorgeanus* occurs in forest leaf litter and other ground-based debris, under stones, in moss around streams and at edges of snowfields, etc. (Solodovnikov 2004).

Russia: N CAUC (Solodovnikov 1998, 2004; ZIN).

#### [Quedius (Raphirus) lateralis (Gravenhorst, 1802)]

Fig. 18V–X

*Quediuslateralis* is widely distributed in Europe and Asia Minor (Solodovnikov 2012b). It is very similar to the more south-eastern species *Q.suramensis*, but the south-eastern distributional border for *Q.lateralis* is unclear and thereby it is unknown whether these species could be sympatric. Nevertheless, there are two records of *Q.lateralis* from Russia, but both are questionable. The first, from the western Caucasus (Rouball 1911) could easily be a misidentified *Q.suramensis*, even though the author recorded *Q.suramensis* from the same locality as well. The second record is general from the “Identification key of the rove beetle larvae of the European part of USSR” (Potockaja 1967). We did not find any specimens from Russia in collections, which suggests that this species does not occur here.

Russia (doubtful records): N CAUC (Roubal 1911); unspecified locality: ‘Palearctic, decaying plant residues’ (Potockaja 1967).

#### Quedius (Raphirus) lgockii Roubal, 1911

Figs 7F, 18G–I

*Quediuslgockii* is a rare montane species endemic to the north-western Caucasus and hitherto known from south-western Russia and western Georgia only (Solodovnikov 2004). Usually it can be found under stones at rather high elevations around 1900–2700 m (Roubal 1911; Solodovnikov 2004; Assing 2016).

Russia: N CAUC (Roubal 1911; Boháč 1980; Solodovnikov 1998, 2004; Assing 2016; LUOMUS; ZIN).

#### Quedius (Raphirus) limbatus (Heer, 1839)

Fig. 17G–I

*Quediuslimbatusponticus* Korge, 1964: 121;

*Quediuslimbatuserdciyasicus* Korge, 1971: 55;

*Quediuspotockajae* Coiffait, 1967: 414;

*Quediusledouxi* Coiffait, 1977: 138; Solodovnikov 2002a: 147 (synonymy).

*Quediusscheerpeltzianus* Fagel, 1968: 195; Assing 2018: 163 (synonymy).

This is one of the most common species within the subgenus Raphirus in the West Palearctic, where it is distributed from Europe to Middle Asia (Herman 2001; Solodovnikov 2012b; Salnitska and Solodovnikov 2018b). It is also widespread in Russia, recorded from all over its European part to Transbaikalia. *Quediuslimbatus* can be found in various ground-based debris from lowland forests up to subalpine meadows and edges of snowfields (Solodovnikov 2012b; material examined here).

Russia: ALTAI REP (cRyv); BURYAT REP (Shavrin 1998; cSha); CN EUR RU (Semenov 2009; Semionenkov et al. 2015; cKur; ZMMU; ZIN); CRIM REP (Koval 1961; Gusarov 1989; Turbanov et al. 2016; ZIN); CS EUR RU (cRyv); E CAUC (Coiffait 1967; Khachikov 1998); EUR S–TAIGA RU (Dedykhin et al. 2015); IRKUTSK PROV (Heyden 1896; Shavrin 2001; cSha; LUOMUS); KRSNYRSK (cRyv); LWR VOLGA (Grebennikov 2001); MDL OB (Sahlberg 1880; cRyv; ZIN); MDL URAL (Uhova 2001; cRyv); MDL VOLGA (Solodovnikov et al. 2002; Shulaev 2008; Shulaev and Bogdanov 2008; ZIN); MURM PROV (cRyv); N CAUC (Reitter 1888; Coiffait 1967, 1978; Bolov 1969a, b; Khachikov 1998; Solodovnikov 1998; Iljina and Khachikov 2000; Solodovnikov 2002a; Knysh and Solodovnikov 2004; Aiydov 2014, 2015; Pushkin 2015, 2016; Pushkin and Maksimova 2014; Pushkin and Minaev 2015a; cRyv; ZIN); NE EUR RU (ZIN); NW EUR RU (Seidlitz 1875; ZIN); N YENISS (Sahlberg 1880); S URAL (cRyv); SW SIBER (Sahlberg 1880; Striganova and Porjadina 2005; Buhkalo et al. 2012); VOLGO-DON (Khachikov 1998; Grebennikov 2001; Pushkin 2015, 2016; Arzanov et al. 2016); ZABAIK TERR (Shavrin 2000; cSha); unspecified locality: ‘Russie’ (Fauvel 1874); ‘weit nach dem Kaukasus’ (Smetana 1962); ‘Kaucasus’ (Horion 1965); northern part of European Russia (Silfverberg 1992).

#### Quedius (Raphirus) lucidulus Erichson, 1839

Figs 7A, 16K–M

The species is widespread and common in Europe and also recorded from Asia Minor (Coiffait 1978; Ghahari et al. 2009; Samin et al. 2011). Records from the Caucasus require confirmation. In Russia it is known only from its European part. Usually *Q.lucidulus* occurs in various ground-based debris from lowlands up to the subalpine zone (Solodovnikov 2012b).

Russia: CN EUR RU (Semenov 2010; Semionenkov et al. 2015); KALIN PROV (Seidlitz 1875); W EUR RU (Horion 1965); unspecified localities: ‘weit nach dem Kaukasus’ (Smetana 1962); ‘Caucase’ (Coiffait 1978); northern part of European Russia (Silfverberg 1992).

#### [Quedius (Raphirus) maurorufus (Gravenhorst, 1806)]

Fig. 17Y–AA

*Quediusrichteri* Korge, 1966: 60; Solodovnikov 2012a: 36 (synonymy).

The species is common in Europe, where it is more abundant in the central and southern regions (Solodovnikov 2012b). The absence of this common European species in the better sampled European part of Russia make the few literature records from Eest Russia highly ambiguous. *Quediusmaurorufus* can be found in forests and open landscapes in various ground based debris.

Russia: IRKUTSK PROV (Shavrin 2001); KUZN ALTAI (Babenko 1991); unspecified locality: “Caucase” (Fauvel 1874).

#### [Quedius (Raphirus) nemoralis Baudi de Selve, 1848]

Fig. 17BB–DD

*Quediussafaensis* Fagel, 1968: 8;

*Quediussafaensisormanus* Fagel, 1971: 129;

*Quediusnemoraliserinci* Korge, 1971: 55; Assing 2018: 162 (synonymy).

This is a widespread species in Europe and in Asia Minor (Solodovnikov 2012b). The old record from the Caucasus (Horion 1965) was apparently based on a misidentification. In Russia, it is known only from its northern and central European parts, based on scarce literature records. *Quediusnemoralis* can be found in wet ground-based habitats, often on sandy soils (Solodovnikov 2012b).

Russia: CN EUR RU (Horion 1965; Semionenkov et al. 2015); NW EUR RU (Horion 1965); unspecified localities: ‘Kaucasus’ (Horion 1965); northern part of European Russia (Silfverberg 1992).

#### Quedius (Raphirus) nigriceps Kraatz, 1857

Fig. 17D–F

The species is known from Europe where it is more abundant in the west; it is not recorded from North Africa (Solodovnikov 2012b). In Russia, it is known from its European part and Irkutsk Province based on a few literature records. *Quediusnigriceps* occurs in wet ground-based habitats in forests and is also recorded from mole nests (Nowosad 1990).

Russia: CN EUR RU (Semionenkov et al. 2015); IRKUTSK PROV (Shavrin et al. 1999); MDL VOLGA (Shulaev and Bogdanov 2008).

#### Quedius (Raphirus) nitipennis (Stephens, 1833)

Fig. 15J–L

*Quediussacuminatuskhnzoriani* Coiffait, 1967: 423; Solodovnikov 2004: 235 (synonymy).

*Quediusnitipennis* is a West Palearctic species, known from Europe, North Africa, and Asia Minor (Herman 2001; Solodovnikov 2012b). In Russia, it is not common and known from scattered literature records from its European part including Northern Caucasus. Very old records from Irkutsk province (Fauvel 1874, 1875) are not reliable. *Quediusnitipennis* usually can be found at different elevations from lowlands up to 2700 m, where it inhabits wet ground-based debris around water bodies or edges of snowfields (Solodovnikov 2012b). In the southern edge of its distribution range, the species occurs at high elevations (Horion 1965; Solodovnikov 2004).

Russia: CN EUR RU (Semionenkov et al. 2015); EUR S–TAIGA RUS (Anciferov and Polezhaeva 2014a, b); IRKUTSK PROV (Fauvel 1874, 1875; Shavrin 2001); N CAUC (Bolov 1969a, b; Solodovnikov 1998, 2004; ZIN); NE EUR RUS (Shilov 1975); unspecified locality: ‘west and mittelsibirien’ (Horion 1965); ‘northern part of European Russia’ (Silfverberg 1992).

#### Quedius (Raphirus) obliqueseriatus Eppelsheim, 1889

Figs 8A, 18G–L

This is endemic species to the north-western Caucasus and usually can be found in forest leaf litter from the foothills up to 1950 m (Solodovnikov 2004; material examined here). Records from Turkey and Iran (Korge 1964, 1971) are based on misidentifications.

Russia: N CAUC (Eppelsheim 1889; Roubal 1911; Jablokov–Khnzorian 1975; Boháč 1980; Khachikov 1998; Solodovnikov 1998, 2004; Knysh and Solodovnikov 2004; Assing 2016; cRyv; cSme; CNC; ZMMU; ZIN).

#### Quedius (Raphirus) omissus Coiffait, 1977

Fig. 15G–I

This montane species is known only from the north-western Caucasus of Russia and from the north-eastern Turkey (Assing 2017). *Quediusomissus* can be found at subalpine and alpine meadows around 1900–2700 m elevation, usually near streams or under stones (Solodovnikov 2002a; Assing 2016).

Russia: N CAUC (Coiffait 1977; Solodovnikov 1998, 2002a; LUOMUS; ZIN).

#### Quedius (Raphirus) paraboops Coiffait, 1975

Fig. 15P–R

*Quediusparaboops* is widely distributed in Siberia from Middle Ob region in the west to Magadan province in the east. We were able to study a female specimen from the *Q.boops*-group collected on Sakhalin Island and, since the very similar species *Q.boops* and *Q.boopoides* do not occur in this region, presumably this specimen belongs to *Q.paraboops*. The species can be found in wet ground based debris in forests and open landscapes, and also in moss and under stones (Ryabukhin 1999; material examined here). Additional remarks on this species can be found in the introductory Taxonomy section.

Russia: AMUR PROV (Smetana and Shavrin 2018); BURYAT REP (Coiffait 1975; cRyv); IRKUTSK PROV (Smetana 1976; Shavrin et al. 1999, 2001; Smetana and Shavrin 2018; cRyv); KRSNYRK (Veselova and Ryvkin 1991); LWR AMUR (cRyv); MAGADAN PROV (Ryabukhin 1999); MDL OB (Smetana 1976); N YENISS (Smetana 1978b); NW YAKUT (Smetana 1976; CNC); S YAKUT (Smetana 1976); ZABAIK TERR (Shavrin 2000; Smetana and Shavrin 2018; cRyv; cSha).

#### Quedius (Raphirus) persimilis Mulsant & Rey, 1876

Fig. 15A–C

*Quediuscorion* Tottenham, 1948: 258;

*Quediusmallius* Tottenham, 1948: 256; Duff et al. 2012: 54 (synonymy).

The species is widely distributed throughout Europe and is most common in central Europe (Solodovnikov 2012b). In Russia it is known only from its European part. *Quediuspersimilis* is confined to dry and sunny open biotopes, found in ground-based debris or pine leaf litter (Solodovnikov 2012b).

Russia: MURM PROV (Koryakin et al. 2004); N CAUC (Solodovnikov 1998; Knysh and Solodovnikov 2004); NE EUR RU (Kolesnikova and Konakova 2010; Konakova and Kolesnikova 2017); NW EUR RU (ZIN).

#### [Quedius (Raphirus) picipes (Mannerheim, 1830)]

Fig. 16W–Y

The species is widely distributed throughout the West Palearctic where it was recorded from Europe, North Africa, and Asia Minor (Lucas 1846; Fauvel 1874; Solodovnikov 2012b). Its presence in Russia and especially in South-West Siberian region (Voitenkova 2016) is questionable, because most of the records are from old literature only (Hochhuth 1862; Potockaja 1967; Silfverberg 1992). *Quediuspicipes* usually can be found in leaf litter or sometimes in various other organic decaying matter like mushrooms or carrion, or even in mole nests (Nowosad 1990; Owen 2000; Solodovnikov 2012b).

Russia: SW SIBER (Voitenkova 2016); unspecified localities: ‘Russlands’ (Hochhuth 1862); ‘widespread in Europe’ (Potockaja 1967); northern part of European Russia (Silfverberg 1992).

#### Quedius (Raphirus) riparius Kellner, 1843

Figs 7B, 16T–V

*Quediusriparius* is a ripicolous species that usually occurs at medium elevations in the mountains of Central and Southern Europe, Caucasus, Asia Minor, and Near East (Solodovnikov 2012b). In Russia this species is known only from the Western Caucasus. Generally, *Q.riparius* prefers wet debris around flowing water: small rivers, streams, waterfalls, often in moss (Herman 1911; material examined here).

Russia: N CAUC (Gridelli 1924; Solodovnikov 1998; cGon; cSme; ZIN); unspecified localities: ‘Caucasus’ (Ganglbauer 1895); ‘Caucase’ (Coiffait 1978).

#### Quedius (Raphirus) ryvkini Smetana, 2018 in Smetana and Shavrin (2018)

Fig. 16G–H

*Quediusryvkini* is a newly described species from Sikhote-Alin Mountains in Primorsky Territory of Russia that so far is known only from the original description (Smetana and Shavrin 2018). The bionomics is unknown; type specimens were taken from window traps.

Russia: PRIM TERR (Smetana and Shavrin 2018).

#### Quedius (Raphirus) scintillans (Gravenhorst, 1806)

Fig. 16N–P

*Quediusscintillans* is a common West Palearctic species distributed from Europe and North Africa to Middle Asia (Herman 2001; Solodovnikov 2012b; Salnitska and Solodovnikov 2018b). In Russia, it is known only from its European part. The species occurs in forests and open landscapes at low elevations, usually in various ground-based debris and often in hay (Solodovnikov 2012b).

Russia: CN EUR RU (Semionenkov et al. 2015); CRIM REP (Gusarov 1989); MDL VOLGA (Goreslavets et al. 2002; Goreslavets 2016b); N CAUC (Khachikov 1998; ZIN); NE EUR RU (Shilov 1975); VOLGO–DON (Khachikov 1998, 2012; Arzanov et al. 2016); unspecified locality: ‘Caucase’ (Fauvel 1874).

Russia: N CAUC (ZIN).

#### Quedius (Raphirus) semiaeneus (Stephens, 1832)

Fig. 14Q–S

The species is widely distributed in the West Palearctic: Europe, North Africa, and Asia Minor (Herman 2001). In Russia, it is known from the northern regions of its European part, but based only on literature records. *Quediussemiaeneus* usually prefers open and dry landscapes, where it occurs in various ground-based debris (Solodovnikov 2012b).

Russia: NE EUR RU (Kolesnikova and Taskaeva 2003; Konakova and Kolesnikova 2017); NEN–NVZEM (Kolesnikova 2015); NW EUR RU (Kolesnikova 2008).

#### Quedius (Raphirus) semiobscurus (Marsham, 1802)

Fig. 16A–C

*Quediusacuminatuskhnzoriani* Coiffait, 1967: 423; Solodovnikov 2004: 235 (synonymy).

*Quediussemiobscurus* is a common West Palearctic species that occurs in Europe, North Africa, and the Middle East (Herman 2001; Anlaş and Newton 2010; Assing 2016). In Russia, it is recorded only from lower elevations of the Caucasus (Solodovnikov 1998, 2004). Usually it can be found at low elevations below 500 m, where it occurs in ground-based debris of both open and forested landscapes (Solodovnikov 2012b).

Russia: E CAUC (Khachikov 1998; Solodovnikov 2004; ZIN); N CAUC (Solodovnikov 1998, 2004).

#### Quedius (Raphirus) sublimbatus Mäklin, 1853

Figs 7D, 17P–R

*Quediussublimbatus*, described from North America, is a Holarctic species that is more common in the northern parts of its distribution, while in the southern areas it occurs in the mountains. Apparently, it has an arctoboreoalpine type of distribution (Herman 2001; Ryabukhin 1999). In Russia, *Q.sublimbatus* is distributed from Murmansk Province to Kamchatka peninsula and is most common in northern Siberia and Far East. The species prefers wet habitats and usually can be found in various plant debris, mosses and lichens near water (Ryabukhin 1999; material examined here).

Russia: BURYAT REP (Smetana 1995; Shavrin 2000); CHUKOTKA (Ryabukhin 1999); IRKUTSK PROV (Gridelli 1924; Shavrin and Anischenko 1997; Shavrin et al. 1999; cSha); KAMCHATKA (Bernhauer 1926; Smetana 1976, 1978; Ryabukhin 1999, 2008; Lobkova and Semenov 2005; ZIN); KRSNYRSK (Smetana 1976; cRyv; ZIN); LWR AMUR (cRyv); MAGADAN PROV (Ryabukhin 1999); MDL URAL (cRyv); MURM PROV (Smetana 1967); N YENISS (Bernhauer 1926; Smetana 1967, 1978b); S KURIL (Shibata et al. 2006); ZABAIK TERR (Coiffait 1967; cRyv); unspecified localities: ‘région du Baïkal, Irkutsk’ (Fauvel 1875); ‘Baikalgebiete’ (Bernhauer 1902); northern part of European Russia (Silfverberg 1992).

Notes: There is some controversy whether *Quediusarcticus* Munster, 1921 is a synonym of *Q.sublimbatus*, or a valid species. *Quediusarcticus* was described from Norway (Munster 1921) and recorded mainly from northern Europe (Munster 1923; Palm 1963; Coiffait 1978), but also from Siberia, Mongolia (Smetana 1963, 1967, 1975 etc.) and North America (Smetana 1965, 1971 etc.). Smetana (1965) synonymized *Q.arcticus* with *Q.sublimbatus* because he considered their aedeagi identical. Also he indicated that for the material from northern Europe and Mongolia as well. It remains unclear from his publication though, whether he examined the type material of *Q.arcticus*. In spite of Smetana’s (1965) synonymy, Coiffait (1978) still used *Q.arcticus* as a valid name without any comments, while Veselova and Ryvkin (1991) explicitly reinstated *Q.arcticus* from synonymy. They mentioned that the Palearctic specimens, which they attributed to *Q.arcticus*, differ from the North American *Q.sublimbatus* in the structure of paramere. But it remains unclear whether Veselova and Ryvkin (1991) actually examined the North American specimens of *Q.sublimbatus* as well, or based their idea of that species only on Smetana (1975). And obviously they did not examine any type material too. Smetana (1995) again insisted on the synonymy of both species, contrary to Coiffait (1978), but he overlooked and did not comment the publication by Veselova and Ryvkin (1991). Currently *Q.arcticus* is listed as a junior synonym of *Q.sublimbatus* in all modern catalogues. We were able to examine rather wide material from Eurasia and North America and did not notice any hiatus between samples from respective continents. Moreover, the variability seen across the Holarctic material displays a pattern more complex than the division between North American and Eurasian populations, as claimed in Veselova and Ryvkin (1991). Additinally, the specimens of *Q.sublimbatus* from Siberia and Russian Far East are mostly wingless, usually without palisade fringe on tergite VII and with short, but differently sized wings and elytra, while the specimens from Europe and North America are winged. Interestingly, one specimen from Lower Amur region in Far East had fully developed wings.

We suspect that with a closer study including molecular analysis of the broad material and study of types, a wide-spread and wing polymorphic Holarctic *Q.sublimbatus* may not be the case, whereas species borders may not necessarily coincide with the border between North America and Eurasia as hypothesized by Veselova and Ryvkin (1991). For the time being and in agreement with the majority of papers, we follow Smetana’ (1965) concept of the wide-spread *Q.sublimbatus* with *Q.arcticus* as its junior synonym.

#### Quedius (Raphirus) suramensis Eppelsheim, 1880

Fig. 18S–U

*Quediusgrouziacus* Coiffait, 1969: 45; Solodovnikov 2002a: 142 (synonymy).

The species is distributed in Western Caucasus, Transcaucasia, and northern Turkey (Herman 2001; Solodovnikov 2004; Özgen et al. 2016). In Russia, it is mainly known from Northern Caucasus region, but recently it was recorded from Middle Volga region too. Mostly, *Quediussuramensis* is confined to mountain forests at elevations from 200 to 1800 m, where it can be found in leaf litter, rotten mushrooms, faeces of brown bear (Solodovnikov 2002a) and even in rodent burrows (Lyayster 1967). Detailed information about this species can be found in Solodovnikov (2002a).

Russia: N CAUC (Reitter 1888; Roubal 1911; Gridelli 1924, 1938; Boháč 1986; Khachikov 1998; Solodovnikov 1998, 2002a; Knysh and Solodovnikov 2004; Pushkin and Maksimova 2014; Pushkiv and Minav 2015a; Pushkin 2015, 2016; cKur; cRyv; cSme; FMNH; ZMMU; ZIN); MDL VOLGA (Khachikov 2017).

#### Quedius (Raphirus) suturalis Kiesenwetter, 1845

Fig. 17J–L

*Quediusobscuriceps* Coiffait, 1967: 404; Solodovnikov 2002a: 149 (synonymy).

*Quediusmerlini* Drugmand & Bruge, 1991: 192; Solodovnikov 2012: 39 (synonymy).

*Quediustroglophilus* Coiffait, 1969: 46.*Quediushumeralisanatolicus* Korge, 1964: 119; Assing 2018: 163 (synonymy).

*Quediussuturalis* is a widely distributed West Palearctic species but it is not recorded from North Africa (Herman 2001; Solodovnikov 2012b). In Russia, it is known only from Northern Caucasus region, although earlier records of *Q.humeralis* may in fact belong to this species due to nomenclatural changes. The species can be found in the mountains up to the alpine zone; it prefers moist microhabitats such as leaf litter and moss (Solodovnikov 2012b; material examined here).

Russia: N CAUC (Khachikov 1998; Solodovnikov 2002a; cKur; cSme; ZIN); unspecified locality: ‘Russie’ (Fauvel 1874); ‘Caucase’ (Coiffait 1967).

#### Quedius (Raphirus) umbrinus Erichson, 1839

Fig. 17V–X

*Quediusumbripennis* Gridelli, 1924: 113; Solodovnikov 2002a: 150 (synonymy);

*Quediuscyanescens* Mulsant & Rey, 1876: 727;

*Quediusbulgaricus* Scheerpeltz, 1937: 219;

*Quediuscyprusensis* Last, 1955: 251;

*Quediusfreyi* Scheerpeltz, 1956: 1102;

*Quediusmaronitus* Coiffait, 1963: 410;

*Quediusgueorguievi* Coiffait, 1967: 399; Assing 2018: 151 (synonymy).

*Quediuskuboni* Štourač, 1998: 15; Assing 2019: 2 (synonymy).

*Quediusumbrinus* is a widely distributed West Palearctic species known from Europe, Middle East, and Middle Asia, but not recorded from North Africa (Herman 2001; Assing 2013, 2017b; Salnitska and Solodovnikov 2018b). In Russia, it is most common throughout the European part, becoming more rare towards the east; easternmost records are from Krasnoyarsk and South-Western Siberia (material examined here). This species prefers forested landscapes and usually can be found in rather wet habitats around water in leaf litter, moss, or other ground-based debris.

Russia: CN EUR RU (Semionenkov et al. 2015; cRyv; ZMMU; ZIN); CRIM REP (Gusarov 1989; cKur; ZIN); E CAUC (Khachikov 1998; Solodovnikov 2002a; ZIN); EUR S–TAIGA RU (Dedykhin et al. 2005); IRKUTSK PROV (Shavrin et al. 1999; cSha); KRSNYRSK (cRyv); MDL URAL (Belskaya and Kolesnikova 2011); MDL VOLGA (Goreslavets et al. 2002; Matveev 2011; ZIN); N CAUC (Roubal 1911; Bolov 1969a; Khachikov 1998; Solodovnikov 1998, 2002a; Knysh and Solodovnikov 2004; ZMMU; ZIN); NE EUR RU (Kolesnikova and Taskaeva 2003; ZIN); NW EUR RU (Poppius 1908; Kolesnikova 2008; ZIN); SW SIBER (ZIN); VOLGO-DON (Grebennikov 2001; Arzanov et al. 2004; Kovalev 2011); unspecified localities: ‘Russie’ (Fauvel 1874); ‘Kaukasus’ (Horion 1965); ‘widespread’ (Potockaja 1967); northern part of European Russia (Silfverberg 1992).

#### Quedius (Raphirus) vulneratus Gemminger & Harold, 1868

Figs 7E, 18A–C

*Quediusabkasicus* Coiffait, 1963: 410; Solodovnikov 2002a: 153 (synonymy).

The species is widely distributed in the Caucasus from its north-western part to eastern Transcaucasia, and also occurs in northern Turkey (Korge 1964, 1971; Solodovnikov 1998, 2002a). *Quediusvulneratus* can be found in moist ground based debris including rotten mushrooms and animal faeces (Solodovnikov 2002a), and under stones at the edges of snowfields. It is recorded from the foothills at 300–400 m up to the subalpine zone at 2000–2400 m elevation.

Russia: E CAUC (Solodovnikov 2002a; cRyv); N CAUC (Reitter 1888; Eppelsheim 1889; Roubal 1911; Boháč 1986; Khachikov 1998; Solodovnikov 1998, 2002a; Knysh and Solodovnikov 2004; cRyv; cSme; ZIN).

### Subgenus Velleius Leach, 1819

#### Quedius (Velleius) dilatatus Leach, 1819

Fig. 18Y–AA

The species is distributed throughout the Palearctic, from Europe to the Far East including Japan, eastern China, southern Korea, and Russia (Herman 2001; material examined here). It is associated with nests of *Vespacrabro*, where its larvae feed on larvae of Diptera in the nest debris. Details on biology and the developmental stages of *Q.dilatatus* can be found in Strassen (1957).

Russia: CN EUR RU (Semionenkov et al. 2015; Ruchin 2017; ZIN); CS EUR RU (Khachikov 1998; Ruchin and Egorov 2015; Ruchin 2017; ZIN); IRKUTSK PROV (Shavrin 2001; ZIN); LWR AMUR (cRyv); MDL VOLGA (Goreslavets et al. 2002; Shulaev 2008; ZIN); N CAUC (Khachikov 2017; Miroshnikov 2018); NW EUR RU (Seidlitz 1874; ZIN); PRIM TERR (ZIN; cRyv); S URAL (ZIN); SW SIBER (Buhkalo et al. 2012); VOLGO–DON (Khachikov 2003); unspecified locality: ‘über Sibirien’ (Horion 1965); ‘widespread’ (Potockaja 1967); northern part of European Russia (Silfverberg 1992).

Notes: Overall, the subgenus Velleius comprises nine species distributed in China and Japan (Zhao and Zhou 2015; Smetana 2018) and only one species, *Q.dilatatus*, is widespread in the rest of the Palearctic from the Russian Far East to Europe. It seems possible, however, that broader sampling will reveal some of the Chinese or Japanese species in the Russian Far East.

### Incertae sedis

#### *Quediusfulvipennis Hochhuth, 1851

Hochhuth (1851) described *Q.fulvipennis* from the unclear locality “Dahuria” (historical region comprising modern Buryatia Republic, Zabaikalsky territory and Amur province) without either an explicit mention of the subgenus it belongs, or information on the type material. He mentioned that systematically *Q.fulvipennis* is related to *Q.molochinus*, but in size and proportions is similar to *Q.fulgidus*. According to the original description, the body length of *Q.fulvipennis* is 2 ‘lin’ [around 9–10 mm] and coloration of elytra is reddish-brown. From these characters and the original description it is difficult to infer even the subgenus to which this species may belong. Later, (Bernhauer and Schubert 1916; Gridelli 1924; Scheerpeltz 1933) *Q.fulvipennis* was included in catalogs and lists, but without examination of the type material. Therefore, the identity of this species remains unknown.

Russia: unspecified locality: “Dahuria” [historical region comprising modern Buryatia Republic, Zabaikalsky territory and Amur province] (Hochhuth 1851).

### *Quedius* of Russia, summary table

The summary Table 1 lists all species alphabetically using the same regions as in the annotated species list. Columns (regions), from left to right, are arranged geographically, roughly from north to south and from west to east. Also they are numbered from 1 to 40 (from left to right). These numbers are duplicated in the alphabetical list of the abbreviated regions in the section about geographic subdivisions of Russia, where all regions are defined. Each cell in the summary table is graphically coded to represent details about a respective distribution record. This table should facilitate visualizations of species distributions, abundance, and degree of knowledge about them.

Table abbreviation: Number of published records 1 (light grey), 2–10 (grey), 11+ (dark grey); T.L. – type locality; ? – doubtful records; number of specimens examined here 1 (🞅), 2–10 (🞇), 11+ (⚫).

**Table 1.** Summary list for *Quedius* species recorded from Russia. Species whose presence in the Russian fauna is strongly ambiguous are taken in square brackets.

	**KALIN PROV (1)**	**AMURM PROV (2)**	**KAREL REP (3)**	**NW EUR RU (4)**	**NEN– NVZEM (5)**	**NE EUR RU (6)**	**EUR S-TAIGA RU (7)**	**CN EUR RU (8)**	**CS EUR RU (9)**	**MDL VOLGA (10)**	**VOLGO- DON (11)**	**LWR VOLGA (12)**	**CRIM REP (13)**	**N CAUC (14)**	**E CAUC (15)**	**MDL URAL (16)**	**S URAL (17)**	**LWR OB (18)**	**MDL OB (19)**	**SW SIBER (20)**	**N YENISS (21)**	**KRSNYRSK (22)**	**KUZN ALTAI (23)**	**ALTAI REP (24)**	**TUVA REP (25)**	**IRKUTSK PROV (26)**	**BURYAT REP (27)**	**ZABAIK TERR (28)**	**NW YAKUT (29)**	**NE YAKUT (30)**	**S YAKUT (31)**	**CHU KOTKA (32)**	**MAGADAN PROV (33)**	**KAM CHATKA (34)**	**KHABA ROVSK (35)**	**AMUR PROV (36)**	**LWR AMUR (37)**	**SAKHA LIN (38)**	**S KURIL (39)**	**PRIM TERR (40)**
*Q.abdominalis* Eppelsheim, 1888														T.L.																										
*Q.aedilis* Smetana, 2018																																								T.L.
*Q.altaicus* Korge, 1962																							🞇																	
*Q.amplissimus Bernhauer, 1912													T.L.?																											
*Q.amurensis* Smetana, 2018																																				T.L.				
*Q.angaricus Coiffait, 1975																										T.L.														
*Q.balticus* Korge, 1960																																								
*Q.boopoides* Munster, 1923		🞇		🞇										🞇					🞇			🞅				🞇		🞅												
*Q.boops* (Gravenhorst, 1802)		🞇		🞇				🞇					🞇	🞇					🞇		🞇	🞇				🞇	🞇	🞇									🞅			
[*Q.brachypterus* Coiffait, 1967]														T.L.?	T.L.?																									
*Q.brevicornis* (Thomson, 1860)								🞇																																
*Q.brevis* Erichson, 1840				🞇			🞇	🞇			🞇										🞇					🞇					🞇						🞇		🞅	
*Q.centrasiaticus* Coiffait, 1969																								T.L.																
[*Q.cincticollis* Kraatz, 1857]																																								
*Q.cinctus* (Paykull, 1790)										🞅				🞇																										
*Q.citelli* Kirschenblatt, 1933																												T.L.												
*Q.conviva* Smetana, 2018																										T.L.														
*Q.cruentus* (Olivier, 1795)				🞇					🞇				🞇	🞇																										
*Q.curtipennis* Bernhauer, 1908				🞇						🞅			🞇																											
*Q.dilatatus* Leach, 1819				🞅				🞇	🞅	🞇						🞅										🞇											🞅		⚫	
*Q.edmundi* Coiffait, 1969														🞇																										
*Q.fasciculatus* Eppelsheim, 1886																												🞅								🞇	🞇		🞅	🞇
*Q.fellmani* (Zetterstedt, 1838)								🞇										🞇			🞇	🞇	🞇	🞇		⚫		⚫		🞅				⚫					🞇	🞇
*Q.fulgidus* (Fabricius, 1793)							🞅	🞇	🞅				🞇													🞅														
*Q.fuliginosus* (Gravenhorst, 1802)			🞇	⚫				⚫	🞇	🞇			🞇	⚫	🞇	🞇	🞅				🞇	🞇	🞇			🞇	🞇													
*Q.fulvicollis* (Stephens, 1832)		🞇			🞇	🞇															🞇	🞇				⚫	🞇							⚫						
*Q.fulvipennis Hochhuth, 1852																											T.L.?	T.L.?								T.L.?				
*Q.fumatus* (Stephens, 1833)														🞇																										
*Q.fusus* Cai & Zhou, 2015																																					🞇			
*Q.gemellus* Eppelsheim, 1889														⚫																										
[*Q.humeralis* Stephens, 1832]										?				?								?	?			?	?													
*Q.humosus* Solodovnikov, 2005														🞅																										
*Q.infuscatus* Erichson, 1840										🞅																														
*Q.invreae* Gridelli, 1924										🞇				🞇																										
*Q.japonicus* Sharp, 1874																																					?	?	?	?
*Q.jenisseensis* Sahlberg, 1880						🞇																⚫	🞇	🞇	🞇	🞇	🞇	🞇			🞇									
*Q.kamchaticus* Smetana, 1976																																								
*Q.koltzei Eppelsheim, 1887																																					T.L.			
*Q.korgeanus* Fagel, 1968														🞇																										
*Q.kvashei Khachikov, 2005											T.L.																													
[*Q.lateralis* (Gravenhorst, 1802)]														?																										
*Q.levicollis* Brulle, 1832																																								
*Q.lgockii* Roubal, 1911																																								
*Q.limbatus* (Heer, 1839)		🞇		⚫		🞇	🞅	⚫	🞇	🞇			⚫	⚫		🞇	🞇		🞇			🞇		⚫		🞇	🞇	🞇												
*Q.longicornis* Kraatz, 1857				🞅				🞇						🞅						🞅																				
*Q.lucidulus* Erichson, 1839																																								
*Q.lundbergi* Palm, 1973																									🞅															
[*Q.maurorufus* (Gravenhorst, 1806)]																																								
*Q.maurus* (Sahlberg, 1830)				🞇				🞇						🞅																										
*Q.meridiocarpathicus* Smetana, 1958													⚫	🞇																										
*Q.mesomelinus* (Marsham, 1802)	🞅			⚫		🞇		⚫		🞅						🞅													🞅											
*Q.microps* Gravenhorst, 1847																																								
*Q.minor* Hochhuth, 1849														🞇																										
*Q.molochinus* (Gravenhorst, 1806)		🞇	🞇	🞅		🞇		🞇		🞇				🞅			🞅				🞇	🞇																		
[*Q.nemoralis* Baudi de Selve, 1848]																																								
*Q.nigriceps* Kraatz, 1857																																								
[*Q.nigrocaeruleus* Fauvel, 1876]	?	?	?	?	?	?	?	?	?	?	?	?	?	?	?	?	?	?	?																					
*Q.nitipennis* (Stepehns, 1833)														🞇																										
*Q.obliqueseriatus* Eppelsheim, 1889														⚫																										
*Q.ochripennis* (Ménétriés, 1832)									🞇				🞇			🞅																								
*Q.ochropterus* Erichson, 1840																																								
*Q.omissus* Coiffait, 1977														🞇																										
*Q.paraboops* Coiffait, 1975																										⚫	🞇	⚫	🞇								⚫			
*Q.persimilis* Mulsant & Rey, 1876																																								
[*Q.picipes* (Mannerheim, 1830)]																																								
*Q.puncticollis* (Thomson, 1867)																																								
*Q.repentinus* Salnitska&Solodovnikov, 2018																								T.L.																
*Q.riparius* Kellner, 1843														⚫																										
*Q.roma* Solodovnikov & Hansen, 2016																																					T.L.			
*Q.ryvkini* Smetana, 2018																																								
*Q.scintillans* (Gravenhorst, 1806)														🞅																										
*Q.scitus* (Gravenhorst, 1806)				🞇				🞅						🞅																										
*Q.semiaeneus* (Stephens 1832)																																								
*Q.semiobscurus* (Marsham, 1802)															🞇																									
*Q.sofiri* Khachikov, 2005											T.L.																													
*Q.sublimbatus* Mäklin, 1853																						🞇				🞇		🞅						🞅			🞇			
*Q.subunicolor* Korge, 1961						🞅																																		
*Q.sundukovi* Smetana, 2003																																				🞇	🞇	🞇		
*Q.suramensis* Eppelsheim, 1880														⚫																										
*Q.suturalis* Kiesenwetter, 1845														⚫																										
*Q.tenellus* (Gravenhorst, 1806)								🞇						🞇										🞅		🞇	🞇						🞇	🞅		🞇	🞇			
*Q.tetrapunctatus Coiffait, 1977																																								
*Q.truncicola* Fairmaire & Laboulbène, 1856																																								
*Q.umbrinus* Erichson, 1839				🞅		🞇		⚫		🞇			🞇	⚫	🞇					🞇		🞅				🞇														
*Q.vexans* Eppelsheim, 1881													🞅																											
*Q.vicinus* Ménétriés, 1832																																								
*Q.vulneratus* Gemminger & Harold, 1868														⚫	🞇																									
*Q.xanthopus* Erichson, 1839			🞅	🞇				⚫		🞅						🞅																								

## Discussion

Based on the examination of ca. 3000 specimens of *Quedius* from Russia in the collections and 165 publications with their records, our review revealed 88 species of *Quedius* for the fauna of Russia, of which *Q.fusus*, *Q.humosus* and *Q.lundbergi* are recorded from the territory of Russia for the first time. On the contrary, analysis of literature and available material suggested that *Q.cincticollis*, *Q.humeralis*, *Q.lateralis*, *Q.maurorufus*, *Q.nemoralis*, Q.*nigrocaeruleus, Q.picipes*, and possibly a few other species in fact do not occur in Russia. Their records here are dubious and likely are based on misidentifications, something to check in the future through more thorough sampling. Some species earlier reported for Russia, like for example narrowly distributed Alpine species *Quediushaberfelneri* recorded from the European part of Russia by Horion (1965), definitely does not occur in Russia. *Quediusplancus* recorded from the Caucasus by Gridelli (1924) also seems an obvious misidentification. One species, *Q.brachypterus*, described from an uncertain locality indicated as ‘Caucasus’ and never recollected since then, most likely occurs in the non-Russian part of the Caucasus. As discussed in the ‘Taxonomy’ section and noted in detail in the Annotated Catalogue, the identities of some species need further taxonomic study, preferably involving modern methods of molecular species delimitation, because of subtle inter-specific differences and significant intra-specific variation. One good example is the *Q.boops* group. As can be seen from the records in the Annotated Catalogue and visual patterns in Table 1 and Figs 3, 4, our current knowledge of *Quedius* of Russia is still based on very scarce material.

Naturally, the European part of Russia was better sampled and studied, while only a few regions in eastern Russia received comparable attention, such as Kamchatka or Primorsky Territory. However, even in western Russia there are poorly known areas such as Kaliningrad Province. One can clearly see in Fig. 3 that biodiversity-rich areas of the southern Urals, Altai, Buryatia, or Amur regions remain very poorly explored, in fact hardly sampled at all. Figure 4 shows that the main diversity of *Quedius* is confined to the more humid and warm western and southern areas of Russia, while the seemingly poor faunas of the forested Amur Province or Northern Khabarovsk region are simply an artefact of limited sampling in, or lack of literature about, these areas. Such an uneven and overall poor sampling of leaf litter invertebrates across the vast territories of Russia limits our understanding of *Quedius* species distributions. Many species records in faunistic papers require validation by a thorough taxonomic study of their underlying material. Generally, a high quality sampling- and collections-building program is required for Russian *Quedius* and Staphylinidae as a whole. The large area, diverse geography, and relatively rich rove beetle fauna of Russia provide a unique opportunity to explore many questions of Palearctic biogeography. We hope our paper will stimulate further activities in this direction.

## Footnotes

(see also couplet 21 in the key to species of Raphirus)

Q.kvashei fits here too because we presume both species are conspecific; for details see Annotated Catalogue section

Externally, Q.lateralis, (aedeagus as in Fig. 18V–X), may fit here except for its different coloration of elytra, which are dark with yellow epipleura. The presence of Q.lateralis in Russia is questionable, for details see notes for Q.suramensis in the Annotated catalogue section.]
